# Drinking alkaline mineral water confers diarrhea resistance in maternally separated piglets by maintaining intestinal epithelial regeneration via the brain-microbe-gut axis

**DOI:** 10.1016/j.jare.2022.12.008

**Published:** 2022-12-17

**Authors:** Jian Chen, Bi-Chen Zhao, Xue-Yan Dai, Ya-Ru Xu, Jian-Xun Kang, Jin-Long Li

**Affiliations:** a College of Veterinary Medicine, Northeast Agricultural University, Harbin 150030, PR China; b Key Laboratory of the Provincial Education Department of Heilongjiang for Common Animal Disease Prevention and Treatment, Northeast Agricultural University, Harbin 150030, PR China; c Heilongjiang Key Laboratory for Laboratory Animals and Comparative Medicine, Northeast Agricultural University, Harbin 150030, PR China

**Keywords:** Alkaline mineral water, Diarrhea, Brain-microbe-gut axis, Intestinal epithelial regeneration, Gut microbiota

## Abstract

**Introduction:**

Diarrhea has the fourth-highest mortality rate of all diseases and causes a large number of infant deaths each year. The maternally separated (MS) piglet (newly weaned piglet) is an excellent model to investigate the treatment of diarrhea in infants. Drinking alkaline mineral water has the potential to be therapeutic in gastrointestinal disorders, particularly diarrhea, but the supporting evidence from system studies and the mechanisms involved have yet to be reported.

**Objectives:**

This study aims to determine whether drinking alkaline mineral water confers diarrhea resistance in MS piglets under weaning stress and what the fundamental mechanisms involved are.

**Methods:**

MS piglets were used to create a stress-induced intestinal disorder-diarrhea susceptibility model. A total of 240 MS piglets were randomly divided into two groups (6 pens/group and 20 piglets/pen). IPEC-J2 cell line was used for in vitro evaluation. An alkaline mineral complex (AMC) water was employed, and its effect on the hypothalamus-pituitary-adrenocortical (HPA) axis, gut microbes, gut morphology, and intestinal epithelial cell (IEC) proliferation and differentiation were investigated using a variety of experimental methodology.

**Results:**

AMC water reduced diarrhea rate in MS piglets by inhibiting the HPA axis, ameliorating gut microbiota structure, and stimulating IEC proliferation and differentiation. Apparently, the brain-microbe-gut axis is linked with AMC water conferring diarrhea resistance in piglets. Mechanistically, AMC water decreased stress hormones (COR and Hpt) secretion by suppressing HPA axis, which then increased the abundance of beneficial gut microbes; accordingly, maintained the proliferation of IEC and promoted the differentiation of intestinal stem cells (ISC) into goblet cell and Paneth cell by activating the Wnt/β-catenin signaling pathway. In the absence of gut microbiota (in vitro), AMC activated the LPS-induced Wnt/β-catenin signaling inhibition in IPEC-J2 cells and significantly increased the number of Lgr5 + cells, whereas had no effect on IPEC-J2 differentiation.

**Conclusion:**

Drinking alkaline mineral water confers diarrhea resistance in MS piglets by maintaining intestinal epithelial regeneration via the brain-microbe-gut axis; thus, this study provides a potential prevention strategy for young mammals at risk of diarrhea.

## Highlights

• Drinking AMC water confers diarrhea resistance in piglets under weaning stress.
• Brain-microbe-gut axis is the target of AMC water for diarrhea therapy.
• Drinking AMC water alleviates weaning-induced intestinal injury in MS piglets.
• Drinking AMC water maintains intestinal epithelial regeneration in MS piglets.
• Gut microbe is a key factor in stimulating intestinal stem cell differentiation.

## Graphical abstract

The present study found that drinking alkaline mineral water confers diarrhea resistance in maternally separated piglets by maintaining intestinal epithelial regeneration via the brain-microbe-gut axis. The improvement in gut microbiota composition and function maintains intestinal epithelial regeneration by activating Wnt/β-catenin signaling to repair damaged intestinal mucosa and interrupted ISC differentiation. This study provides a potential prevention strategy for young mammals at risk of diarrhea.

**Figure:**
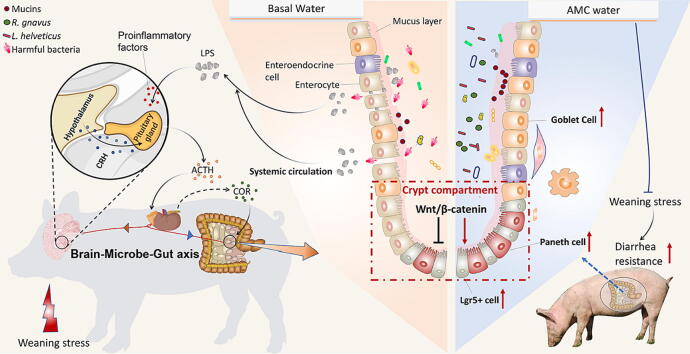

## Introduction

Diarrhea is the most prevalent gastrointestinal disease, at a given time, it affects up to 5 % of the population [1]. Diarrhea is one of the top causes of death among children under the age of three in poor nations, particularly in the second half-year [2]. According to World Health Organization (WHO) estimates in 2004, 1.5 million children worldwide died of severe diarrhea, primarily in low-income nations [3]. As a result, finding an effective approach to controlling diarrhea in children that most families can afford would be critical. Maternal separation, also known as weaning stress in piglets, is a popular stress paradigm. Exposure to weaning stress can result in intestinal injury and immune system malfunction in piglets, resulting in increased gut permeability, an inflammatory response, and a disordered gut microbiota structure, which then induces diarrhea [4], [5]. Hence, the maternally separated (MS) piglet is an excellent model for researching therapies for infant diarrhea. On the other hand, emerging evidence indicates that the brain and gut interact via the brain-microbe-gut axis, which may be prospective targets for the therapy of gastrointestinal disorders such as diarrhea and irritable bowel syndrome (IBS) [6]. However, the understanding of the brain-microbe-gut axis is currently limited, and relevant reports are scarce.

Water is the basic component of the diet and exerts an important physiological function in life activities. Drinking alkaline mineral water improved the quality of life of IBS patients with diarrhea-predominant, according to a clinical study report [7]. It is well known that natural alkaline mineral water generally contains Na, K, Zn and metasilicic acid, as well as some rare minerals such as germanium [8]. Coincidentally, it is these alkaline mineral elements that are lost during diarrhea [9], [10], leading to metabolic acidosis in piglets [11]. These minerals are not only the most numerous inorganic elements in the biota, but they are also the most researched due to their significant roles in biophysiological metabolism and catalysis [12]. The health and medical therapeutic effects of alkaline mineral complex (AMC) water have been widely studied and reported in human medicine, including improving cancer patients' quality of life, antioxidant effects, promoting intestinal health, treating intestinal inflammatory diseases and diarrhea [13], [14]. AMC water utilized in this study is an alkaline solution (pH 9.1), containing SiO_3_^2-^, Na^+^, K^+^, Zn^2+^, and Ge^4+^. As a non-specific immunostimulator, AMC water was used to promote pig growth and development as early as 2000 [15], [16], [17]. These minerals are essential for organ function (heart, brain, and gut), as well as various physiological functions such as digestion, immunological biosynthesis, and others [18]. In our previous study, we treated piglets with synthetic AMC water (same as this study) and found it significantly promoted piglet growth and intestinal barrier function [4]. However, whether AMC water confers diarrhea resistance in MS piglets, as well as the role of the brain-microbe-gut axis in stress-induced intestinal disturbances and diarrhea, is an important unanswered question.

Gut microbes interact with nearly all host organs, including the central nervous system (CNS), which regulates the connection between the cerebrum and the gut [19]. Moreover, the microbiota and brain have a bidirectional relationship that regulates neuronal, endocrine, metabolic, and immunological functions. Meanwhile, the gut microflora can interface with the brain via a variety of pathways and mediators, such as proinflammatory cytokines (IL-6, IL-1β, and TNF-α) [20], [21], [22]. The host's primary neuroendocrine system, hypothalamic–pituitaryadrenal (HPA) axis, as one of the systems that interacts closely with the gut microbiota, regulates multiple bodily processes in reaction to stress [23][62], [63], [64]. This interaction is critical; as numerous abnormalities of the microbiota-gut-brain axis are coupled with HPA axis dysfunction. Diarrhea pathogenesis and progression are complicated, involving gut barrier integrity, microbial composition, and the neuroendocrine system [23], [65], [67], [69]. The CNS regulates digestive system function through the sympathetic and parasympathetic nerves of the HPA axis and the autonomic nervous system (ANS) [19]. A study on an IBS-diarrhea mouse model showed that regulation of the brain-microbe-gut axis by downregulating corticotropin-releasing hormone (CRH) receptor 1 (CRHR1) expression and inhibiting HPA axis activity can ameliorate diarrhea symptoms [23]. However, the specific molecular mechanisms by which the HPA axis modulates the gut microbiota to improve gut health are unclear.

In the gut, intestinal epithelial cells (IEC) exhibit a vital effect in fluid transport, electrolyte equilibrium, and nutritional absorption, while also preventing bacteria, viruses, fungi, and toxins from entering host tissues and ensuring tissue homeostasis [24]. In mammals, IEC are renewed every 3–5 days [25]. Normally, IEC unceasingly regenerate through rapid proliferation and differentiation, from intestinal stem cells (ISC), mainly the Lgr5+ (leucine-rich-repeat-containing G-protein-coupled receptor 5+) ISC, to committed progenitors, and to specific IEC types (including Paneth cells, hormone-secreting enteroendocrine cells, and absorptive enterocytes, mucus-secreting goblet cells) that maintain the gut niche [26]. This prolonged regeneration, as a sign of intestinal homeostasis, is contingent on the maintenance of the equilibrium between ISC proliferation and differentiation. Once ISC niche homeostasis is disrupted, gut self-healing and barrier integrity will be devastated [27]. Evidence suggests that malnutrition due to diarrhea induces a pause in the regeneration of ISC, whereby the self-renewal and damage repair processes of the intestinal epithelium are inhibited. Of note, crypt epithelial cell proliferation and differentiation in the intestine is a dynamic system regulated by a tight signaling network; the main driving force of which is Wnt/β-catenin pathway [28]. Normally, a decrease in Wnt/β-catenin activity can suppress the expansion of ISC and induce villus shrinkage [29]. Hence, Wnt/β-catenin-mediated proliferation and differentiation of ISC may be a therapeutic target for intestinal dysbiosis. Given the importance of intestinal epithelial regeneration in maintaining intestinal homeostasis, we speculated that drinking AMC water promotes intestinal epithelial regeneration by activating the Wnt/β-catenin signaling pathway through the brain-microbe-gut axis, conferring diarrhea resistance in MS piglets.

Here, we investigated whether drinking alkaline mineral water could control weaning stress-induced diarrhea in MS piglets. From the role of brain-microbe-gut axis, our study demonstrated that drinking alkaline mineral water can reduce the secretion of stress hormones cortisol (COR) and haptoglobin (Hpt) by inhibiting the activity of the HPA axis, thereby increasing gut microbe diversity and the abundance of beneficial microbiotas, accordingly inducing the Wnt/β-catenin signaling activation to promote gut epithelial regeneration and specific IEC types differentiation, which in turn confer diarrhea resistance in MS piglets.

## Materials and methods

A more detailed “Material and Methods” is provided in the Supplementary Information.

### Ethics statement

The animal pain-reduction experiments were carried out following approval from the Guide for the Care and Use of Laboratory Animals at Northeast Agricultural University (NEAU), Harbin, China. The NEAU Animal Ethics Committee approved the experiments (No. NEAUEC202102415). All animal experiments should comply with the ARRIVE guidelines.

### MS piglets and treatment

The piglet model of maternal separation was employed as described previously [30]. A total of 240 MS piglets (large white × landrace × Duroc) weaned at 28 days of age were randomly placed into two groups (6 pens/group and 20 piglets/pen) depending on sex and body weight (9.35 ± 0.86 kg). All animals were provided with the same feed diet (NRC, 2012). According to our previous study, an AMC concentrate (Supplementary Table S1) containing Na^+^, K^+^, Zn^2+^, SiO_3_^2-^, Ge^4+^ and HCO_3_^–^ was synthesized and used in this experiment [4]. The piglets of control group (Con) were provided with basal water (pH 7.0), while the AMC group was supplemented with basal water plus 0.25 % AMC concentrate (pH 9.1). The mineral concentration of AMC water (Supplementary Table S2) employed in this trial was defined based on our previous study, which revealed that animals had the optimal growth rate and lowest diarrhea rates at this dose [4]. In a 15 days trial, all piglets had unlimited access to feed and water. All the raw materials for AMC were provided by Nail Biotechnology Co., ltd. (Beijing, China).

### Diarrhea incidence assessment

Consistent with our earlier work [31], the diarrhea index was graded based on stool status and water content: zero points for normal; one for soft feces; two for semisolid containing more than half water-like feces; and three for water-like feces. The diarrhea incidence was recorded according to the diarrhea index (fecal score ≥ 2).

### In vitro evaluation

Porcine intestinal epithelial cells (IPEC-J2 cell line, from Prof. Dong Na Lab., NEAU, Harbin, China) were cultured in an incubator at 37 °C and 5 % CO_2_ in DMEM/F12 supplemented with 10 % (v/v) fetal bovine serum (HyClone). All evaluations in vitro were performed during the logarithmic growth phase of IPEC-J2 cells.

A total of 100 μL of IPEC-J2 cells in suspension were plated in 96-well plates (2 × 10^4^ cells) and cultured for 24 h for cell viability assay. Then, AMC concentrate was added to the cells at concentrations of 0, 0.5, 1.0, 1.5, 2.0, 4.0, 6.0, and 8.0 mg/mL for 12 h, after that, 10 μL CCK-8 solution (Dojindo, Kumamoto, Japan) was added into each well and preserved at 37℃ for 2 h. By using a microplate reader, the absorbance was measured at 450 nm. Cell viability in AMC treatment was expressed as a proportion of control (untreated). Next, we established a model of LPS-exposed intestinal epithelial cell injury. First, the cells were exposed to LPS (Beyotime, No. ST1470, Shanghai, China) at concentrations of 0, 25, 50, 100, 200 and 400 μg/mL and cultured for 12 h, and the cell viability was determined by CCK-8 assay to confirm the exposure concentration of LPS. Then, IPEC-J2 cells were incubated at this LPS exposure concentration for 2, 4, 6, 8, and 10 h, thus the time of LPS exposure was confirmed by CCK-8 assay.

IPEC-J2 cells were pre-incubated on 6-well plates (1 × 10^6^ cells/well) for stimulation experiments. After reaching 75–80 % confluence, cells were added with 1.5 mg/mL AMC for 12 h and then exposed to 100 ug/mL LPS for 6 h. At indicated times, IPEC-J2 cells were collected for 5-ethynyl-2′-deoxyuridine (EdU) incorporation assay and cellular immunofluorescence, and the cell protein was extracted for Western Blotting.

### Statistical analysis

All basic data were analyzed with STAMP (Statistical Analysis of Metagenomic Profiles) and GraphPad Prism 9.0 (GraphPad Software, San Diego, CA, USA) software [66], [68]. Statistical analysis was performed using Student’s *t*-tests to compare differences between the two groups or one-way ANOVA followed by Tukey's post hoc pairwise comparison. The heat map was created using TBtools (V1.09854) software.

## Results

### AMC water decreased diarrhea incidence and inhibited HPA axis in MS piglets

**Fig. 1:**
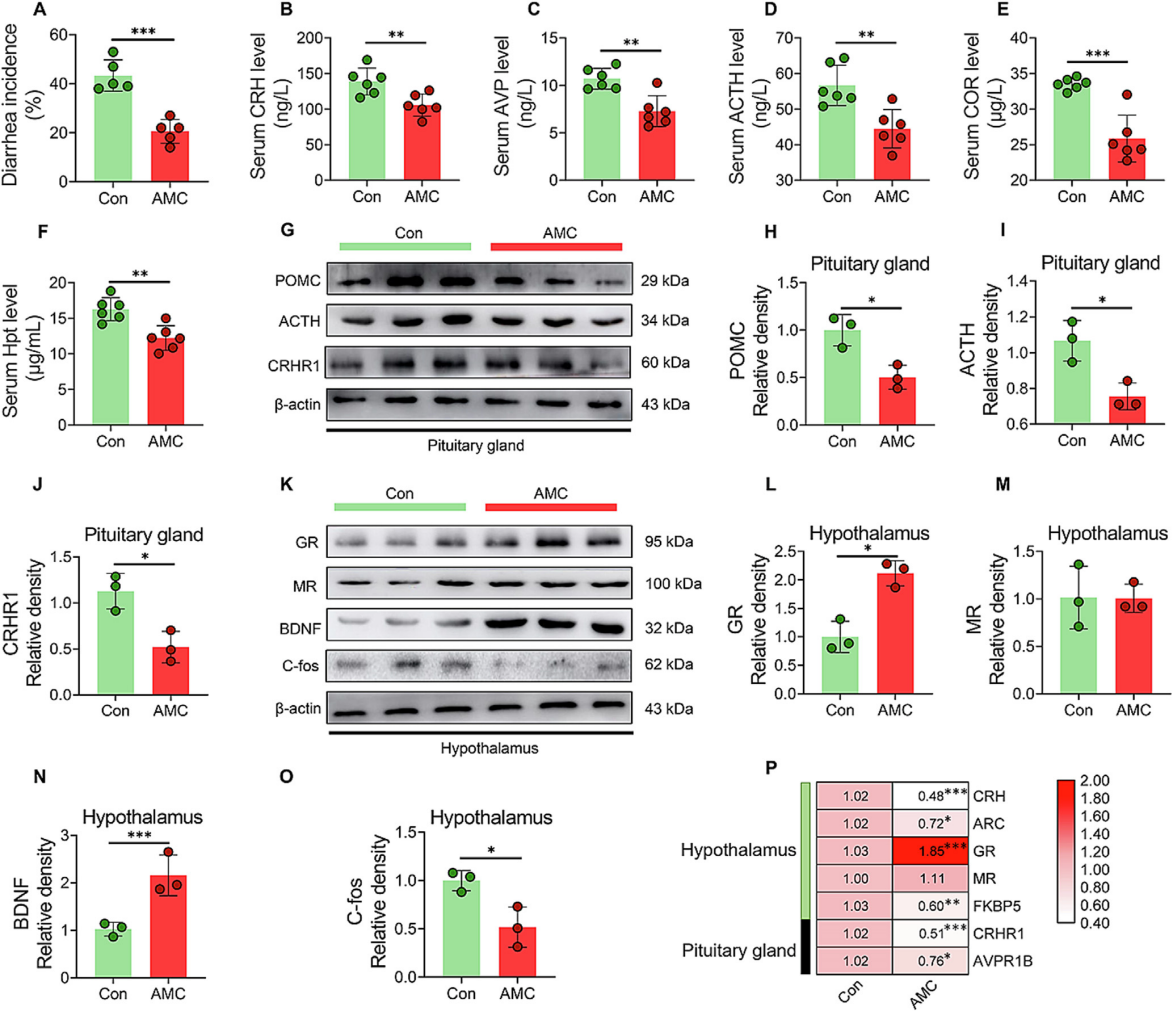
**Effect of AMC water on diarrhea incidence and hypothalamus-pituitary-adrenocortical axis in MS piglets under weaning stress.** (A) diarrhea incidence. (B-F) The serum levels of (B) CRH, (C) AVP, (D) ACTH, (E) COR and (F) Hpt. (G-J). Western blotting measurements of the protein levels of (H) POMC, (I) ACTH, and (J) CRHR1 in the pituitary gland. (K-O) Western blotting measurements of the protein levels of (L) GR, (M) MR, (N) BDNF, and (O) C-fos in the hypothalamus. (P) Heat map of mRNA levels of CRH, ARC, GR, MR, and FKBP5 in the hypothalamus, and CRHR1 and AVPR1B in the pituitary gland. Statistical analysis was performed using Student’s *t*-tests to compare differences between the two groups. Data are presented as the mean ± SD. ns, not significant, *P < 0.05, ^**^P < 0.01, and ^***^P < 0.001.

Weaning-induced stress leads to diarrhea, which increases mortality and slows growth rate in piglets [5]. The diarrhea index (Supplementary Fig. S1) and diarrhea incidence (Fig. 1A) in MS piglets under weaning stress were notably decreased (*P* < 0.001) after AMC water treatment. The HPA axis is the primary neuroendocrine stress response system of mammals [21]. As shown in Fig. 1B-F, the levels of HPA axis-related hormones CRH, arginine vasopressin (AVP), adrenocorticotrophic hormone (ACTH), cortisol (COR) and Haptoglobin (Hpt) in serum were prominently reduced in the AMC group (*P* < 0.01). In pituitary gland, AMC water markedly decreased the protein expression of ACTH (*P* < 0.05) and its precursor proopiomelanocortin (POMC), as well as CRHR1 (Fig. 1G-J). Meanwhile, at protein level, AMC water greatly enhanced GR levels (Fig. 1K-L) in the hypothalamus (*P* < 0.05), which is responsible for the negative feedback regulation of HPA axis [32]. However, there was no difference (*P >* 0.05) in MR protein levels between the two treatments (Fig. 1K, M). The protein expression of brain-derived neurotrophic factor (BDNF) and C-fos in the hypothalamus has also been proposed as indicators for identifying neural activation [33]. As presented in Fig. 1N-O, AMC water conspicuously promoted BDNF levels (*P* < 0.001), whereas reduced C-fos protein levels (*P* < 0.05) in the hypothalamus. Furthermore, the transcriptional profiles of HPA axis-related genes in the hypothalamus and pituitary were considerably altered after drinking AMC water, as reflected by decreased (*P* < 0.05) mRNA levels of CRH, ARC, FKBP5, CRHR1, and AVPR1B, and increased GR mRNA expression (*P* < 0.001, Fig. 1P). These results revealed that drinking AMC water conferred diarrhea resistance in MS piglets, which was associated with AMC-induced suppression of HPA axis.

### AMC water-induced HPA axis inhibition ameliorated gut microbiota composition in MS piglets

**Fig. 2:**
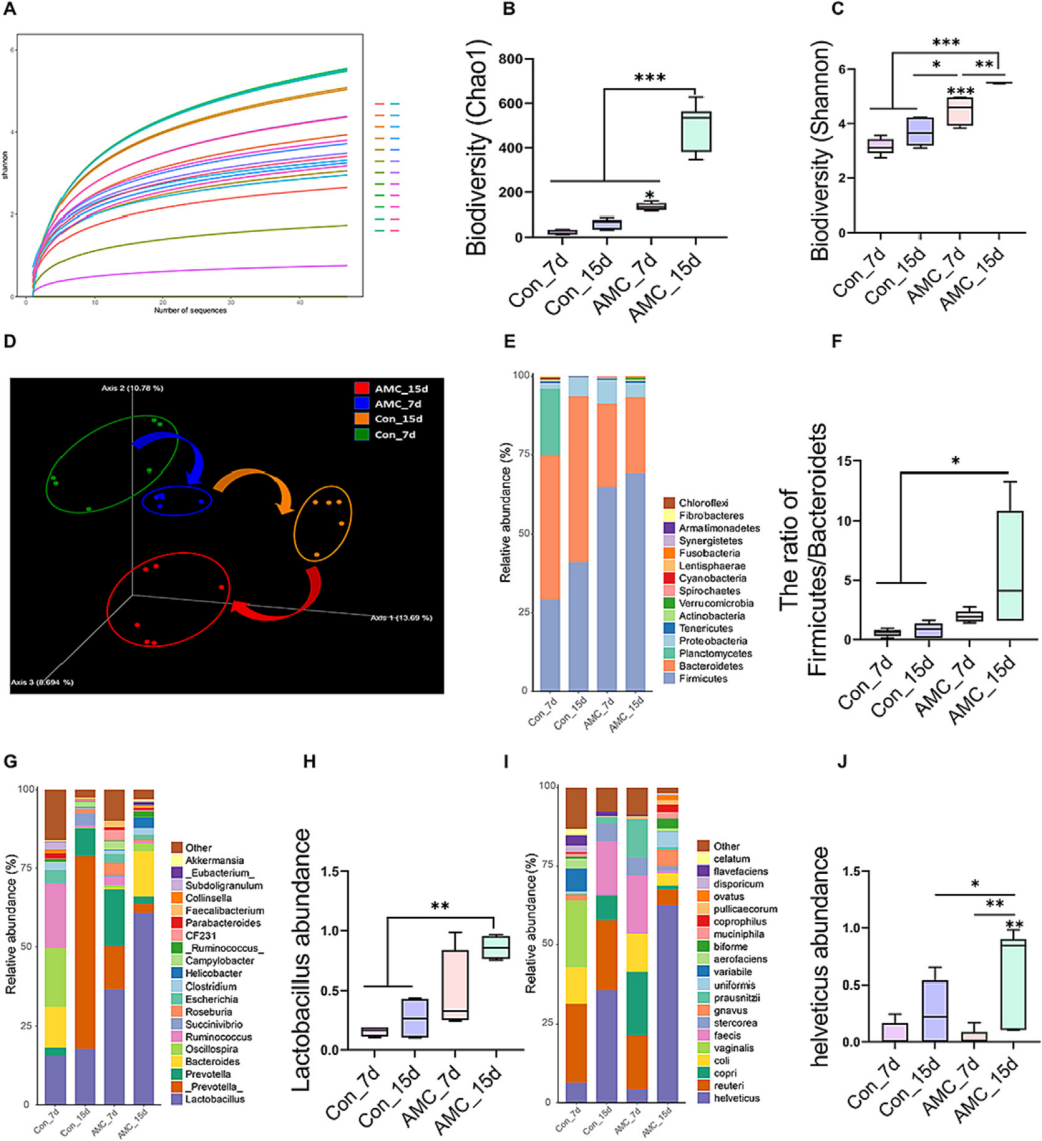
**Effect of AMC water on the diversity and composition of gut microbiota in MS piglets under weaning stress.** (A) The rarefaction curve in bacterial communities based on the Shannon index. Each sample was shown by different line colors. (B-C) Bacterial alpha diversity based on (B) Chao 1 index and (C) Shannon index (_7d/_15d, 7 days/15 days after weaning). (D) Scatterplot from Principal Coordinates Analysis (PCoA) in bacterial communities based on the weighted UniFrac distance. (E) Microbial communities bar plot at the phylum level. (F) The ratio of *Firmicutes/Bacteroidets.* (G) Microbial communities bar plot at the genus level. (H) The changes of *Lactobacillus* abundance. (I) Microbial communities bar plot at the species level. (J) The changes of *helveticus* abundance. Statistical analysis was performed using one-way ANOVA followed by Tukey's post hoc pairwise comparison. Data are presented as the mean ± SD. ns, not significant, *P < 0.05, ^**^P < 0.01, and ^***^P < 0.001.

Gut microbiota is involved in intestinal injury repair in MS piglets under weaning stress, and it interacts with the brain via the HPA axis [21]. We investigated the gut microbial alterations associated with repaired gut injury using 16S-rRNA PacBio SMRT Gene Full-Length Sequencing analyses in piglets at two time points (Con/AMC_7d and Con/AMC_15d) after separation. Rarefaction curves showed that nearly all bacterial species were recognized in stool samples (Fig. 2A). The alpha diversity markers of gut microbes, (Fig. 2B) Chao1 and (Fig. 2C) Shannon indices, were conspicuously elevated on (*P* < 0.05) day 7 and (*P* < 0.001) day 15 of AMC treatment group. Meanwhile, AMC caused the transfer of Con_7d to AMC_7d to Con_15d to AMC_15d in MS piglets intestinal microbial beta diversity, as presented in the scatterplot from PCoA (Fig. 2D). The PCA of the Kyoto Encyclopedia of Genes and Genomes (KEGG) pathway (Supplementary Fig. S2) also showed that AMC induced shifts from Con_7d to Con_15d to AMC_7d to AMC_15d in the functional profiles of piglet intestinal bacterial communities. Furthermore, microbial taxonomic analyses (Fig. 2E-F) exhibited that the ratio of *Firmicutes*/*Bacteroidets* (*P* < 0.05) was apparently higher in AMC_15d MS piglets compared to the Con group (on days 7 and 15). At the genus level (Fig. 2G-H), AMC treatment notably increased *Lactobacillus* abundance at both time points (*P* < 0.01). Likewise, at the species level (Fig. 2I-J), *helveticus* abundance was significantly increased in MS piglets of AMC_15d group (*P* < 0.05), compared to the other treatments. These results indicated that AMC-induced HPA axis suppression could improve the diversity of gut microflora and ameliorate its composition to remodel the gut microbiota of MS piglets.

### AMC water-induced HPA axis inhibition modulated the marker bacterial taxa, core bacteria interaction and ameliorated functional profiles of gut microbe

**Fig. 3:**
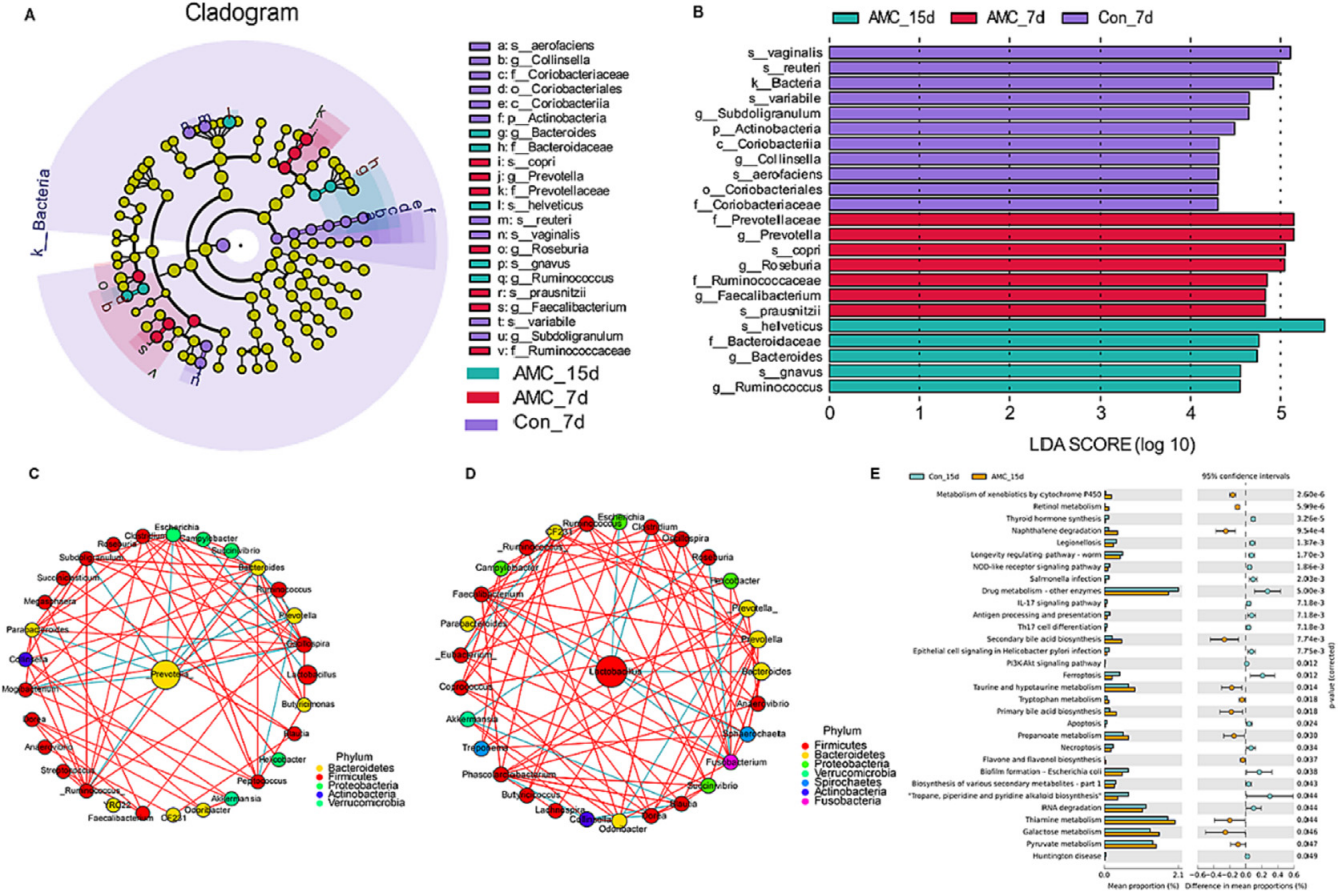
**Effect of AMC water on marker bacterial, microbiota interaction network and functional profiles in MS piglet.** (A) Linear discriminant analysis effect size (LEFse) analysis on gut microbiota. Linear discriminant analysis (LDA): an LDA score higher than 2 indicated a higher relative abundance in the corresponding group than in other groups. (B) LEfSe taxonomy cladogram: different colors suggest enrichment of certain taxa in AMC_15d (green), AMC_7d (red) and Con_7d (purple). The size of the circles is based on relative abundance. The significantly different species using the nonparametric factorial Kruskal–Wallis rank sum test at a significance level of 0.05. (C-D) Microbiota interaction network analysis of common core fecal bacteria in the (C) Con group and (D) AMC group. The red line means a positive correlation, and the green means negative correlation. (E) The comparative analysis for the relative abundances of bacterial KEGG pathways between the Con group and AMC group. *P-value* < 0.05 means a significant difference. (For interpretation of the references to colour in this figure legend, the reader is referred to the web version of this article.)

LEfSe analysis recognized 22 marker bacterial taxa that were radically different, with an LDA score>2, indicating that there was an apparently structural difference among the treatments, in which 8 bacterial species were confirmed as key discriminants (*P* < 0.05, Fig. 3A-B). The species marker microflora of Con_7d piglets included *vaginalis*, *reuteri*, *variabile*, and *aerofaciens*, the AMC_7d piglets included *copri* and *prausnitzii*, and the AMC_15d piglets included *helveticus* and *gnavus*, whereas no key discriminants of Con_15d piglets were observed. To further research the effect of AMC water on microflora composition at the genus level, we used the Spearman correlation matrix to perform an interaction correlation network analysis. Compared to the Con group (Fig. 3C), AMC water changed the abundance of several core bacteria, resulting in stronger, clearer, and more specific interactions (Fig. 3D). It was worth emphasizing that, a potential pathogen, *Prevotella*, was the central genus in the microbiota interaction network in the Con group, whereas the central genus in AMC group was *Lactobacillus*, a widely accepted probiotic genus. These findings suggested that drinking AMC water may induce the shift of the gut core microflora from neutral or pathogenic to beneficial microflora in MS piglets under weaning stress.

As shown in Fig. 3E, compared to the Con_15d treatment, AMC_15d treatment conspicuously increased (*P* < 0.05) the abundance of genes responsible for biologically beneficial metabolic and synthetic pathways, including the metabolism of retinol, tryptophan, taurine, hypotaurine, pyruvate, thiamine and galactose, together with the biosynthesis of primary and secondary bile acid, flavone and flavonol. Meanwhile, the abundance of genes related to cell death, such as apoptosis, ferroptosis, and necroptosis, were markedly reduced after treated with AMC water. Moreover, drinking AMC water remarkably suppressed the abundance of genes involved in disease infection, including epithelial cell signaling in helicobacter pylori infection, legionellosis, salmonella infection, and huntington disease. Importantly, the relative gene abundances of inflammatory-related pathways, NOD-like receptor, PI3K-Akt and IL-17 signaling pathways, were markedly decreased with supplementation of AMC water. Thus, all of these findings showed that drinking AMC water can improve the composition of intestinal microflora in MS piglets, modulate the core microbiota interaction network, and ameliorate the functional profiles. Could changes in gut microbiota be related to reduced activity of the HPA axis? This is a query.

### LPS-induced inflammatory factors and stress hormones released by the HPA axis mediated bidirectional communication in the brain-microbe axis

**Fig. 4:**
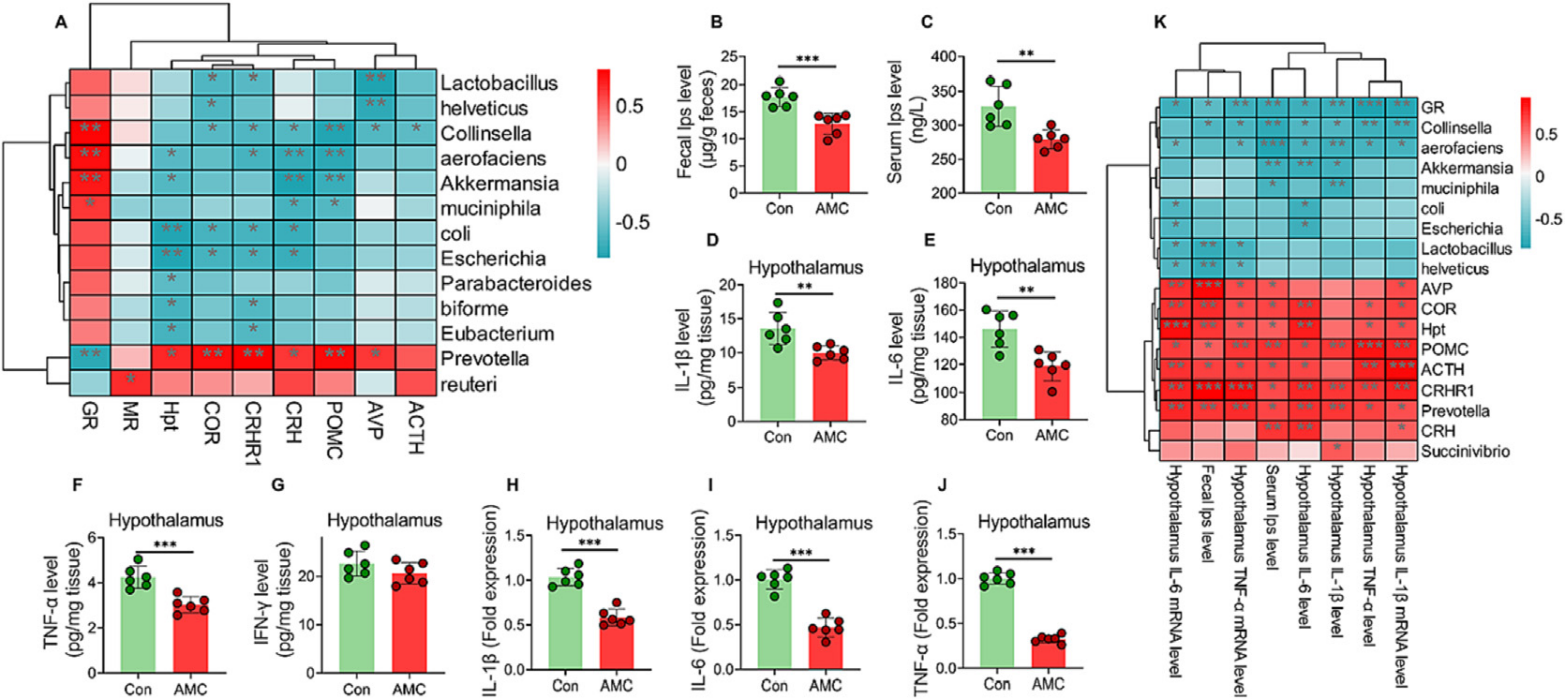
**Effects of AMC water on interaction mediators of the brain-microbe axis in MS piglets under weaning.** (A) Correlation analysis between HPA axis and gut microbes. (B) Fecal LPS levels. (C) Serum LPS levels. (D-G) The levels of proinflammatory factors (D) IL-1β, (E) IL-6, (F) TNF-α and (G) IFN-γ in the hypothalamus. (H-J) The mRNA fold expression of (H) IL-1β, (I) IL-6 and (J) TNF-α in the hypothalamus. (K) Correlation analysis between fecal/serum LPS, hypothalamus proinflammatory factors and brain-microbes axis. Statistical analysis was performed using Student’s *t*-tests to compare differences between the two groups. Data are presented as the mean ± SD. ns, not significant, *P < 0.05, ^**^P < 0.01, and ^***^P < 0.001.

A concept has been recognized: the nervous system regulates the immune system through the interplay of cytokines and hormones in various organs [22]. As shown in Fig. 4A, there was an obvious negative correlation between the stress hormones and the intestinal beneficial bacteria, such as the COR, AVP and the *Lactobacillus* and *Helveticus* (*P* < 0.05). Notably, the HPA axis negative feedback regulator, GR, was positively correlated with beneficial bacteria, like *Akkermansia* (*P* < 0.01)*,* but negatively related to the potential pathogen *Prevotella* (*P* < 0.01). These results validated the hypothesis that supression of HPA axis by AMC water improved the gut microbiota composition in MS piglets. It is well known that translocation of LPS into the systemic circulation is a consequence of increased intestinal permeability. As shown in Fig. 4B-C, the levels of the gut microbiota metabolite LPS were significantly reduced in both (*P* < 0.001) feces and (*P* < 0.01) serum in MS piglets treated with AMC water. The translocation of LPS into the systemic circulation was suppressed by AMC-induced intestinal barrier function promotion, which was manifested by lower levels of proinflammatory factors (*P* < 0.001) IL-1β, (*P* < 0.001) IL-6, and (*P* < 0.01) TNF-α in the hypothalamus (Fig. 4D-J). Importantly, correlation analysis exhibited that the contents of LPS and the levels of inflammatory factors in the hypothalamus were positively correlated with the HPA axis-related hormones, whereas they were negatively correlated with the gut-beneficial bacteria (*P* < 0.05, Fig. 4K). Therefore, it can be confirmed that the gut microbiota metabolite LPS and its-activated proinflammatory factors mediated the bidirectional interaction between the brain and the gut via the HPA axis.

### AMC water-induced microbiota improvement attenuated weaning-caused gut morphological injury in MS piglets

**Fig. 5:**
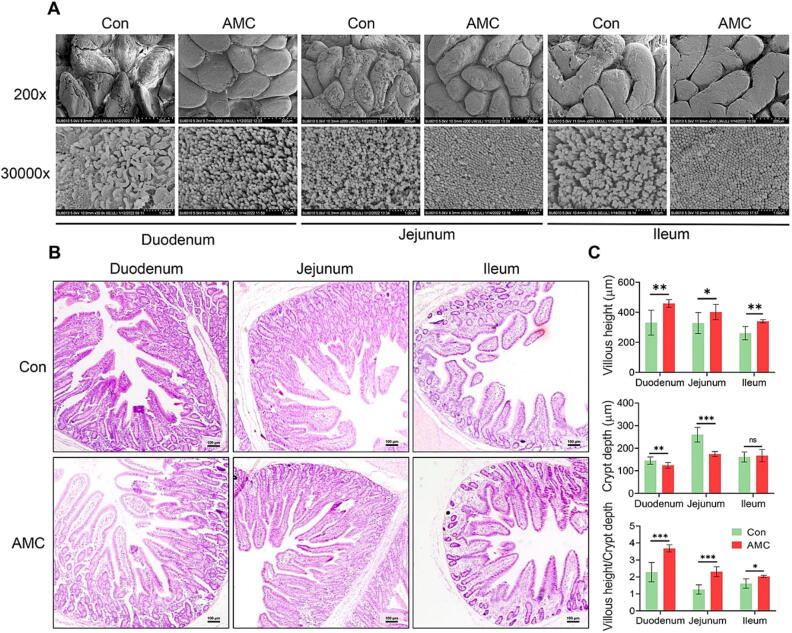
**Effect of AMC water on intestinal morphology in MS piglets under weaning stress.** (A) Intestinal morphology shown by SEM (at 200x and 30000x magnification) and (B**)** H&E staining (scale bar 100 μm) of duodenum, jejunum, and ileum tissues in MS piglets. Fields from one representative experiment of three are shown (n = 3). (C) Statistical analysis of villus height (µm), crypt depth and villous height/crypt depth of MS piglets. For each group, a minimum of 8 villi from each sample were determined by Image J software (n = 8). Statistical analysis was performed using Student’s *t*-tests to compare differences between the two groups. Data are presented as the mean ± SD. ns, not significant, *P < 0.05, ^**^P < 0.01, and ^***^P < 0.001.

H&E staining and SEM were applied to assess the effect of AMC water on morphological damage of small gut epithelium in MS piglets under weaning stress. SEM results (200x, Fig. 5A, upper) revealed that weaning stress induced obviously surface epithelium damage to villi in small intestine, which was relieved by AMC water treatment. More importantly, AMC water elevated the micro-villi number in the small gut, exhibiting as a more tidy and dense micro-villi morphology (Fig. 5A, nether, at 30000x magnification). H&E staining also supported these changes. As presented in Fig. 5B-C, AMC water markedly elevated villous height (VH) and the ratio of VH: crypt depth (VH:CD) in the duodenum (*P* < 0.01, *P* < 0.001), jejunum (*P* < 0.05, *P* < 0.001), and ileum (*P* < 0.01, *P* < 0.05). Meanwhile, the CD was clearly reduced in the duodenum (*P* < 0.05) and jejunum (*P* < 0.01) after AMC water treatment. Therefore, drinking AMC water could ameliorate gut pathological injury induced by weaning in MS piglets, but is this related to gut microbe improvement?

### AMC water enhanced goblet cell and Paneth cell numbers in the small intestine

**Fig. 6:**
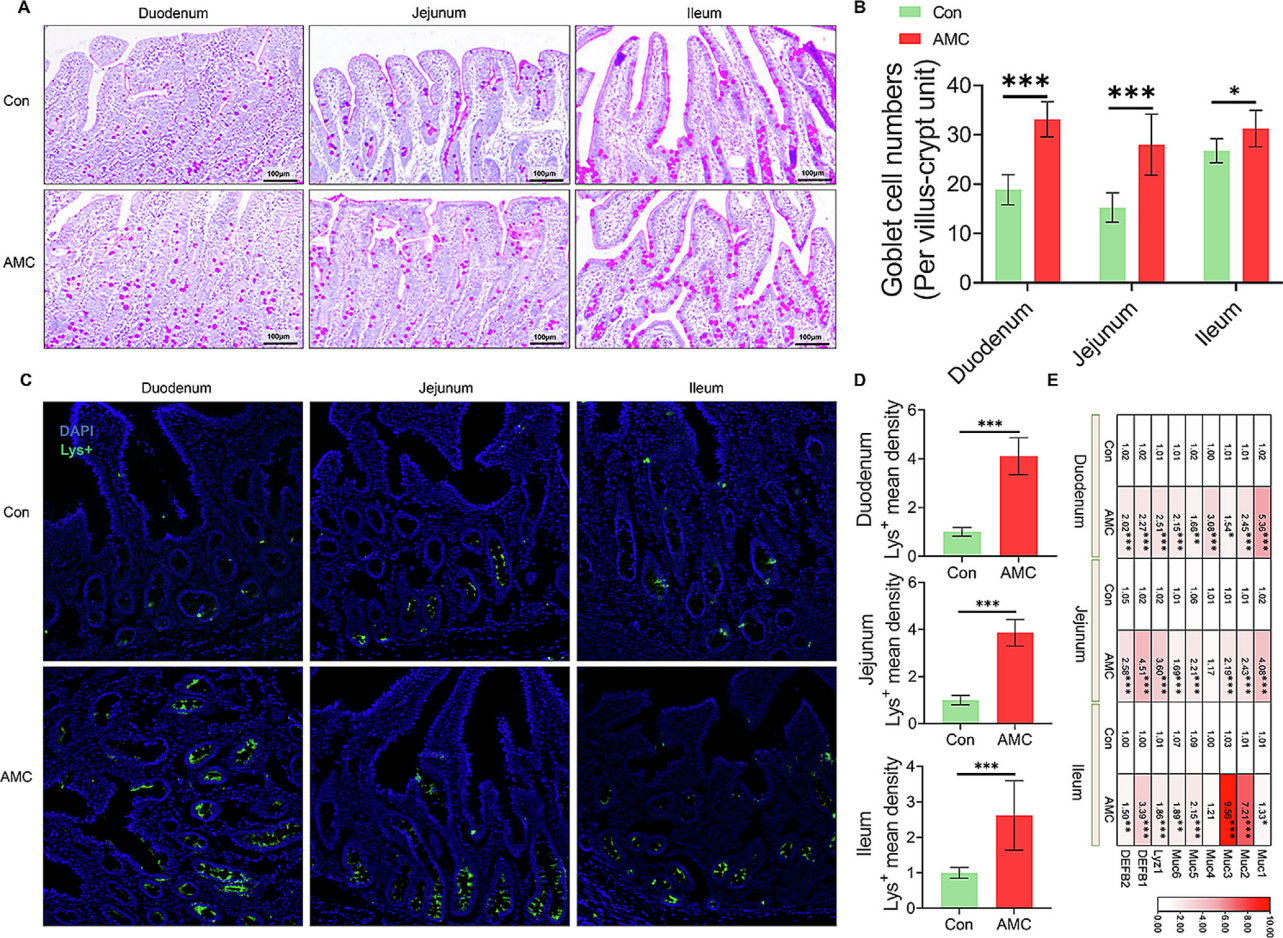
**Effect of AMC water on the number of goblet cells and Paneth cells in the small intestine of MS piglets.** (A) The intestinal goblet cells shown by PAS staining of duodenum, jejunum, and ileum tissues in MS piglets (scale bar 100 μm). (B) Statistical analysis of the goblet cells number in the duodenum, jejunum, and ileum (n = 3). (C) The intestinal Paneth cells marked by Lys + of duodenum, jejunum, and ileum tissues in MS piglets. The mean optical density was analyzed by Image J software. (D) Statistical analysis of Lys + mean density in the duodenum, jejunum, and ileum (n = 3). (E) Heat map of mRNA levels of Mucins (Muc 1–6), lysozyme (Lyz1), and defensins (DEFB1-2) in duodenum, jejunum, and ileum tissues. The mRNA levels were determined by qRT-PCR (n = 6). Statistical analysis was performed using Student’s *t*-tests to compare differences between the two groups. Data are presented as the mean ± SD. ns, not significant, *P < 0.05, ^**^P < 0.01, and ^***^P < 0.001.

Goblet cells and Paneth cells, which differentiate from crypt multipotent adult ISC, exert a vital role in sustaining gut mucosal immunity [26]. As presented in Fig. 6A-D, AMC water markedly increased the number of goblet cells and Paneth cells (Lys + ) in the (*P* < 0.001) duodenum, (*P* < 0.001) jejunum and (*P* < 0.05, *P* < 0.001) ileum. The primary function of goblet cells is to produce and secrete mucins (Muc), establish a mucosal barrier, protect ISC, and limit LPS translocation [34]. Compared to the Con group, the mRNA levels of Muc1, Muc2, Muc3, Muc5, and Muc6 were significantly enhanced (*P* < 0.05) in the AMC group in all small intestine segments (Fig. 6E). Paneth cells exert a cardinal effect on innate immunity due to their ability to produce lysozyme, antibiotics, and defensins [35]. Likewise, AMC water significantly elevated the transcriptional levels of Lyz1, DEFB1, and DEFB2 in the small intestine (*P* < 0.001). These results uncovered that drinking AMC water could boost Muc and defensins expression by increasing the number of goblet cells and Paneth cells, thereby preventing gut injury in MS piglets.

### AMC water activated Wnt/β-catenin signaling pathway to drive ISC differentiation

**Fig. 7:**
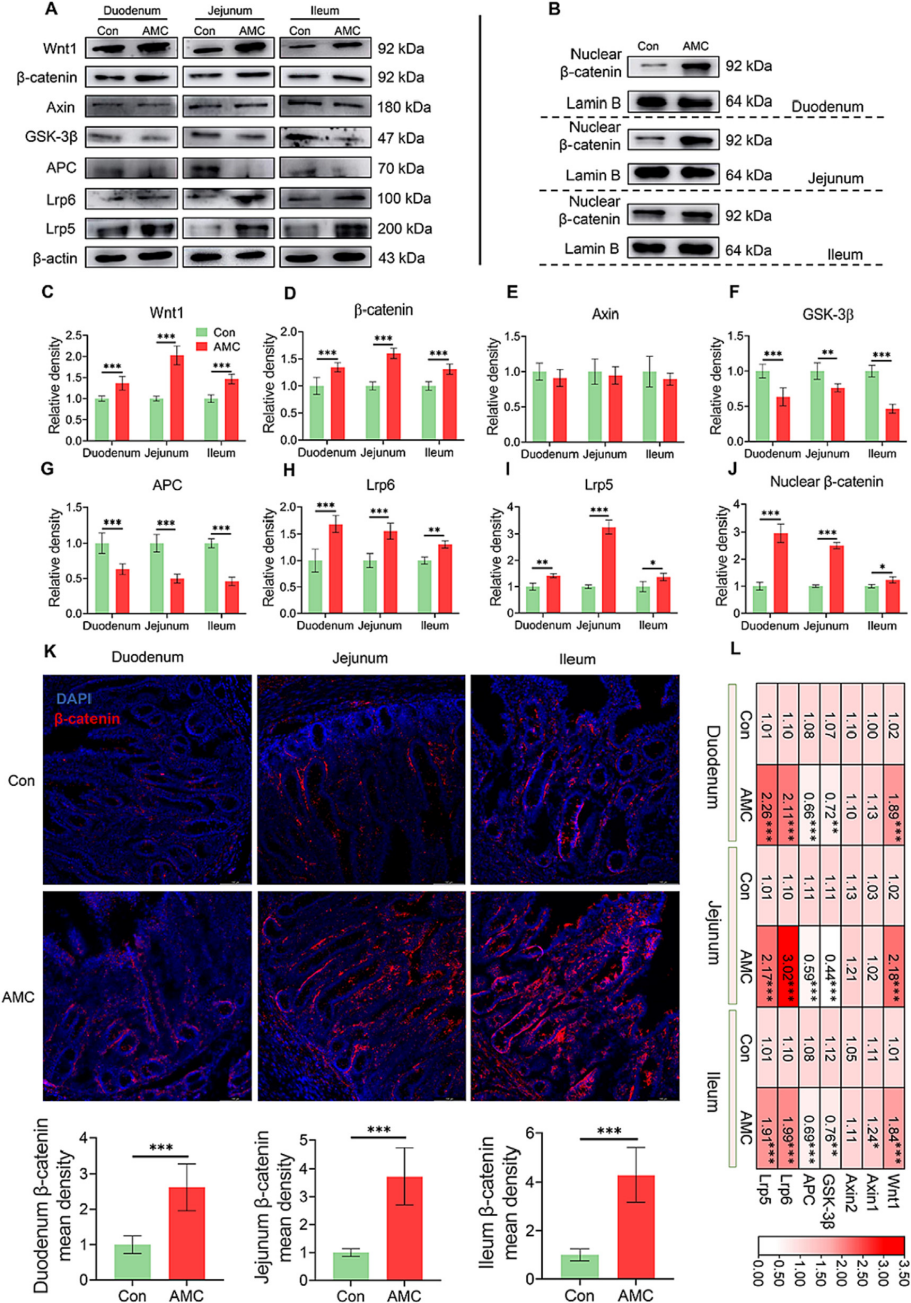
**Effect of AMC water on Wnt/β-catenin signaling pathway of small intestine in MS piglets.** (A) Western blotting measurements of the protein levels of Wnt/β-catenin signaling pathway in small intestine (including duodenum, jejunum, and ileum) from MS piglets. (B) Western blotting measurement of the protein levels of nuclear β-catenin of duodenum, jejunum, and ileum in MS piglets. (C-J) The protein levels of (C) Wnt1, (D) total β-catenin, (E) Axin, (F) GSK-3β, (G) APC, (H) Lrp6, (I) Lrp5, and (J) nuclear β-catenin of duodenum, jejunum, and ileum in weaned-piglets. (K) Immunofluorescence images of β-catenin staining (red) and DAPI (blue) of duodenum, jejunum, and ileum in MS piglets. The mean density of β-catenin was detected. (L) Heat map of mRNA levels in Wnt/β-catenin signaling pathway. Statistical analysis was performed using Student’s *t*-tests to compare differences between the two groups. Data are presented as the mean ± SD. ns, not significant, *P < 0.05, ^**^P < 0.01, and ^***^P < 0.001. (For interpretation of the references to colour in this figure legend, the reader is referred to the web version of this article.)

Wnt/β-catenin signaling is the main driver of crypt ISC differentiation and proliferation [36]. Thus, we speculated that the elevated number of goblet cells and Paneth cells was caused by Wnt/β-catenin signaling pathway activation. As shown in Fig. 7A, AMC water dramatically enhanced (*P* < 0.001) the protein expression of Wnt1 (Fig. 7C) and β-catenin (Fig. 7D) in the duodenum, jejunum and ileum of MS piglets. Of note, the increases in the fluorescence density (Fig. 7K) and nuclear protein levels (Fig. 7B, J) of β-catenin further confirmed this result. A degradation complex consisting of Axin, APC, GSK-3β, and casein kinase I targets β-catenin for proteasomal degradation by phosphorylation. The protein levels of GSK-3β (*P* < 0.01, Fig. 7F) and APC (*P* < 0.001, Fig. 7G) were dramatically decreased in the small intestine when subjected to AMC water, but no change was recorded in Axin (Fig. 7E) between the two groups (*P >* 0.05). On the other hand, the Wnt ligand binds to LRP5/6, inactivating the complex in an unknown manner [37]. In this study, the Lrp6 (Fig. 7H) and Lrp5 (Fig. 7I) protein levels of AMC group were notably elevated (*P* < 0.05) in the small intestine. Additionally, at the transcriptional level, the results of mRNA expression of these factors were in agreement with the changes in protein expression (Fig. 7L). These findings revealed that the stimulation of the Wnt/β-catenin signaling pathway was responsible for the increase in the number of goblet cells and Paneth cells in the small gut.

### AMC water drove ISC proliferation via the Wnt/β-catenin signaling pathway in MS piglets

**Fig. 8:**
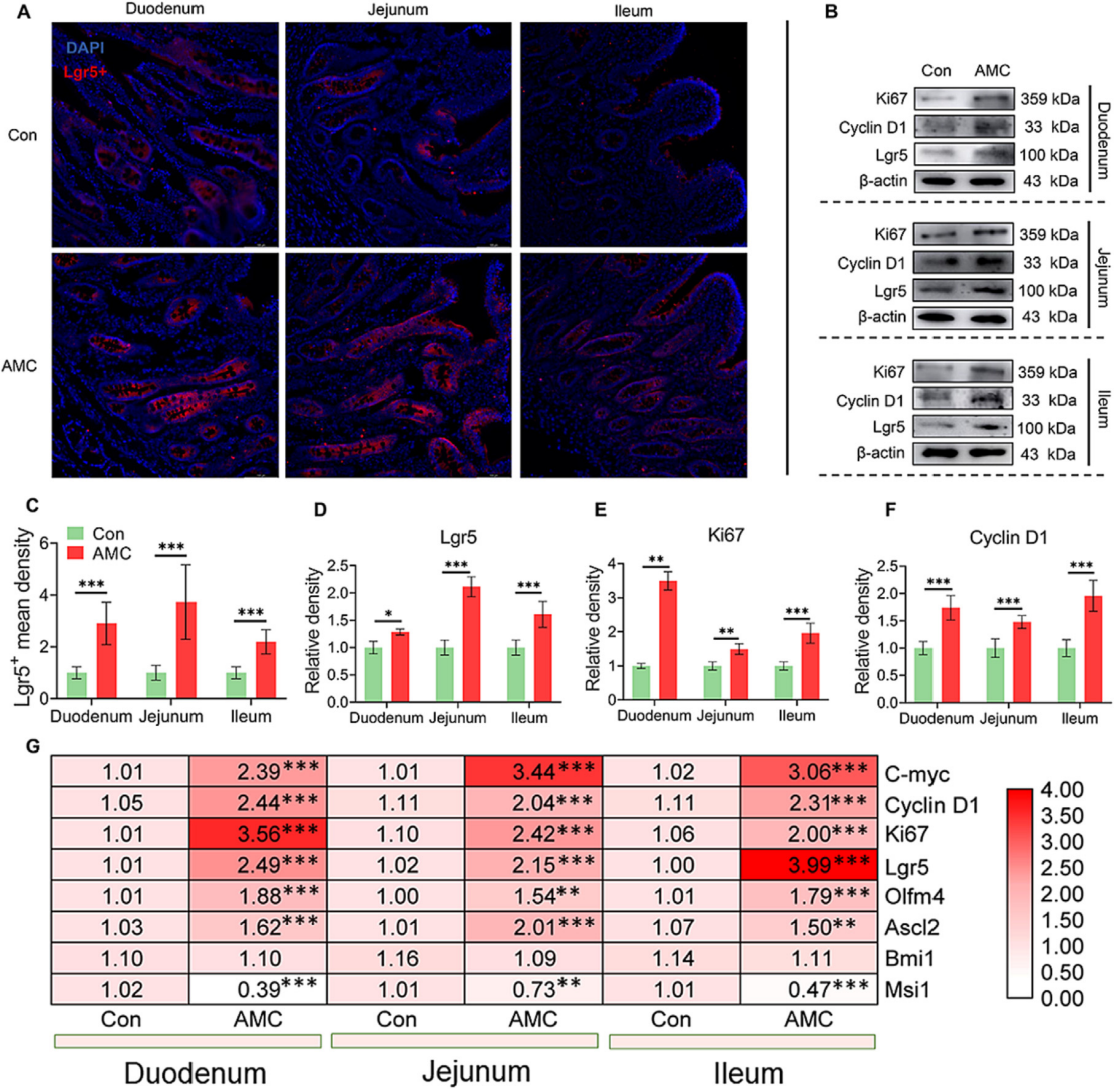
**Effect of AMC water on intestinal stem cells proliferation of small intestine in MS piglets.** (A) Immunofluorescence images of Lgr5 + staining (red) and DAPI (blue) of duodenum, jejunum, and ileum in MS piglets. (B) Western blotting measurements of protein levels of cell proliferation markers of duodenum, jejunum, and ileum in MS piglets. (C) Mean density of Lgr5 + in duodenum, jejunum, and ileum of MS piglets. (D-F) The protein levels of (D) Lgr5, (E) Ki67, and (F) Cyclin D1 of duodenum, jejunum, and ileum in MS piglets. (G) Heat map of mRNA levels of cell proliferation markers (C-myc, CyclinD1, Ki67), active ISC markers (Lgr5, Olfm4, Ascl2), and quiescent ISC marker (Bmi1) in duodenum, jejunum, and ileum of MS piglets. Statistical analysis was performed using Student’s *t*-tests to compare differences between the two groups. Data are presented as the mean ± SD. ns, not significant, *P < 0.05, ^**^P < 0.01, and ^***^P < 0.001. (For interpretation of the references to colour in this figure legend, the reader is referred to the web version of this article.)

To further investigate the role of Wnt/β-catenin signaling pathway activation in intestinal epithelial proliferation, we evaluated the levels of cell proliferation-related factors and ISC markers. Maintenance of self-renewal of Lgr5 + ISC in crypts is critical to support intestinal homeostasis [38]. AMC water markedly promoted the Lgr5+ (Fig. 8A, C) ISC density (*P* < 0.001) and Lgr 5 (Fig. 8B, D) protein levels (*P* < 0.05) in all small intestine segments. In addition, AMC treatment fueled intestinal epithelial proliferation with noticeably increased protein or mRNA levels of Ki67 (*P* < 0.01, Fig. 8B, E, G), Cyclin D1 (*P* < 0.001, Fig. 8B, F, G) and C-myc (*P* < 0.001, Fig. 8G). Meanwhile, the active ISC markers (Lgr5, Olfm4, Ascl2) mRNA levels were increased in the small intestine (*P* < 0.05) of MS piglets treated with AMC water (Fig. 8G). By contrast, the mRNA level of Bmi1 (a quiescent ISC marker) was significantly decreased after AMC water treatment (Fig. 8G). These results validated that AMC water activated Wnt/β-catenin signaling pathway to drive the ISC proliferation, consequently repairing the damaged intestinal epithelium.

### AMC treatment in the absence of microbes did not promote ISC differentiation into goblet cells and Paneth cells

**Fig. 9:**
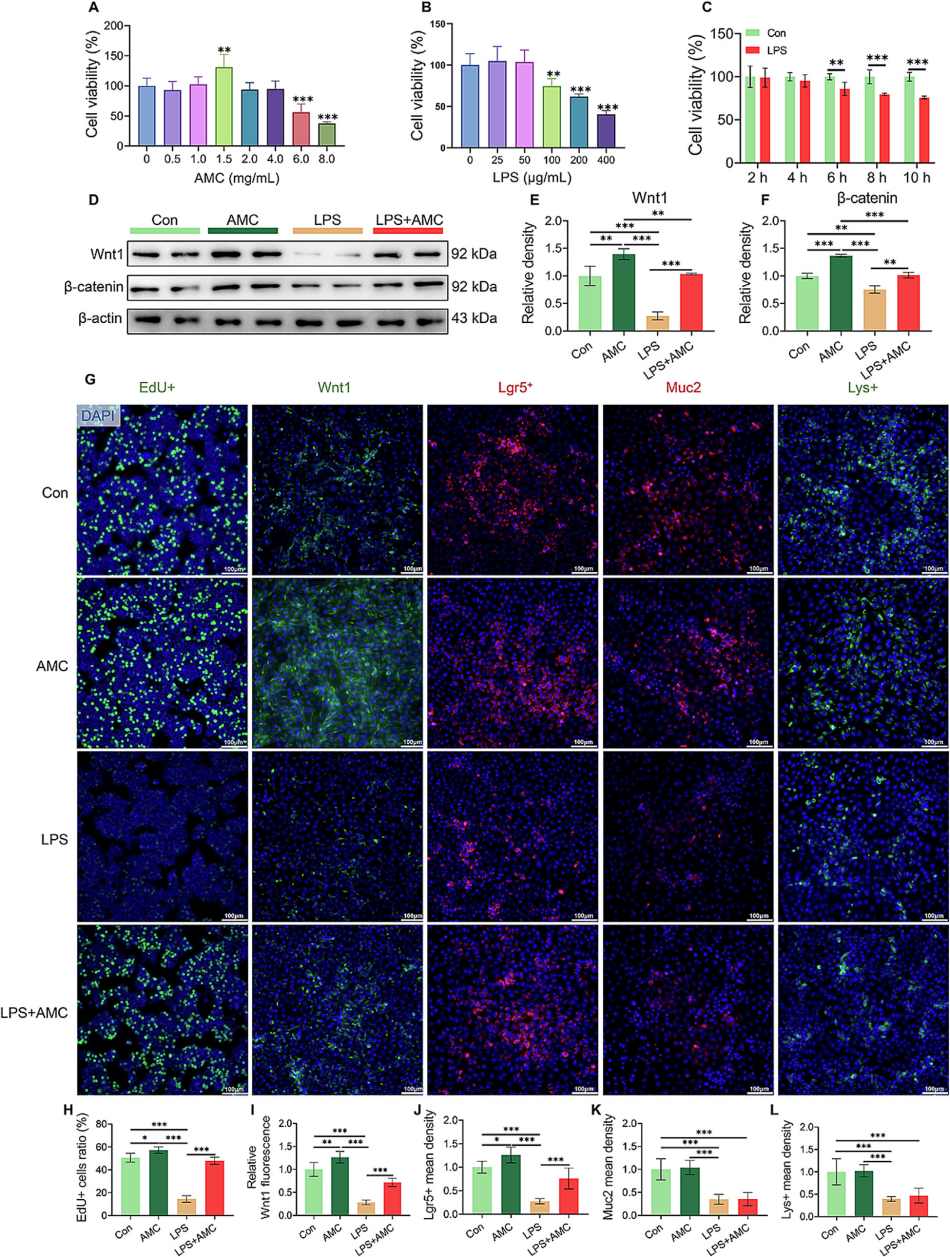
**Effect of AMC on IPEC-J2 cells proliferation and differentiation in vitro.** (A) Cell viability in various concentrations of AMC (0, 0.5, 1.0, 1.5, 2.0, 4.0, 6.0 and 8.0 mg/mL) treatment. (B) Cell viability in various concentrations of LPS (0, 25, 50, 100, 200 and 400 μg/mL) exposure. (C) Cell viability at 2, 4, 6, 8 and 10 h after 100 μg/mL LPS exposure. (D-F) Western blotting measurements of the protein levels of (E) Wnt1 and (F) β-catenin in the IPEC-J2 cells. (G-L) Immunofluorescence images of (H) EdU+ (green), (I) Wnt1 (green), (J) Lgr5+ (red), (K) Muc2 (red), (L) Lys+ (green) and DAPI (blue) staining in IPEC-J2 cells. The mean optical density or cell number was analyzed by Image J software. Statistical analysis was performed using Student’s *t*-tests to compare differences between the two groups or one-way ANOVA followed by Tukey's post hoc pairwise comparison. Data are presented as the mean ± SD. ns, not significant, *P < 0.05, ^**^P < 0.01, and ^***^P < 0.001. (For interpretation of the references to colour in this figure legend, the reader is referred to the web version of this article.)

To investigate the involvement of gut microbiota in AMC-induced gut epithelial regeneration, we performed an in vitro evaluation (in the absence of microbes) using IPEC-J2 cell line. In physiological conditions, AMC promoted the proliferation of IPEC-J2 cells, and the best treatment concentration is 1.5 mg/mL (*P* < 0.01, Fig. 9A). By CCK-8 viability assays, we established a model of LPS-induced IPEC-J2 injury and a model of AMC antagonism: after pretreatment with 1.5 mg/mL AMC concentrate for 12 h, IPEC-J2 cells were exposed to LPS at 100 ug/mL for 6 h (Fig. 9B-C). As shown in Fig. 9D-F, the protein levels of Wnt1 and β-catenin were significantly increased in AMC treatment group, which proved that AMC could improve the proliferation of IPEC-J2 under physiological conditions by activating the Wnt/β-catenin pathway. Importantly, AMC treatment significantly reversed LPS-induced suppression of Wnt/β-catenin signaling pathway (*P* < 0.01), which was also verified with enhanced Wnt1 fluorescence (*P* < 0.001, Fig. G, I) in cells. Meanwhile, the results of EdU + cell ratio (*P* < 0.001, Fig. 9G, H) and Lgr5 + mean density (*P* < 0.001, Fig. 9G, J) also indicated that AMC drove the proliferation of LPS-exposed IPEC-J2 cells by activating the Wnt/β-catenin pathway. Interestingly, AMC treatment did not restore (*P >* 0.05) the LPS-suppressed expression of the goblet cell marker Muc2 (Fig. 9G, K) and the Paneth cell marker Lys+ (Fig. 9G, L). These results suggested that, AMC treatment can promote the proliferation of IEC by activating the Wnt/β-catenin signaling; but it cannot promote LPS-suppressed cell differentiation in vitro without the intervention of intestinal microbes.

## Discussion

Weaning leads to weight loss, nutrient waste, immune homeostasis disruption, and pro-inflammatory response activation, resulting in susceptibility to potential pathogens and diarrhea in young mammals [39], [40], [41]. It has long been known that antibiotics are effective in treating post-weaning diarrhea in mammals. Nonetheless, widespread antibiotic use causes superbug occurrences (by increasing bacterial resistance), public health concerns, and food safety dangers to humans [42], [43]. In the present study, we found that drinking AMC water notably reduced diarrhea incidence in MS piglets under weaning stress, indicating that AMC water may be a promising antibiotic alternative for preventing diarrhea. The benefits of alkaline mineral water have revealed that those who drank water with alkaline minerals had decreased rates of coronary heart disease, cardiovascular disease, cancer incidence, and overall mortality. Meanwhile, the intestinal beneficial effects of AMC water have been widely reported, including ameliorating intestinal disturbances, promoting gastrointestinal motility, alleviating gastrointestinal injury, and treating IBS-diarrhea [3]. Our study demonstrates that drinking AMC water confers diarrhea resistance via the brain-microbe-gut axis in MS piglets, furthering the understanding of the interplay of neurological, microbial and gut immunity in gastrointestinal tract disorder therapy.

Stress is one of the primary causes of diarrhea in young mammals, whether direct or indirect. The activation of the HPA axis and the sympathetic nervous system is the body's adaptive reaction to stress, resulting in the enormous release of stress indicators such as COR and Hpt [33]. Excessive activation of these mechanisms may trigger systemic biological responses with deleterious effects [44]. Evidence has consistently demonstrated that weaning stress exposure causes disturbances of the HPA axis in MS piglets and induces a variety of biological abnormalities, including subclinical inflammation, low levels of BDNF, microbiota dysbiosis, and increased intestinal permeability [45]. Our study demonstrated that drinking AMC water reduced HPA axis and serum levels of stress hormones COR and Hpt, whereas increasing BDNF expression in the brain, suggesting the anti-stress properties of AMC water. It has been reported that the stress hormones induced by HPA axis activation can influence gut microbiota composition and gut permeability [21]. The gut microbiota can certainly affect the activity of the HPA axis in the brain via mediators that can penetrate the blood–brain barrier, including microbial antigens and cytokines [22]. Clearly, the brain and gut interact bidirectionally through the HPA axis and gut microbiota mechanisms. In the present study, our results found that AMC can act as a microbiota regulator, which can increase the diversity of gut microbiota and improve its composition, interaction network, and functional profile. It is worth noting that the results of PCoA from beta diversity and PCA from KEGG pathway indicated that the microflora composition and function of piglets showed a clockwise change from Con_7d to AMC_7d to Con_15d to AMC_15d and Con_7d/15d to AMC_7d/15d, respectively, suggesting that the composition and diversity changes of microbiota induced by AMC water may cause the microbiota of MS piglets to reach the homeostasis period earlier than that of the Con group. This finding is supported by the *Firmicutes* to *Bacteroidets* ratio, a lower ratio of which is thought to be a precursor to dysbiosis [46]. In addition, LEfSe analysis showed that the gut beneficial bacteria *Lactobacillus helveticus* (*L.helveticus*) and *Ruminococcus gnavus* (*R.gnavus*) were the marker microbes that conferred diarrhea resistance in MS piglets after 15 days of drinking AMC water. A rat study revealed that *L.helveticus* attenuated d-galactosamine-induced liver damage by regulating the gut microflora and metabolome, suggesting this species has the potential to repair organ damage [47]. Likewise, as a mucus-degrading gut bacterium, *R. gnavus*, promotes early gut microbiota colonization in young animal intestine by an endogenous nutrient supply manner [48]. Therefore, drinking AMC water improved gut microbiota diversity and community maturation in MS piglets, which may be related to *R. gnavus* promoted microbe colonization.

Gut microflora and the host have undergone long-term co-evolution, participating in and affecting the physiological function and metabolic spectrum of the host. In this study, we performed a functional predictive analysis of the identified differential communities. Our results indicated that the gene abundance of some biologically beneficial metabolic and synthetic pathways was significantly increased after AMC water treatment, such as the metabolism of retinol, taurine, pyruvate, and thiamine, as well as the biosynthesis of primary and secondary bile acids. Reportedly, retinol [49], taurine [50], and pyruvate [51] regulate intestinal mucosal immune homeostasis and intestinal barrier integrity through different pathways, which play an important role in maintaining intestinal homeostasis. In addition, bile acids can regulate gut microbial composition both directly and indirectly by activating innate immune response genes in the small intestine [52]. These findings revealed that AMC water improved intestinal metabolism and the synthesis of beneficial molecules in MS piglets by regulating intestinal microflora function. Likewise, it is worthy of highlighting that AMC water reduced the risk of host intestinal diseases, including helicobacter pylori and salmonella infection, which have been shown to cause diarrhea and intestinal barrier damage in piglets [53]. Moreover, the improvement of microflora structure may also alleviate intestinal epithelial cell death and intestinal inflammation, as reflected by the down-regulated abundance of inflammation-related pathways (NOD-like receptor, PI3K-Akt, and IL-17 signaling pathways) and cell death-related pathways (apoptosis, ferroptosis, and necroptosis). These results provide reliable evidence that AMC water participates in the regulation of host intestinal physiological functions and metabolic pathways by improving microbiota composition.

Which mediators mediate the bidirectional communication between the HPA axis and the gut microbiota? It has been well documented that the stress hormone COR directly causes changes in the gene expression profiles of the oral microflora, providing a possible pathway by which COR can alter the microbe [54]. Our findings also showed that the HPA axis affected the gut microbiota by controlling hormone secretion; however, what mediators does the gut microbiota use to regulate the HPA axis? The results of this study showed that the gut microbial metabolite LPS may be a messenger of the gut microbiota-brain axis. Mechanically, AMC water increases the abundance of probiotics *L.helveticus* and *R. gnavus*, which then decreases the level of fecal LPS and restricts its entry into the systemic circulation, thus inhibiting the content of pro-inflammatory factors in the hypothalamus. Also, the results of the correlation analysis in this study demonstrated that gut microbiota regulated the HPA axis through LPS-mediated pro-inflammatory factor expression in the hypothalamus.

The majority of studies showed that the primary target organ of weaning stress is the intestine, which causes intestinal barrier disruption, inflammatory damage, and microbial dysbiosis [4], [31]. To explore whether AMC water-induced improvement in microbiota composition alleviated weaning stress-induced gut damage, we assessed MS piglet gut morphology. The results of SEM and HE staining revealed that weaning caused apparent damage to the intestinal epithelium, a reduction of VH/CD ratio and a decrease in microvilli density in all small intestinal segments of piglets, which was significantly repaired by AMC water treatment. The gold standard for evaluating gut morphology is intestinal VH and the VH/CD ratio [55]. It exerts a fundamental effect on nutrient absorption and builds a defensive barrier. In addition, the decreased VH/CD induced by weaning may cause absorption dysfunction and further suppress intestinal cell proliferation and differentiation [56], [57]. Our results found that drinking AMC water significantly increased the number of goblet cells and Paneth cells in the small intestine. ISC differentiate into a variety of specialized IEC, among which goblet cells and Paneth cells exhibit a vital role in intestinal injury repair and host immunity [26]. Goblet cells are directly regulated by the immune system and secrete protective Mucs, which form a mucus layer between IEC and host microbes, and exert protection effect to against shear stress and injury [34]. In line with this finding, *Barodon* (an anionic AMC in Korea) application research showed that adding *Barodon* to diets for olive flounder could improve the feed utilization and digestibility by promoting the number of goblet cells in small intestine, as well as having positive effects on gut histology and innate immunity [58]. Likewise, Paneth cell exerts a function in innate immunity and antibacterial defense, to which ultimately secretes bactericidal defensin peptides and lysozymes [35]. Accumulating evidence indicated that Paneth cells are the marker molecules of Wnt/β-catenin pathway activation, which can reflect the dynamic activity of this pathway, thereby regulating the proliferation and recovery of epithelial cells [59]. Furthermore, the increased mRNA expression of Mucs and defensins also confirmed the changes in the number of Paneth cells and goblet cells. These results indicated that AMC water induced ISC differentiation toward goblet cells and Paneth cells, which further promoted the repair of weaning-induced intestinal damage.

Given that the differentiation and regeneration of ISC strictly depend on the activity of Wnt/β-catenin signaling pathway, we further measured the expression levels of related factors in Wnt/β-catenin pathway in the small intestine. Mechanically, after Wnt activated by R-sponds, β-catenin first aggregates in the protoplasm and then enters the nucleus, where it replaces Groucho (from TCF/LEF transcription factors) to form the TCF/LEF-β-catenin complex, which then regulates the expression of target genes, such as c-Myc, cyclin D1, and Lgr5. Previous study has shown that high levels of β-catenin are critical for intestinal homeostasis in intestinal epithelial regeneration [37]. Study found that [36] activation of Wnt/β-catenin signaling by hydrolyzed wheat gluten could relieve deoxynivalenol-induced gut damage via boosting ISC proliferation and differentiation in mice. Consistent with these findings, we observed that AMC water stimulated the Wnt/β-catenin signaling pathway, as reflected by the elevated levels of Wnt1, total/nuclear-β-catenin, Lrp6 and Lrp5, as well as decreased activity of β-catenin degradation complex. Notably, during intestinal epithelial damage repair, precursor cells dedifferentiate into new Lgr5 + ISC, which initiate a vast program of cell division and are involved in recovering epithelial integrity [60]. In our study, results argued that AMC water could significantly facilitate ISC proliferation, as manifested by increased density of Lgr5+, and proteins and mRNA expression of proliferating cell markers (Ki67 and Cyclin D1). On the other hand, the ISC proliferation induced by AMC water was confirmed by the changes in mRNA levels of active and quiescent ISC markers. Because ISC are the driving force behind the IEC's continual renewal and regeneration and are sensitive to external stimuli, it was not unexpected that weaning inhibited ISC growth. Trustworthy, these results indicated that AMC water maintained the high activity of Wnt/β-catenin signaling to promote the proliferation and differentiation of ISC, and recover the periodical self-renewal of intestinal epithelium, and consequently alleviated intestinal damage in weaned piglets. In surprise, RDA results (Supplementary Fig. S3) indicated that the elevation of *L. helveticus* abundance with AMC treatment was consistent with the activity of Wnt/β-catenin signaling, and the number of goblet cells, Lys + and Lgr5 + cells in the small gut. Evidently, AMC water-induced gut damage repair by stimulating the proliferation and differentiation of ISC via activating Wnt/β-catenin signaling pathway was at least partly attributed to the increase of beneficial bacteria abundance especially *L. helveticus*.

The IPEC-J2 cell line was originally isolated from the jejunal epithelium of neonatal piglets and consisted of undifferentiated porcine intestinal crypt-based columnar cells, reflecting the function of piglet intestinal epithelial cells under normal physiological conditions [61]. Thus, we performed in vitro evaluations using IPEC-J2. Consistent with the results of animal experiment, AMC alleviated the LPS-induced IPEC-J2 proliferation inhibition and decreased Lgr5 + cell ratio by activating the Wnt/β-catenin signaling pathway. Of note, AMC cannot reverse LPS-suppressed cell differentiation in the absence of gut microbiota, which indicates that AMC-induced improvement in microbiota composition is a key factor in stimulating ISC differentiation into goblet and Paneth cells.

## Conclusion

Taken together, we found, for the first time that AMC water confers diarrhea resistance in MS piglets under weaning stress by regulating the brain-microbe-gut axis. Further analysis showed that drinking AMC water inhibited stress hormones secretion by alleviating the HPA axis disorder, thereby ameliorating the gut microbe composition, as manifested by the increase in the abundance of probiotics *L.helveticus* and *R.gnavus*. The observed improvements in gut microbiota composition and function maintains intestinal epithelial regeneration by activating Wnt/β-catenin signaling pathway to repair damaged intestinal mucosa and interrupted ISC differentiation. Our study provides a potential prevention strategy for young mammals at risk of diarrhea.

## Compliance with Ethics Requirements

*Ethics requirements for the trial was received from the Northeast Agricultural University Animal Care and Use Committee (No. NEAUEC202102415)*.

## CRediT authorship contribution statement

**Jian Chen:** Conceptualization, Investigation, Writing – original draft. **Bi-Chen Zhao:** Methodology, Formal analysis. **Xue-Yan Dai:** Methodology, Formal analysis. **Ya-Ru Xu:** Visualization, Supervision. **Jian-Xun Kang:** Conceptualization, Methodology, Writing – review & editing. **Jin-Long Li:** Writing – review & editing, Data curation, Methodology.

## Declaration of Competing Interest

*The authors declare that they have no known competing financial interests or personal relationships that could have appeared to influence the work reported in this paper.*
