# Supplementary material for: Drinking alkaline mineral water confers diarrhea resistance in maternally separated piglets by maintaining intestinal epithelial regeneration via the brain-microbe-gut axis
Source: J Adv Res. 2022 Dec 17;52:29–43. doi: 10.1016/j.jare.2022.12.008 (PMC10555785; doi:10.1016/j.jare.2022.12.008)
Supplement: Supplementary data 1 [file mmc1.docx]

***Supplemental Materials***

**Drinking alkaline mineral water confers diarrhea resistance in maternally separated piglets by maintaining intestinal epithelial regeneration via the** **brain-microbe-gut axis**

**Jian Chen^a^, Bi-Chen Zhao^a^, Xue-Yan Dai^a^, Ya-Ru Xu^a^, Jian-Xun Kang^a^, Jin-Long Li^a,b,c,#^**

^a^ College of Veterinary Medicine, Northeast Agricultural University, Harbin 150030, P.R. China

^b^ Key Laboratory of the Provincial Education Department of Heilongjiang for Common Animal Disease Prevention and Treatment, Northeast Agricultural University, Harbin 150030, P.R. China

^c^ Heilongjiang Key Laboratory for Laboratory Animals and Comparative Medicine, Northeast Agricultural University, Harbin 150030, P.R. China

^#^**Corresponding author**

**Jin-Long Li**

Address: College of Veterinary Medicine, Northeast Agricultural University, Harbin, 150030, P. R. China

Tel: +86 45 155190407; fax: +86 451 55190407

E-mail address: Jinlongli@neau.edu.cn (J.L. Li)

**Supplementary Table S1. The composition of AMC concentrate**

| ingredient | Content | Chemical formula | |
| --- | --- | --- | --- |
| Sodium metasilicate pentahydrate | 200 g/L | | 5H_2_O·Na_2_SiO_3_ |
| Potassium bicarbonate | 100 g/L | | KHCO_3_ |
| Zinc oxide | 10 mg/L | | ZnO |
| Bis-(carboxyethylgermanium) sesquioxide | 1 mg/L | | Ge-132 |

**Supplementary Table S2. The mineral ions content of drinking water (AMC water and basal water)**

|  | AMC water | Basal water | |
| --- | --- | --- | --- |
| Ions | calculated contents (mg/L) | | analyzed contents (mg/L) |
| SiO_3_^2-^ | 179.25 | | ND^1^ |
| Na^+^ | 108.49 | | 2.79 |
| K^+^ | 97.50 | | 0.95 |
| Zn^2+^ | 0.02 | | ND |
| Ge^4+^ | 0.0005 | | ND |
| HCO_3_^-^ | 152.50 | | 13.20 |

^1^ ND, not detected

**Supplementary Table S3. Primers used for qRT-PCR analysis**

| Gene name | Accession number | Primer and probe sequences (5′ to 3′) |
| --- | --- | --- |
| GAPDH1 | NM_001206359.1 | F: TCGGAGTGAACGGATTTGGC  R: TGACAAGCTTCCCGTTCTCC |
| GAPDH2 | NM_001206359.1 | F: CGGAGTGAACGGATTTGGC  R: CACCCCATTTGATGTTGGCG |
| CRH | NM_001113062.1 | ACTCAGAGCCCAAGTCCGTT |
|  |  | TTAGGGGCGCTAGCTTCTGA |
| ARC | XM_021088786.1 | GAGAGCCGAAGCTGAAAATGC |
|  |  | AGGAGCCTAGAGACCTGTTG |
| GR | XM_005652952.3 | GAAGGGGGCGGCTGTTTAC |
|  |  | CCATCAGTGGGTATCAGCTCT |
| MR | XM_021100657.1 | GTGGAAGGTAAATGTTCATGGCA |
|  |  | TATGTCGTCCCCTCCTCTGG |
| FKBP5 | NM_001315611.1 | CCTTCACCTGCAGACCCG |
|  |  | CCCCACTCGTTTGACAATCTTT |
| CRHR1 | NM_001144110.1 | TTTCCTCAACAGCGAGGTCC |
|  |  | GACTGCTGTGGACTGCTTGA |
| AVPR1B | XM_003130445.3 | GCAACCCCTGGATCTACCTG |
|  |  | CCCTGTCGCCCCCTAAAAG |
| IL-1β | NM_214055.1 | F: GAAAGCCCAATTCAGGGACC  R: GGCGGGTTCAGGTACTATGG |
| IL-6 | NM_001252429.1 | GACCCTGAGGCAAAAGGGAAA |
|  |  | AGGAAATCCTCAAGGCTGCG |
| TNF-α | NM_214022.1 | GCCCCCAGAAGGAAGAGTTTC |
|  |  | GGCATACCCACTCTGCCATT |
| Muc1 | XM_021089730.1 | F: GTGCCGACGAAAGAACTG |
|  |  | R: TGCCAGGTTCGAGTAAGAG |
| Muc2 | XM_021082584.1 | F: CTGTGTGGGGCCTGACAA |
|  |  | R: AGTGCTTGCAGTCGAACTCA |
| Muc3 | NM_010843.2 | F: CAAGAAGAGCGCAAAGCAGG |
|  |  | R: AGTGGAGGATGAAAGCCAGC |
| Muc4 | XM_021068272.1 | F: TTCACCTGTGTTCCTCACACC |
|  |  | R: AAAGAGTCCCTCAAGCTCCC |
| Muc5 | XM_021082583.1 | F: GCCGCCTATGAGGACTTCAA |
|  |  | R: GCTGAAGGGCAGCTGAATTG |
| Muc6 | XM_021082474.1 | F: GCCATCAAGATCCTCTCGGG |
|  |  | R: GTCGTCCTTCATGTTCCCGT |
| Wnt1 | XM_003126100.5 | F: TCGCCCAACTTCTGCACATA |
|  |  | R: AATGCTCCTAAGGCGAGTCC |
| Axin1 | XM_021086926.1 | F: AAGGTGCCGGGATTAAAGGG |
|  |  | R: CCAGAAGTCCAGCAGGTCAG |
| Axin2 | XM_021066736.1 | F: ACTCAGTAACAACCCGAGCG |
|  |  | R: AAGCTTTCGGCTGGGTAGAC |
| GSK-3β | NM_001128443.1 | F: CGAGACACACCTGCACTCTT |
|  |  | R: CCGGCATTAGTATCTGAGGCT |
| APC | NM_001206430.1 | F: GGAAATTCCCGGGGCAGTAA |
|  |  | R: TCCTGGTCCATGCCTTGTTC |
| Lrp6 | XM_021092344.1 | F: CGTGCCAGTTGGAGGTTTTG |
|  |  | R: TCCGAAGGCTGTGGATAGGA |
| Lrp5 | XM_021082719.1 | F: GACCCCTCCCTCTACAACCT |
|  |  | R: GTAGGGGTCTGAGTCCGAGT |
| C-myc | NM_001005154.1 | F: AAAAGGTCGGAATCGGGGTC |
|  |  | R: GTTTCTCCTCTGGCGTTCCA |
| Cyclin D1 | XM_021082686.1 | F: TCCCCACGCTTCTCTTTCAC |
|  |  | R: TGGTTTACACGCGGCTAAGT |
| Ki67 | NM_001101827.1 | F: AAAGCACCAGGCTTTACGGA |
|  |  | R: CCAGATACGGGCTGCTTGAA |
| Lgr5 | NM_001315762.1 | F: AAGCCTTTGTAGGCAACCCT |
|  |  | R: TGGTTTACACGCGGCTAAGT |
| Olfm4 | XM_003482903.4 | F: TCTTAAACCTCACCGTCCGC |
|  |  | R: GCGTCTCGAGCTTCTCTACC |
| Ascl2 | NM_001122991.1 | F: CTGACCAAGGGCTAGTGTGG |
|  |  | R: CTCGTCAAGCCTCCAAGTGT |
| Bmi1 | NM_001285971.1 | F: TGCAGCTCGCTTCAAGATGG |
|  |  | R: TTGCTGGTCTCCAGGTAACG |
| Msi | XM_021072064.1 | F: GTCTCGAGTCATGCCCTACG |
|  |  | R: CCATCGTCCAGGGGTGAGAG |

**Supplementary Table S4.** **Antibodies used in this study for western blot**

| Antibody name | Company | product category | Dilution ratio |
| --- | --- | --- | --- |
| Anti-POMC antibody | Wanlei | DF2655 | 1:800 |
| Anti-ACTH antibody | Wanlei | WL0215 | 1:1000 |
| Anti-CRHR1 antibody | Wanlei | A8409 | 1:1000 |
| Anti-GR antibody | Wanlei | WL02695 | 1:600 |
| Anti-MR antibody | Wanlei | A21058 | 1:800 |
| Anti-BDNF antibody | Wanlei | WL0168 | 1:800 |
| Anti-C-fos antibody | Wanlei | WL03699 | 1:1000 |
| Anti-GR antibody | Wanlei | WL02695 | 1:800 |
| Anti-PCSK1 antibody | SANTA | sc-100578 | 1:1000 |
| Anti-Wnt1 antibody | Affinity | AF5315 | 1:1500 |
| anti-β-catenin antibody | Abclonal | A19657 | 1:1000 |
| anti-Axin antibody | Bioss | bs-21731R | 1:800 |
| anti-GSK-3β antibody | Bioss | bs-0028R | 1:800 |
| anti-Apc antibody | Affinity | AF9039 | 1:800 |
| anti-Lrp6 antibody | Affinity | DF2995 | 1:1000 |
| anti-Lrp5 antibody | Affinity | AF4645 | 1:800 |
| anti-β-actin antibody | Abclonal | ACO26 | 1:2500 |
| anti-Lamin B antibody | Bioss | bs-23709R | 1:1500 |
| anti-Ki67 antibody | Abclonal | A2094 | 1:1000 |
| anti-Cyclin D1 antibody | Affinity | AF0931 | 1:800 |
| anti-Lgr5 antibody | Abclonal | A10545 | 1:1000 |
| HRP-conjugated secondary antibody^-^ | Bioss | bs-40295G-HRP | 1:3000 |


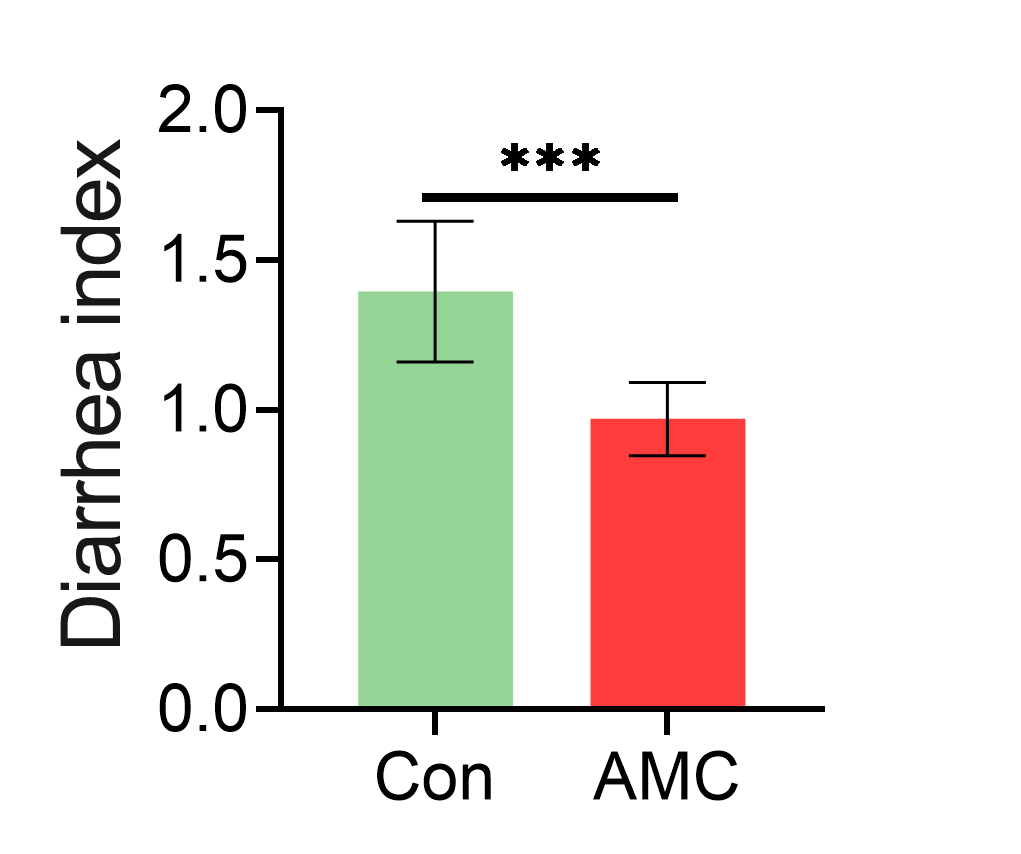


**Supplementary Fig. S1** The effect of AMC water on diarrhea index in MS piglet under weaning stress. Data are presented as the mean ± SD. ns, not significant, ^*^*P*<0.05, ^**^*P*<0.01, and ^***^*P*<0.001.


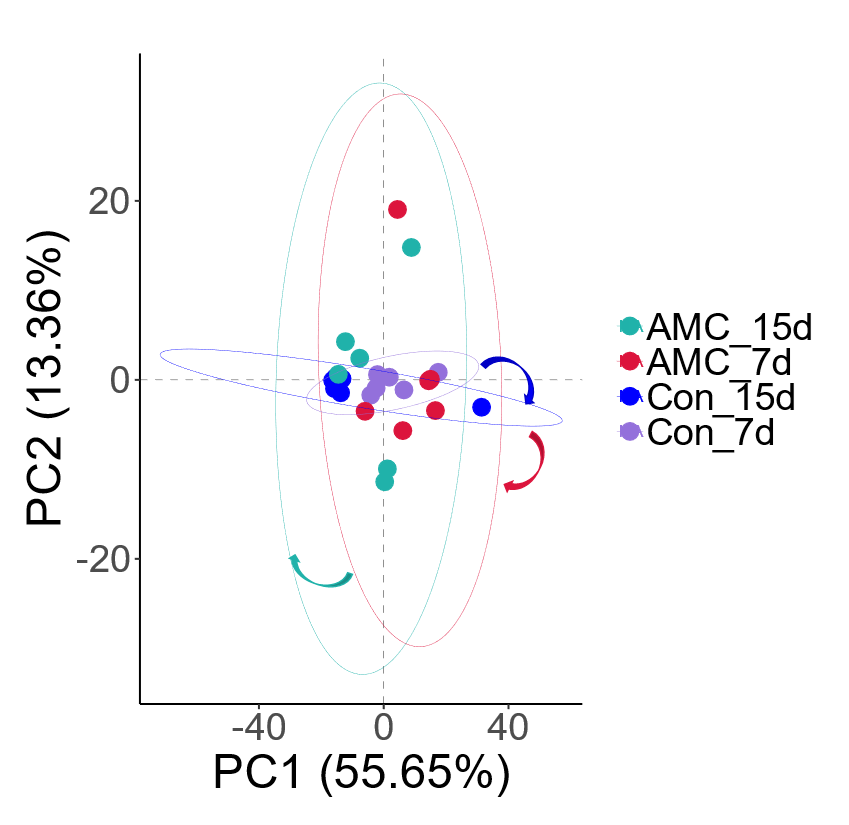


**Supplementary Fig. S2** Scatterplot from PCA based on KEGG pathway analysis by PICRUSt in intestinal bacterial communities of MS piglets.


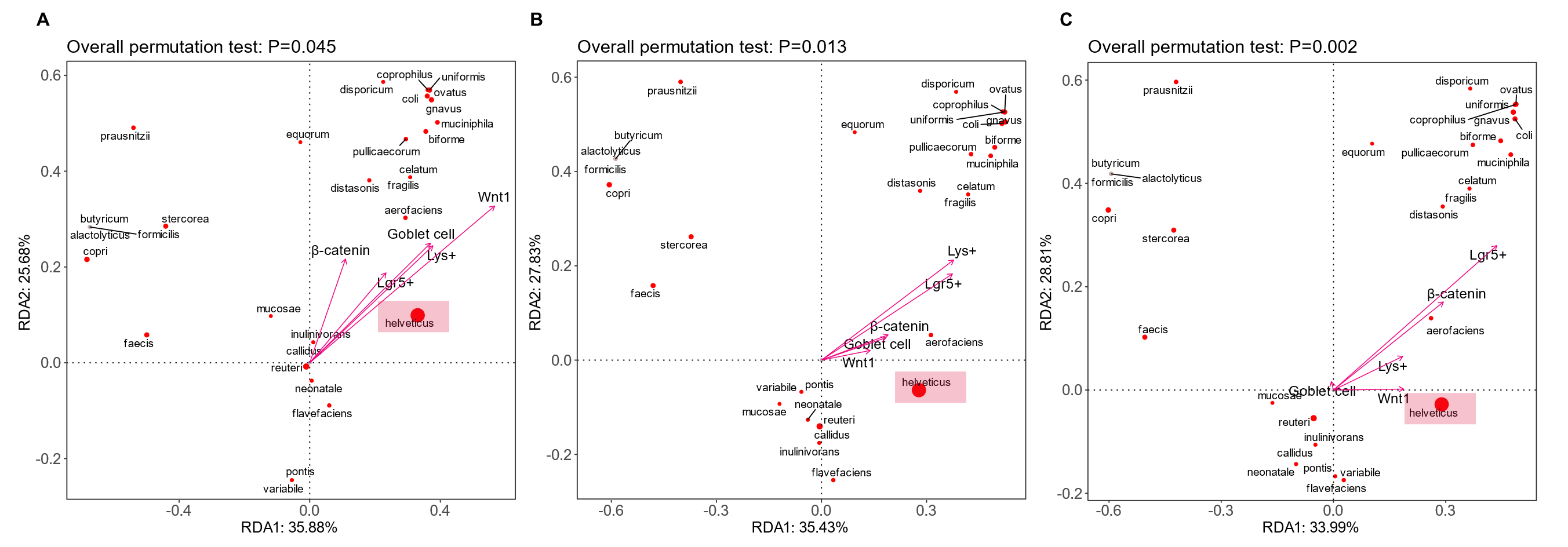


**Supplementary Fig. S3** Redundancy analysis (RDA) for the relevance of gut microbiota (at the species level) with phenotypic and molecular expression changes in the (B) duodenum, (C) jejunum, and (D) ileum of MS piglets.

**Supplementary Materials and Methods**

**Sample collection**

On days 7 and 15, MS piglet stool samples were collected for 16S rRNA MiSeq Sequencing of gut microflora. On day 15, one piglet from each pen (n = 6) was selected and slaughtered for sample collection. Through jugular venipuncture, blood samples were obtained, and serum was obtained as described in a previous report [1]. The piglets were killed by euthanasia, and the abdominal cavity was opened to collect the mid-section of the small intestine (duodenum, jejunum, and ileum) immediately. For paraffin section, frozen section, and scanning electron microscopy (SEM), slices from the mid-section of the duodenum, jejunum, and ileum were preserved in 4% paraformaldehyde or 2.5% glutaraldehyde (SEM). A 10 cm section was placed in Snap-frozen in liquid nitrogen and then stored at -80℃ for various analyses.

**ELISA assay**

The collected serum, faeces and hypothalamus tissues sample were used to measure HPA axis related hormones, LPS, and proinflammatory cytokines by the commercially available enzyme-linked immunosorbent assay (ELISA) kits from Beijing Chenglin Biological Technology Co. LTD. (Beijing, China) following the protocol. The levels of corticotropin releasing hormone (CRH), arginine vasopressin (AVP), adrenocorticotrophic hormone (ACTH), cortisol (COR), Haptoglobin (Hpt), lipopolysaccharide (LPS), IL-1β, IL-6, TNF-α and IFN-γ were quantified by using a microplate reader, and absorbance was determined at 450 nm.

**Small intestine morphology analysis**

As previously described, Hematoxylin and Eosin (HE) staining [2] and scanning electron microscopy (SEM) [3] were used to examine intestinal morphology. The Philips Model SU8010 FASEM (HITACHI, Japan) was used to SEM visualization. The villous height (VH) and crypt depth (CD) of each segment were assessed with Image J software, and the ratio of VH:CD was calculated. For each group, a minimum of 8 villi from each sample were determined.

**Periodic acid schiff staining**

To determine the number of goblet cells, the small intestine periodic acid-schiff (PAS) staining was conducted. Paraffin sections were cut into approximately 5-µm thick sections and stained with a PAS staining kit based on the provided instructions (No. G1281, Solarbio Science & Technology Co.,Ltd., Beijing, China). Briefly, the sections were dewaxed and oxidized with an oxidizing agent, then stained with Schiff and hematoxylin, and then dehydrated and transparent for observation by microscope (DM2000 LED, Leica, Germany).

**5-ethynyl-2′-deoxyuridine (EdU) incorporation assay**

Cells from the Con, LPS and LPS+AMC groups were cultured in 6-well plates at 1× 10^6^ cells/ml in the growth medium. Propagation of cells was cytochemically detected according to the manufacturer’s instructions (C0071L, Beyotime, China). Briefly, IPEC-J2 cells were incubated with the EdU staining buffer for 2.5 h, fixed by 4% polyformaldehyde and stained the nuclear with DAPI. The stained cells were scanned and photographed under Leica fluorescence microscope (DMi8, Leica, Germany). Furthermore, EdU^+^ cells ratio was used to assess EdU-positive cells with Image J software (National Institute of Health, USA).

**Quantitative Real-Time PCR**

Total RNA was extracted from mid-duodenum, mid-jejunum, mid-ileum, hypothalamus, and pituitary gland tissues (100 mg) using RNAout reagent following the enterprise’s description (Beijing Tiandi, Inc., Bejing, P.R. China). At 260/280 nm, the quality and concentration of RNA were committed spectrophotometrically. A commercial reagent kit was used to generate cDNA from 4 μg total RNA (product category: AU311-02, TransGen Biotech, Beijing, China) following the specification. The cDNA was deposited at −80℃ before the quantitative Real-Time PCR (qRT-PCR). The primers used in this study for qRT-PCR (Supplemental Table S3) were designed by the Primer Premier software 6.

**Western blot**

Total or nuclear protein was extracted from the mid-duodenum, mid-jejunum, and mid-ileum tissues or IPEC-J2 cells using RIPA Lysis buffer. Protein was separated by 6%-10% SDS-PAGE and transferred onto a PVDF or NC membrane. The detail procedure of the western blot is shown in our previous report [4]. The membranes were washed three times for 15 minutes each, imaged by Amersham Imager 600 (GE, Switzerland). Blots were semiquantified using Image J software (National Institute of Health, USA). The antibodies used in this study were shown in Supplemental Table S4.

**Immunofluorescence assay**

Small intestine sections were fixed in 4% paraformaldehyde overnight and then embedded in the optimal cutting temperature (OCT) compound. The duodenum, jejunum, and ileum were sectioned at 8 μm, rinsed in TBS, permeabilized with 0.4% Triton X-100 for 20 min, washed three times with TBS and incubated for 1.5 h in 10% normal goat serum to reduce nonspecific background. Sections were then incubated with the primary antibody at 4℃ overnight. The tissue sections were incubated with a goat anti-rabbit antibody conjugated to Alexa Fluor 594 (1:500, Abcam, ab150160) for 40 min at 37℃, and stained nuclei with DAPI for 10 min. The stained sections were observed with a Leica fluorescence microscope (DMi8, Leica, Germany) and the fluorescence images were captured for further qualitative and quantitative analysis. Analysis of β-catenin, Lgr5+, and lysozyme+ mean density in the small intestine was performed by Image J software to analyze the mean optical density. The methods used for immunofluorescence staining of cells were described previously report [5]. The following primary antibodies were used in immunofluorescence assays: anti-rabbit lysozyme, Lgr5, β-catenin, Muc2 (1:150, Abclonal, A13511, A10545, A19657, A14659) and Wnt (1:100, Affinity, AF5315) antibodies.

**16S** **rRNA PacBio SMRT Gene Full-Length Sequencing of Gut Microbiota.**

Total microbial genomic DNA of piglet fecal samples were extracted following the manufacturer’s instructions (TIANGEN Biotech Co., Ltd., Beijing, China, DP812) and stored at −20℃. The specific barcode sequencing primer design, gene amplification, and sequencing library (SMRTbell library) formation were done as described previously [6]. The formed library was checked, and the sequencing service was supplied by Wekemo Tech Group Co., Ltd., (Shenzhen, China) using PacBio Sequel System.

**Bioinformatics analysis**

Fastq file quality control, operational taxonomic unit (OTU) cluster and associated downstream analysis are consistent with previous [7]. Alpha diversity (Shannon and Chao1 indices) was measured using QIIME2, and the corresponding rarefaction curve are drawn by R (v3.1.1) software. Beta diversity analysis based on weighted UniFrac distance was conducted using QIIME (v1.80) software and shown by the principal coordinates analysis (PCoA). The Linear discriminant analysis Effect Size (LEfSe) analysis was performed by LEfSe software to find the bacteria with marked differences in relative abundance between the two treatments. To predict the gene family abundances of bacterial communities, the phylogenetic investigation of communities by reconstruction of unobserved states (PICRUSt) method was performed according to previous study [8]. The functional correlation network analysis was conducted to assess the correlation of microflora using Cytoscape 3.4.0 as previously described [9]. Redundancy analysis (RDA) was performed by the bioincloud platform (<https://www.bioincloud.tech/task-meta>).

**Supplementary References**

[1] J. Chen, Y.R. Xu, J.X. Kang, B.C. Zhao, X.Y. Dai, B.H. Qiu, J.L. Li, Effects of alkaline mineral complex water supplementation on growth performance, inflammatory response and intestinal barrier function in weaned piglets, J. Anim. Sci. 100(10) (2022) skac251. Doi: 10.1093/jas/skac251.

[2] M.Z. Li, Y. Zhao, H.R. Wang, M. Talukder, J.L. Li, Lycopene Preventing DEHP-Induced Renal Cell Damage Is Targeted by Aryl Hydrocarbon Receptor, J Agric Food Chem 69(43) (2021) 12853-12861. Doi: 10.1021/acs.jafc.1c05250.

[3] H. Yi, L. Zhang, Z. Gan, H. Xiong, C. Yu, H. Du, Y. Wang, High therapeutic efficacy of Cathelicidin-WA against postweaning diarrhea via inhibiting inflammation and enhancing epithelial barrier in the intestine, Sci Rep 6 (2016) 25679. Doi: 10.1038/srep25679.

[4] J. Chen, Y.X. Tang, J.X. Kang, Y.R. Xu, A.I.A. Elsherbeni, H.B.A. Gharib, J.L. Li, Astragalus polysaccharide alleviates transport stress-induced heart injury in newly hatched chicks via ERS-UPR-autophagy dependent pathway, Poult Sci 101(9) (2022) 102030. Doi: 10.1016/j.psj.2022.102030.

[5] J.Y. Zhou, D.G. Huang, M. Zhu, C.Q. Gao, H.C. Yan, X.G. Li, X.Q. Wang, Wnt/beta-catenin-mediated heat exposure inhibits intestinal epithelial cell proliferation and stem cell expansion through endoplasmic reticulum stress, J Cell Physiol 235(7-8) (2020) 5613-5627. Doi: 10.1002/jcp.29492.

[6] Q. Emu, H. Guan, J. Zhu, L. Zhang, J. Fan, Y. Ji, Y. Lin, C. Li, X. Dan, Y. Aguo, X. Wei, M. Zhang, B. Zhang, C. Yang, B. Li, C. Xiong, Grazing and Supplementation of Dietary Yeast Probiotics Shape the Gut Microbiota and Improve the Immunity of Black Fattening Goats (Capra hircus), Front Microbiol 12 (2021) 666837. Doi: 10.3389/fmicb.2021.666837.

[7] R.C. Edgar, Search and clustering orders of magnitude faster than BLAST, Bioinformatics 26(19) (2010) 2460-1. Doi: 10.1093/bioinformatics.

[8] M.G. Langille, J. Zaneveld, J.G. Caporaso, D. McDonald, D. Knights, J.A. Reyes, J.C. Clemente, D.E. Burkepile, R.L. Vega Thurber, R. Knight, R.G. Beiko, C. Huttenhower, Predictive functional profiling of microbial communities using 16S rRNA marker gene sequences, Nat Biotechnol 31(9) (2013) 814-21. Doi: 10.1038/nbt.2676.

[9] J. Zhang, X. Chen, P. Liu, J. Zhao, J. Sun, W. Guan, L.J. Johnston, C.L. Levesque, P. Fan, T. He, G. Zhang, X. Ma, Dietary Clostridium butyricum Induces a Phased Shift in Fecal Microbiota Structure and Increases the Acetic Acid-Producing Bacteria in a Weaned Piglet Model, J Agric Food Chem 66(20) (2018) 5157-5166. Doi: 10.1021/acs.jafc.8b01253.
