# Supplementary material for: Drinking alkaline mineral water confers diarrhea resistance in maternally separated piglets by maintaining intestinal epithelial regeneration via the brain-microbe-gut axis
Source: J Adv Res. 2022 Dec 17;52:29–43. doi: 10.1016/j.jare.2022.12.008 (PMC10555785; doi:10.1016/j.jare.2022.12.008)
Supplement: Supplementary data 2 [file mmc2.docx]

**Supplementary information of the full unprocessed and uncropped images of Western Blots**

1. **POMC (Pituitary gland)**


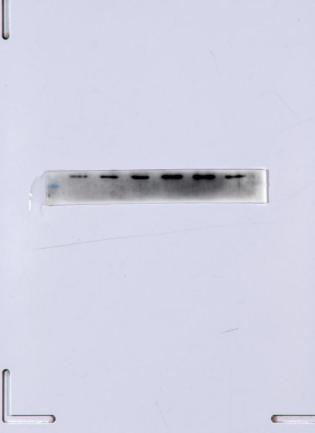

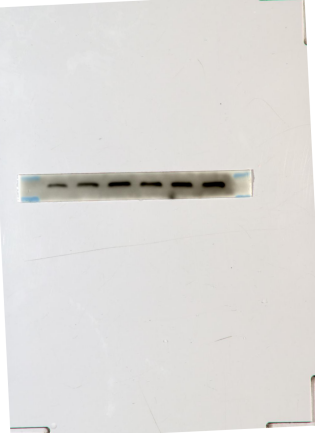

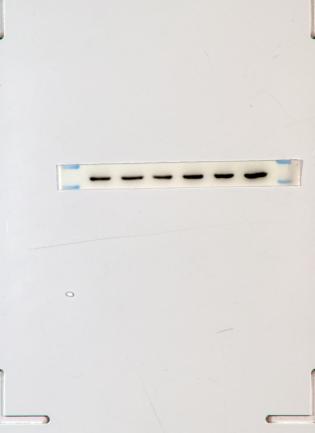


1. **ACTH (Pituitary gland)**


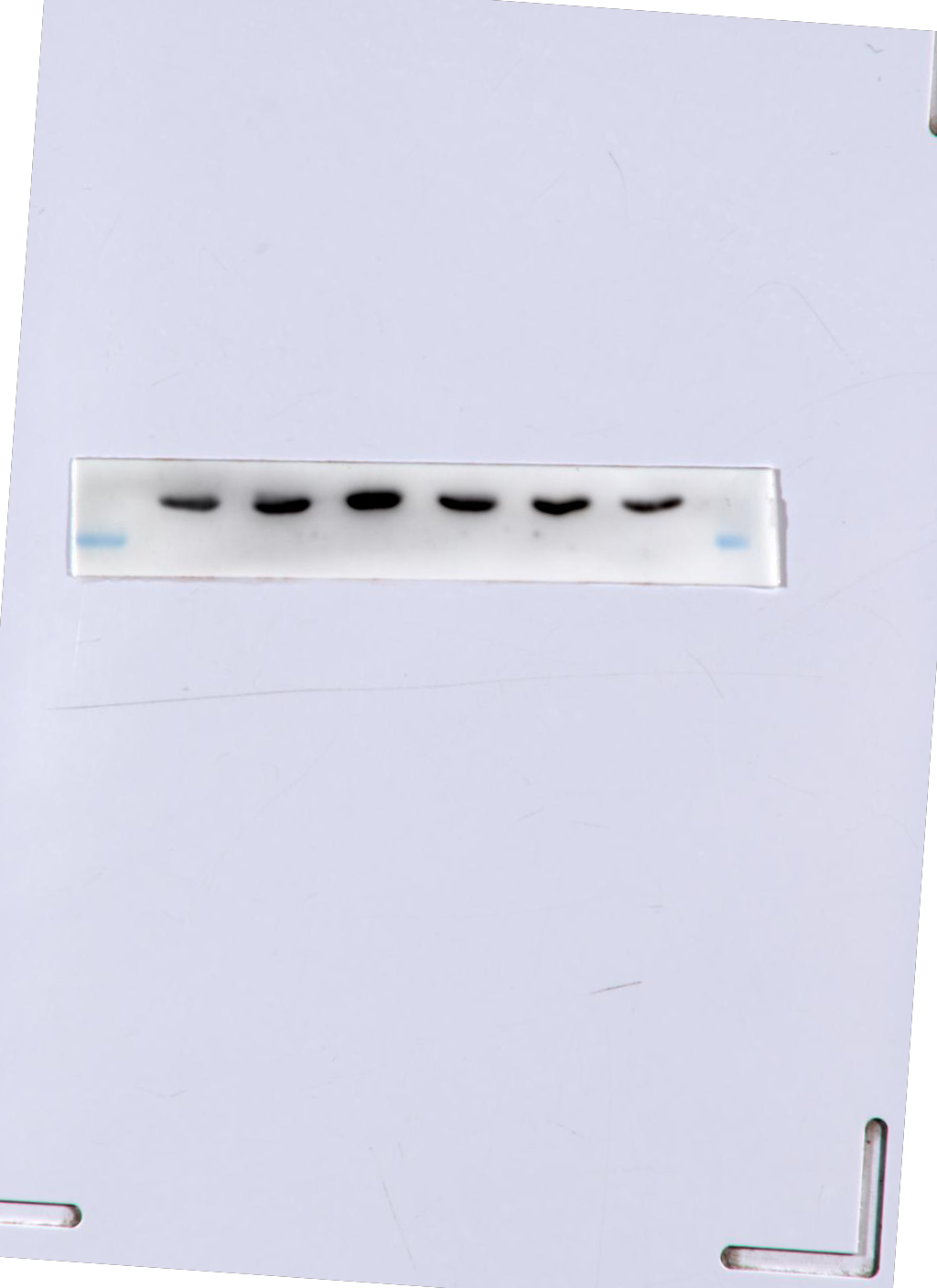

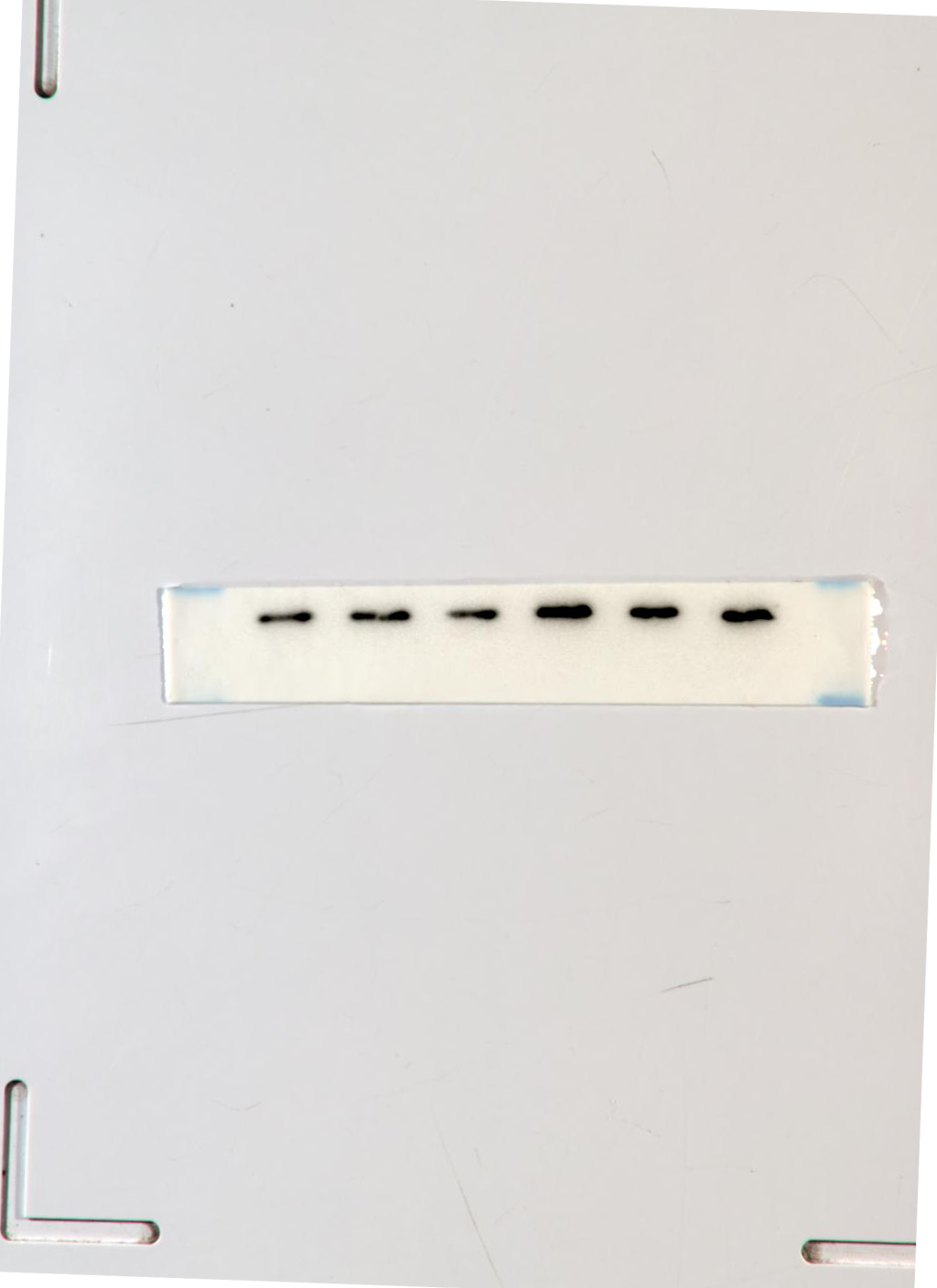

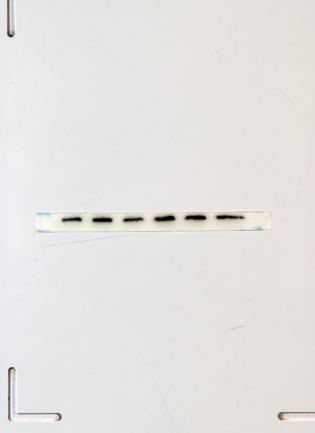


1. **CRHR1 (Pituitary gland)**


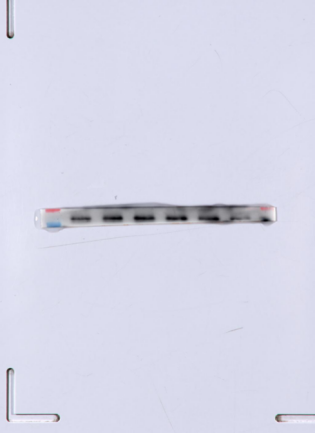

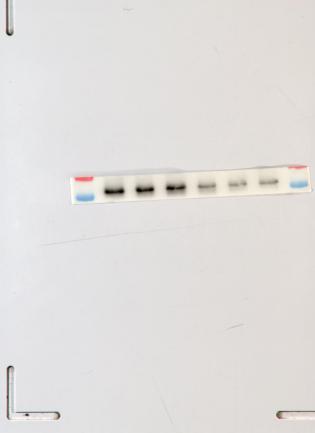

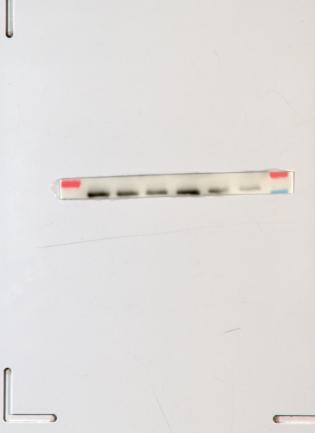


1. **β-actin (Pituitary gland)**


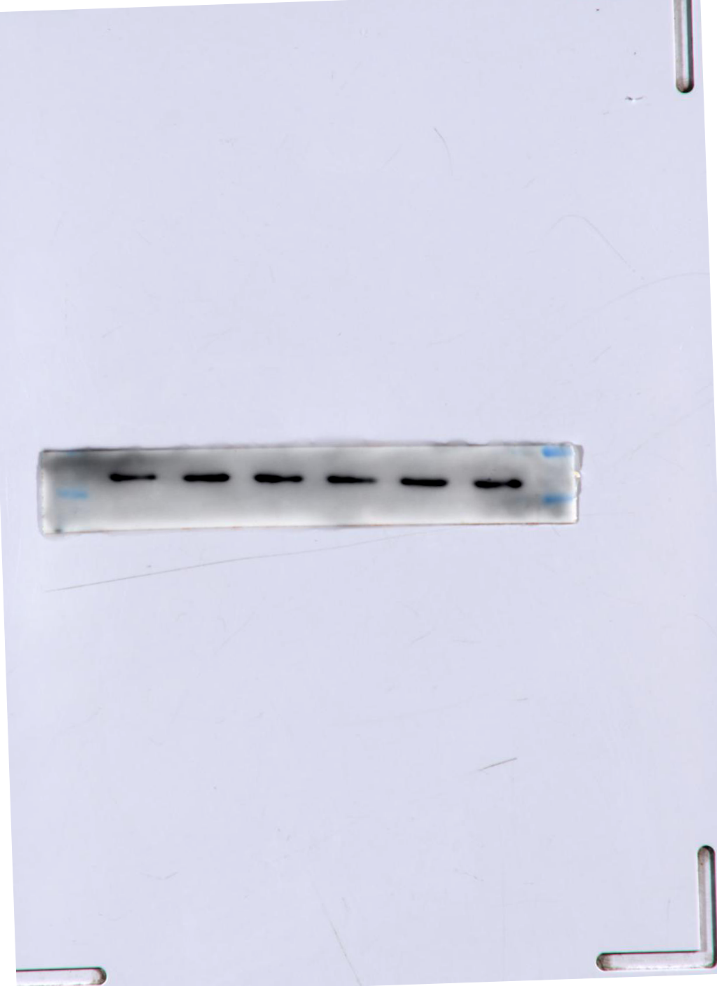


1. **GR (Hypothalamus)**


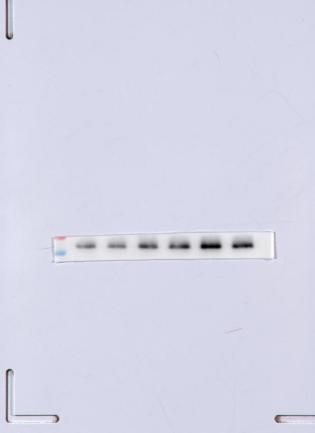

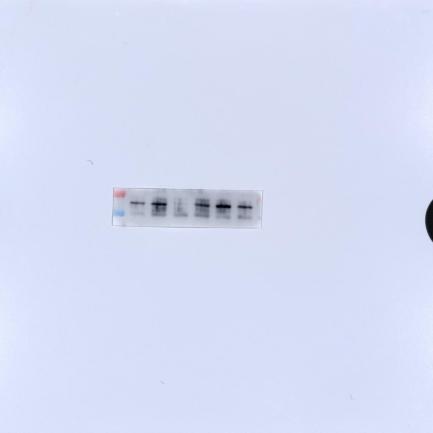

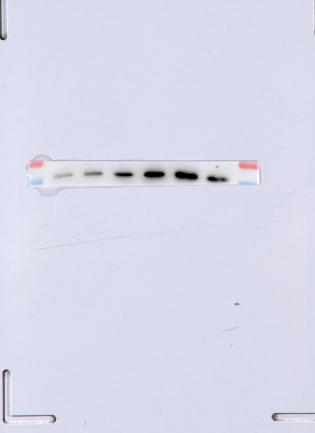


1. **MR (Hypothalamus)**


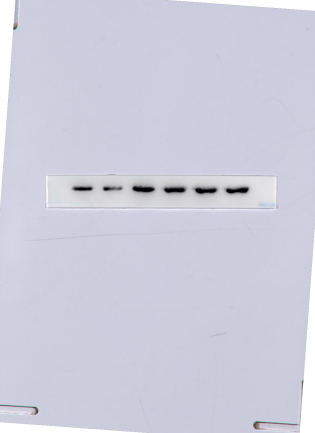

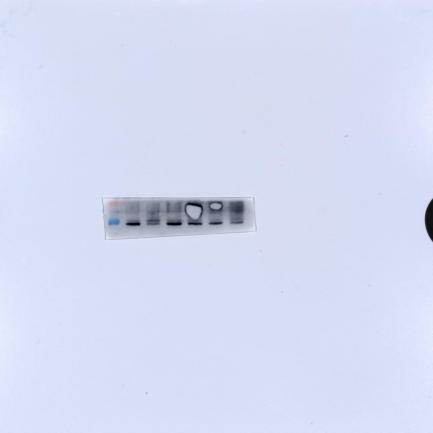

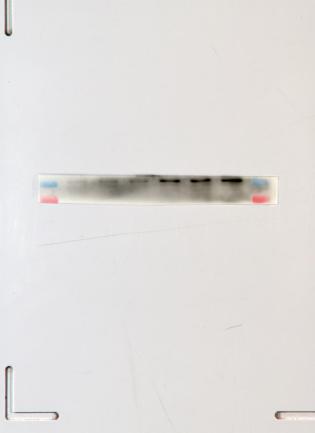


1. **BDNF (Hypothalamus)**


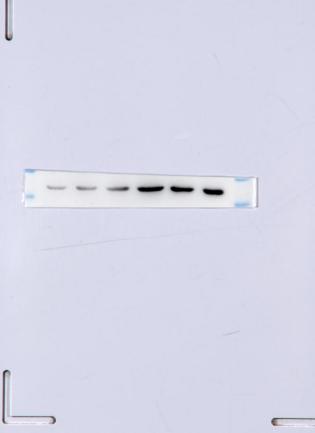

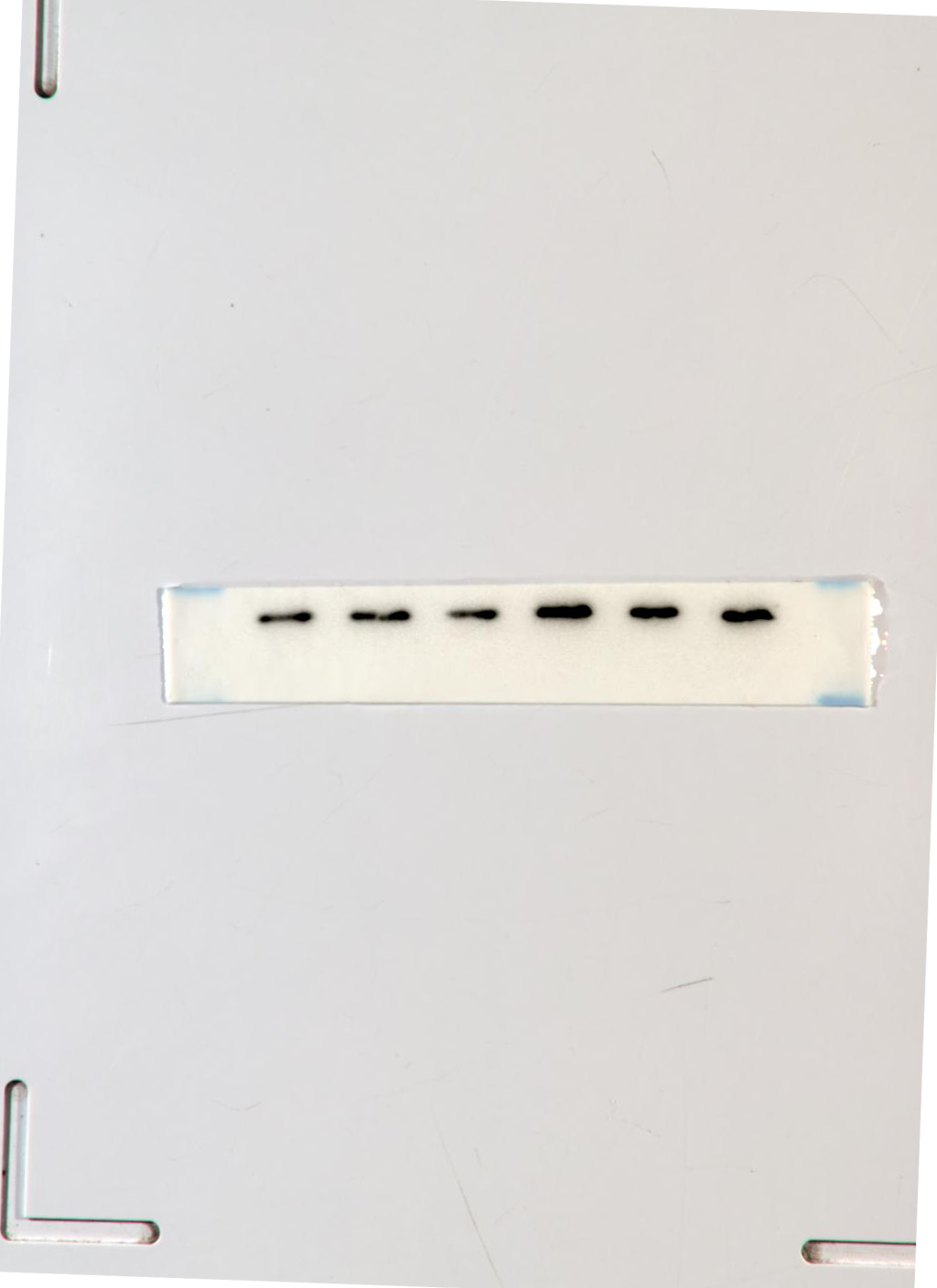

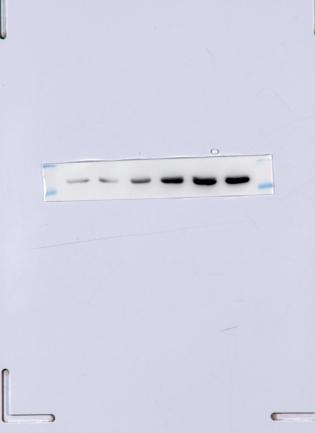


1. **C-fos (Hypothalamus)**

**
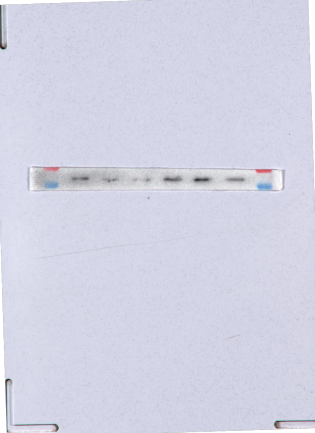

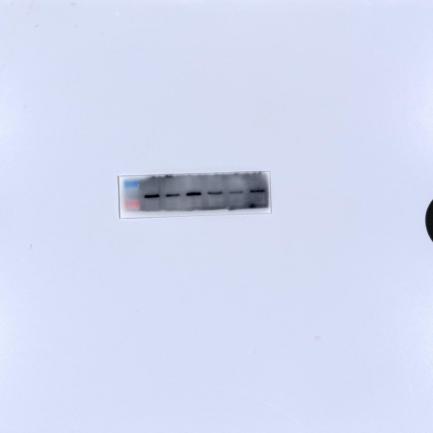
**
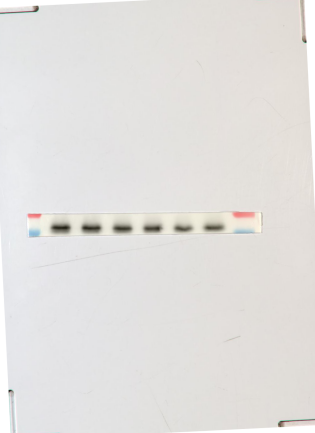


1. **β-actin (Hypothalamus)**

**
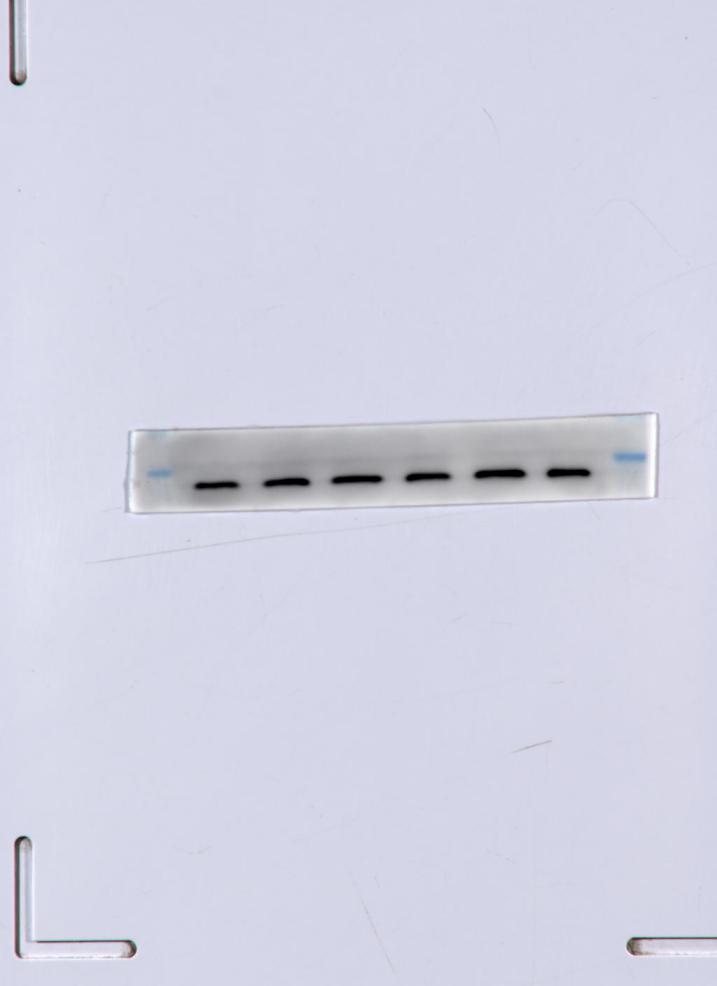
**

1. **Wnt1 (Duodenum, Jejunum, Ileum)**

**
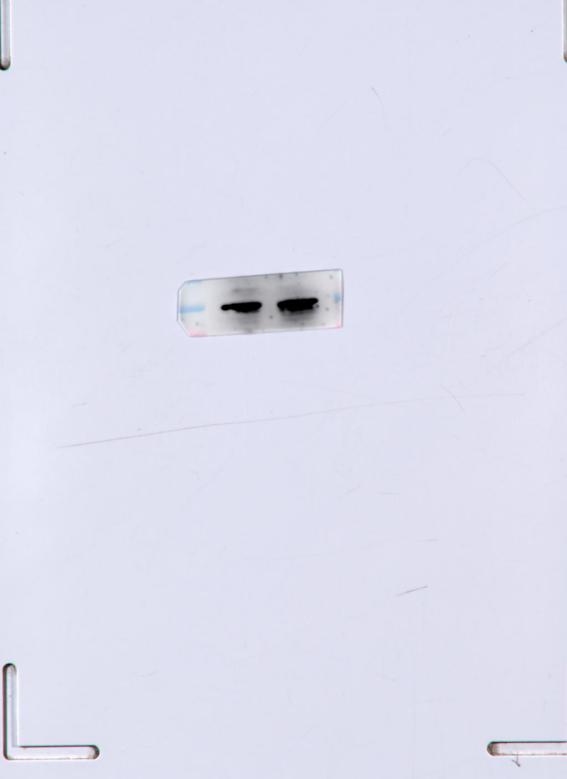
** **
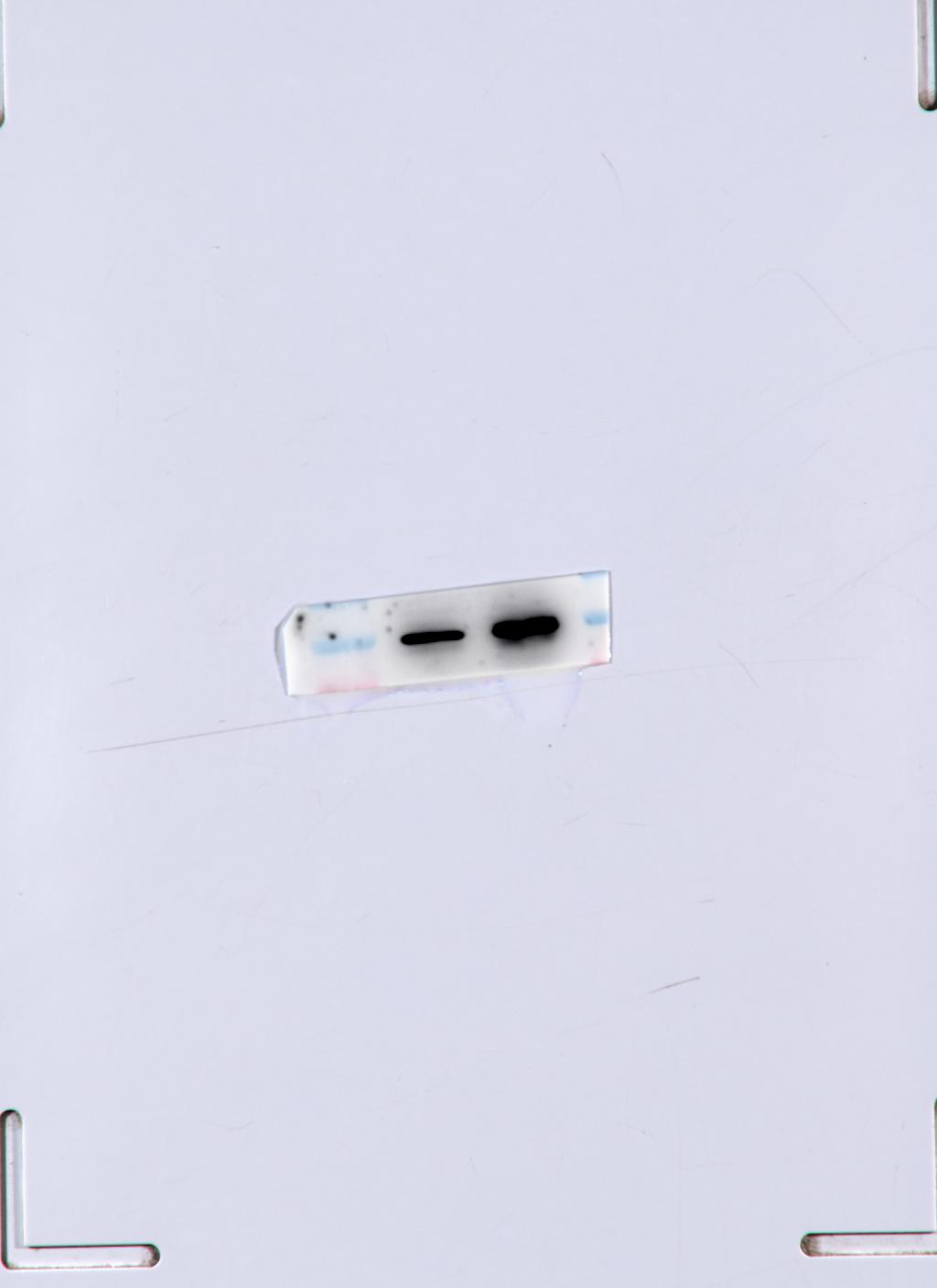
**
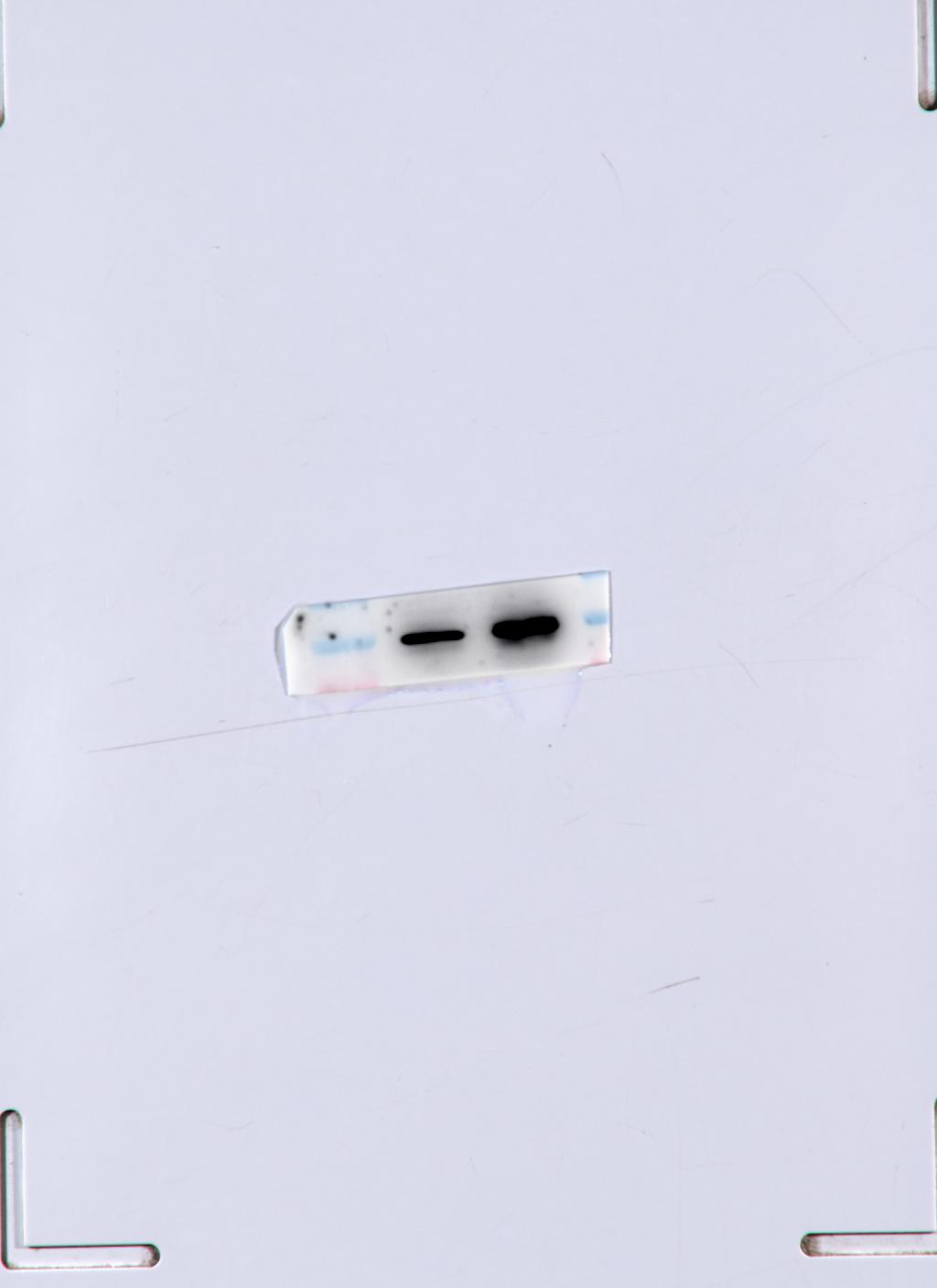


1. **β-catenin (Duodenum, Jejunum, Ileum)**

**
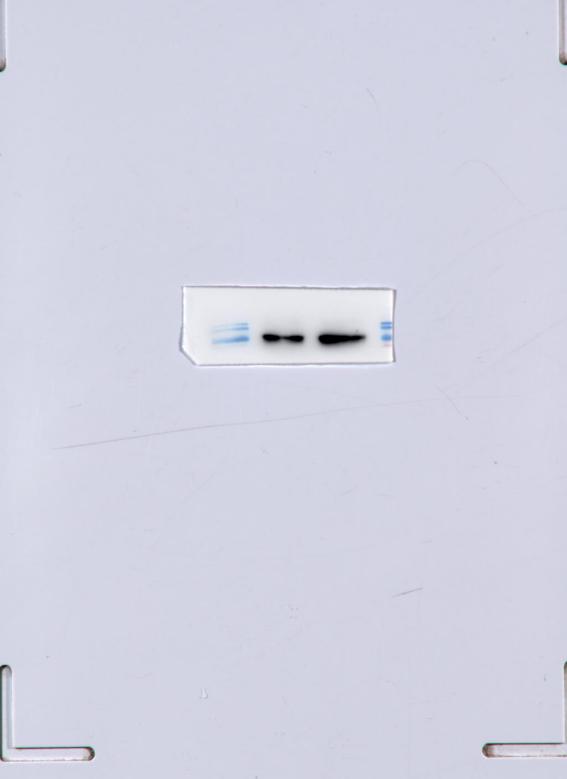
** **
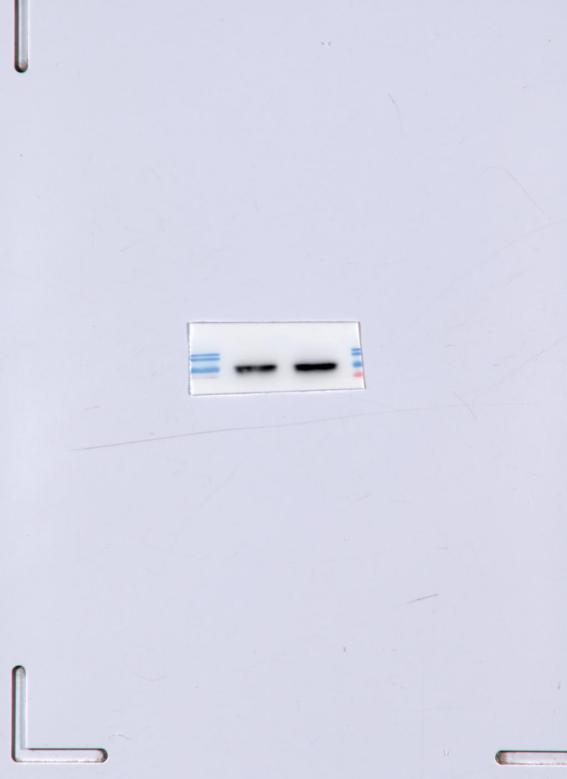
**

**
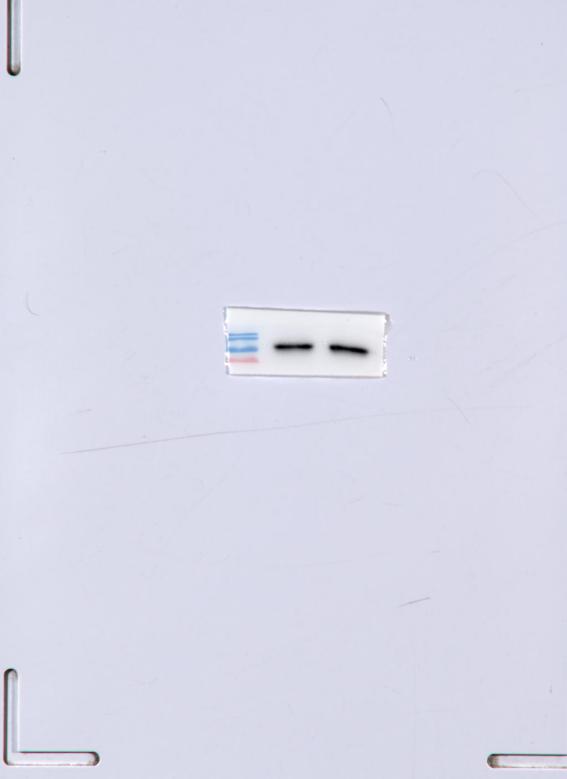
**

1. **Axin (Duodenum, Jejunum, Ileum)**

**
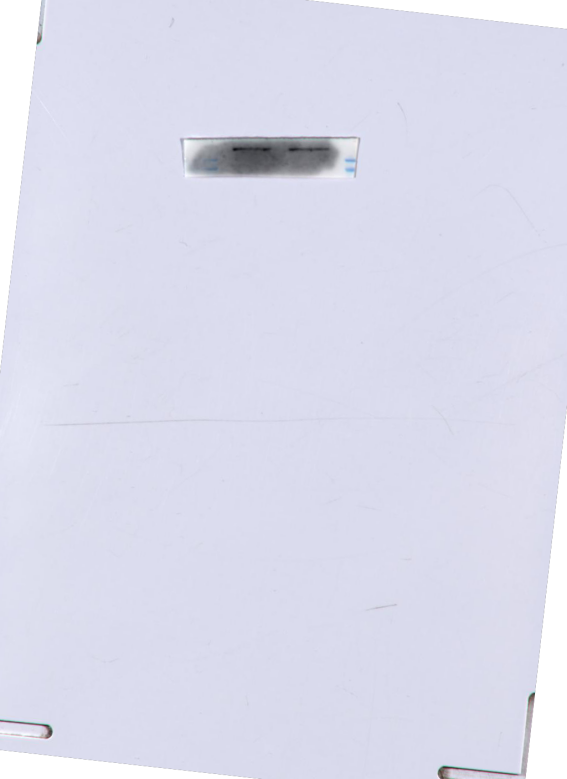
** **
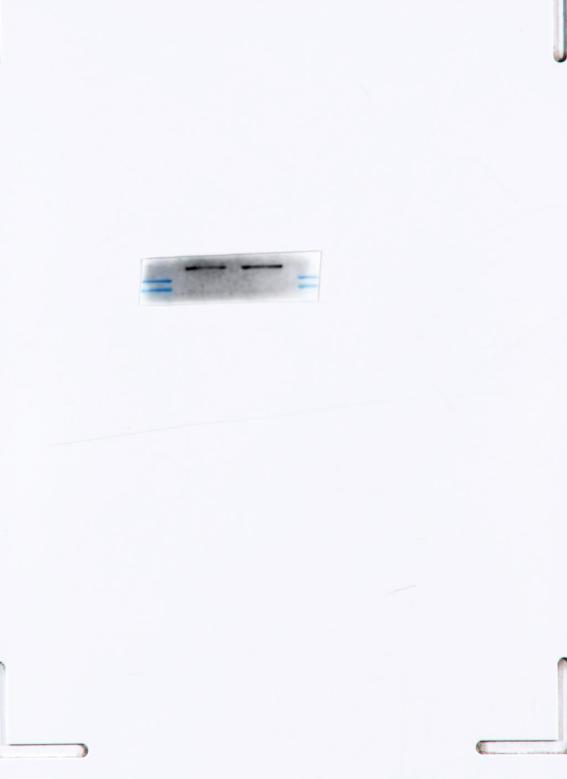
**
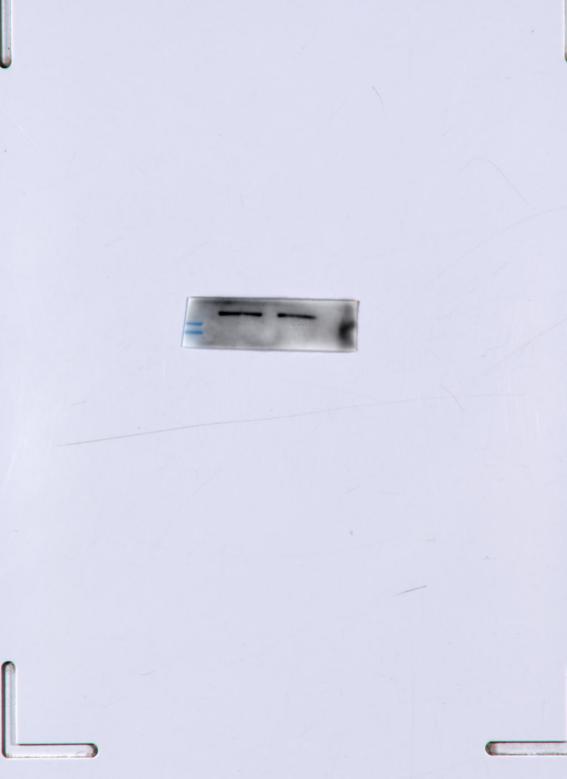


1. **GSK-3β (Duodenum, Jejunum, Ileum)**

**
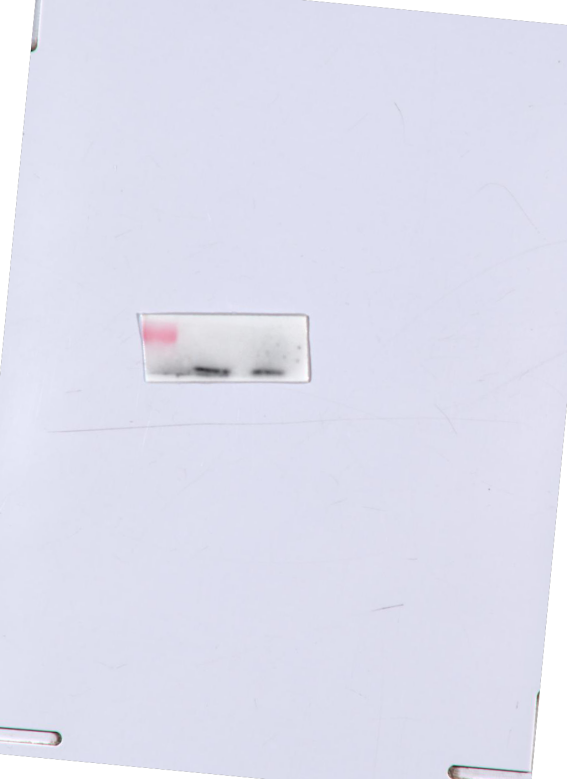
** **
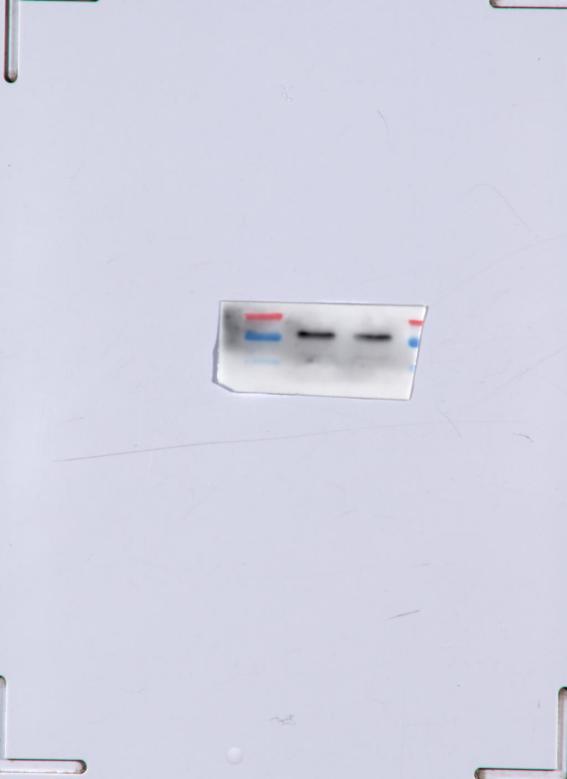
**

**
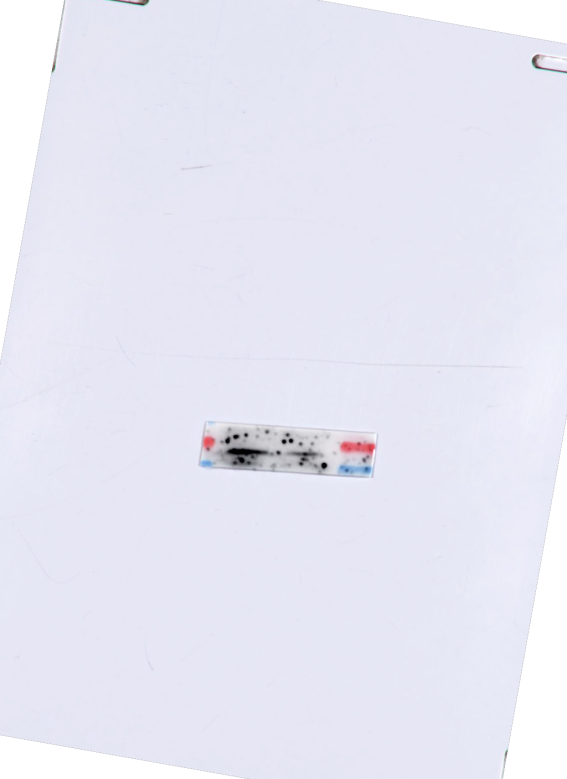
**

1. **APC (Duodenum, Jejunum, Ileum)**

**
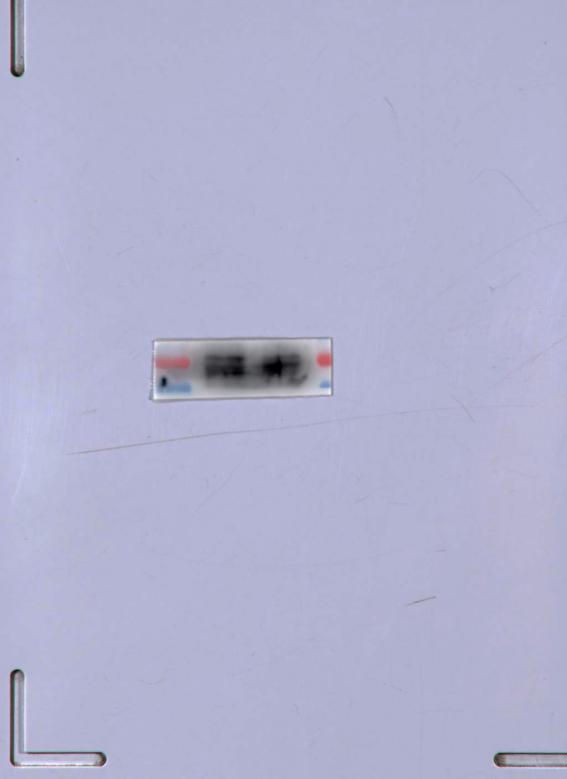
** **
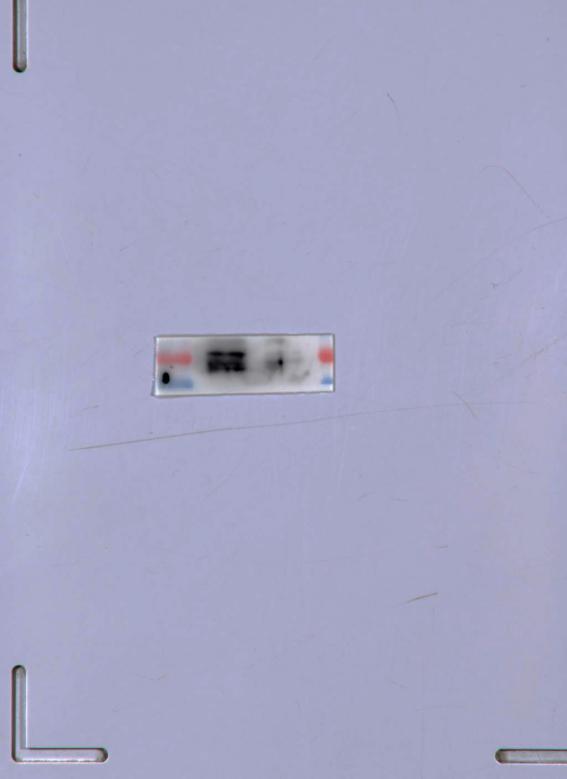
**

**
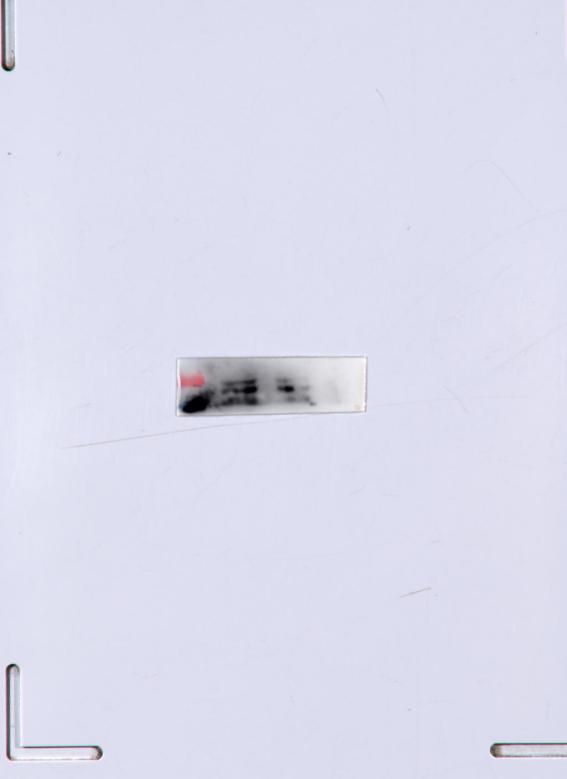
**

1. **Lrp6 (Duodenum, Jejunum, Ileum)**

**
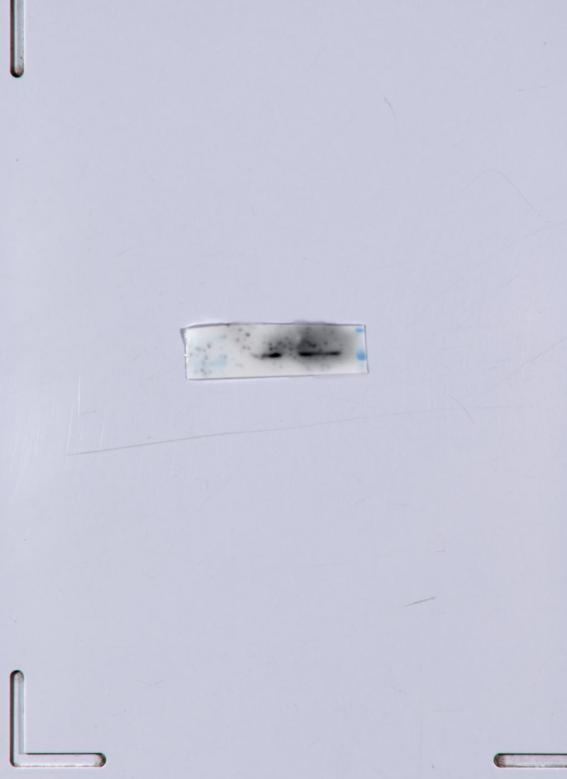
** **
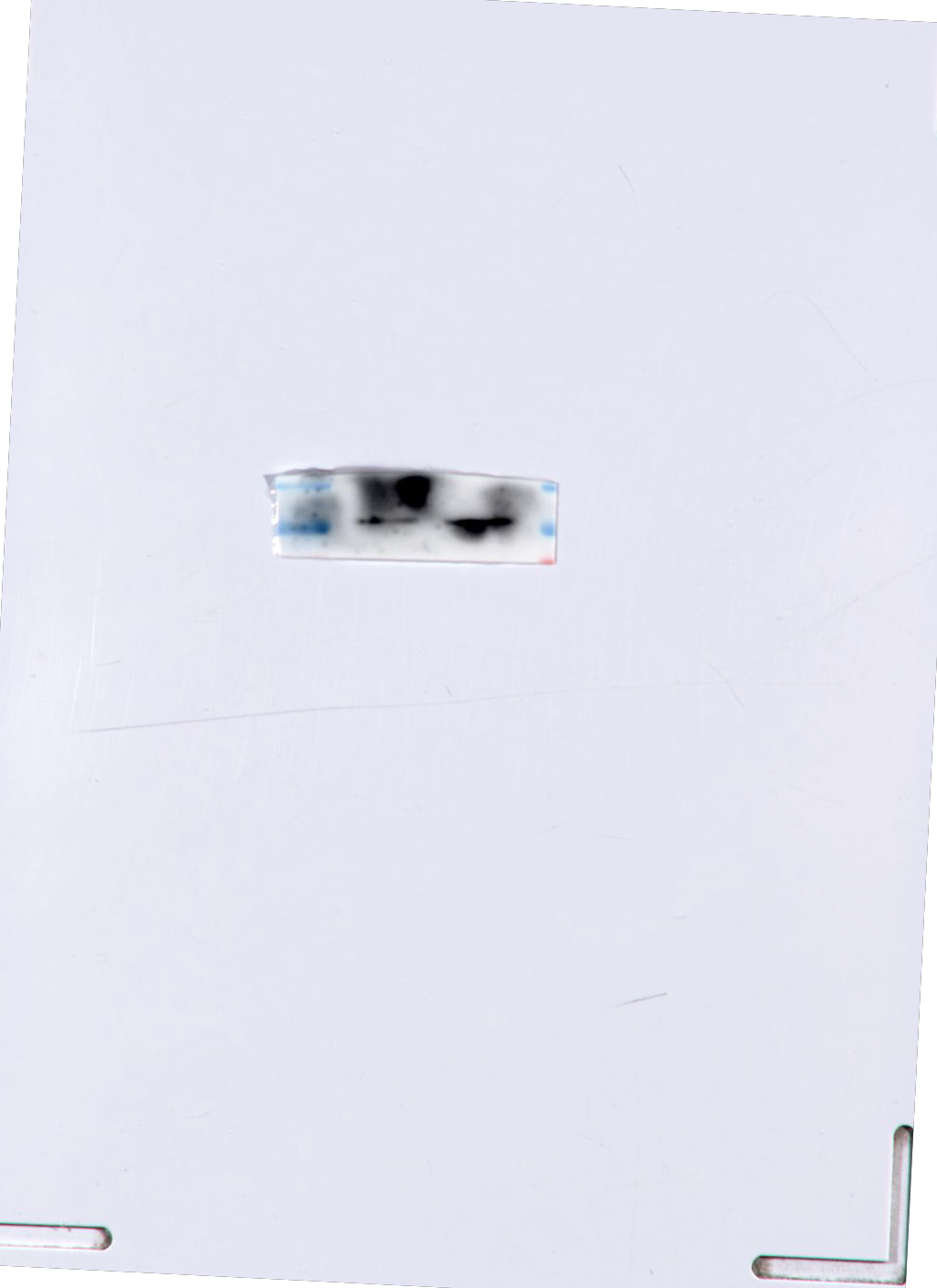
**
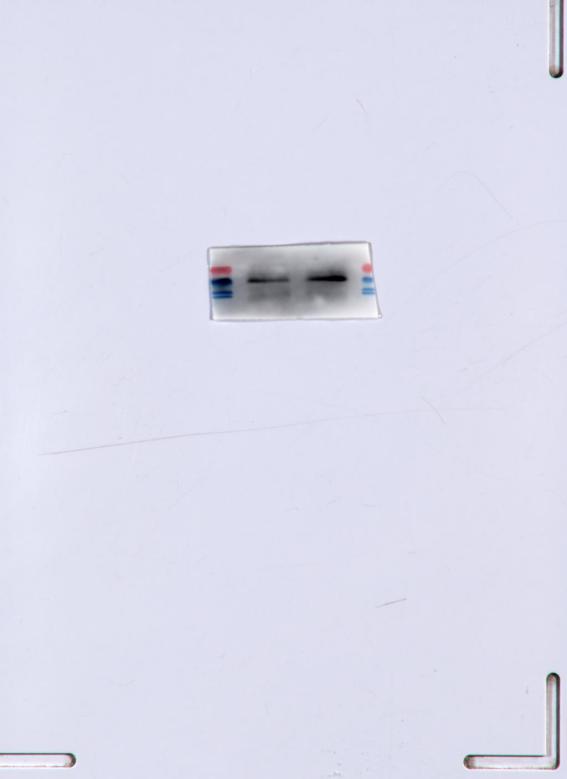


1. **Lrp5 (Duodenum, Jejunum, Ileum)**

**
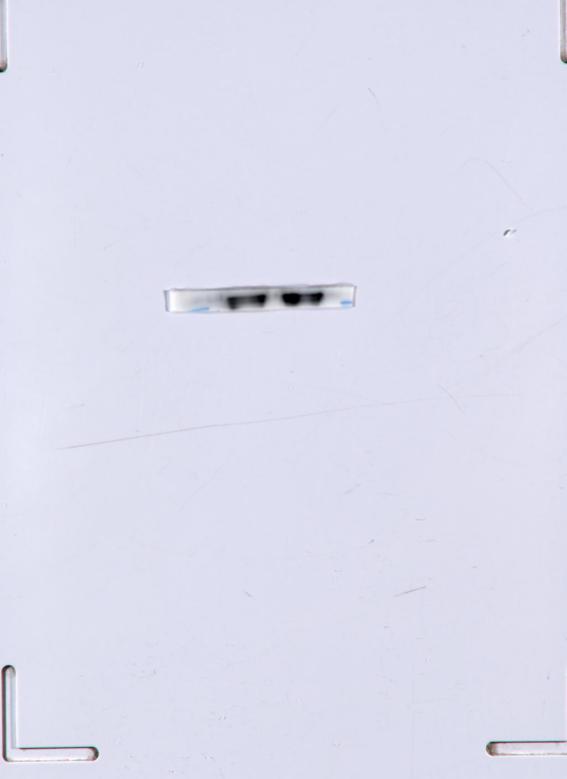
** **
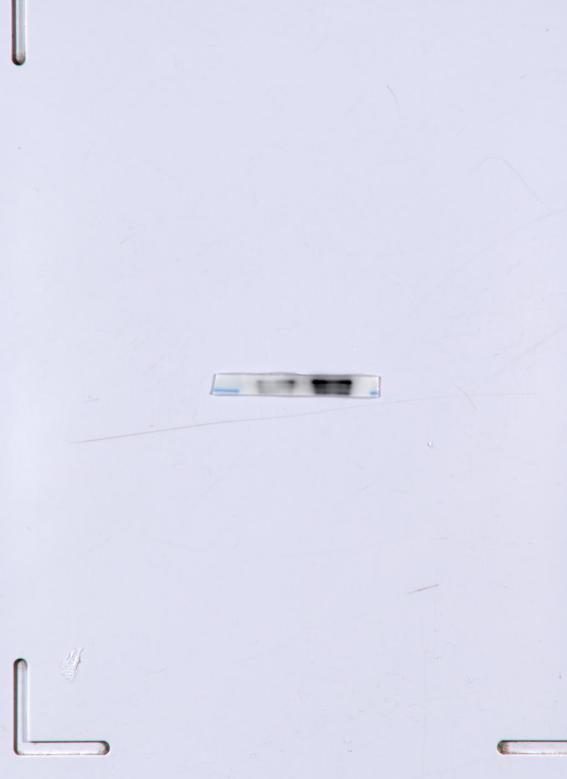
**

**
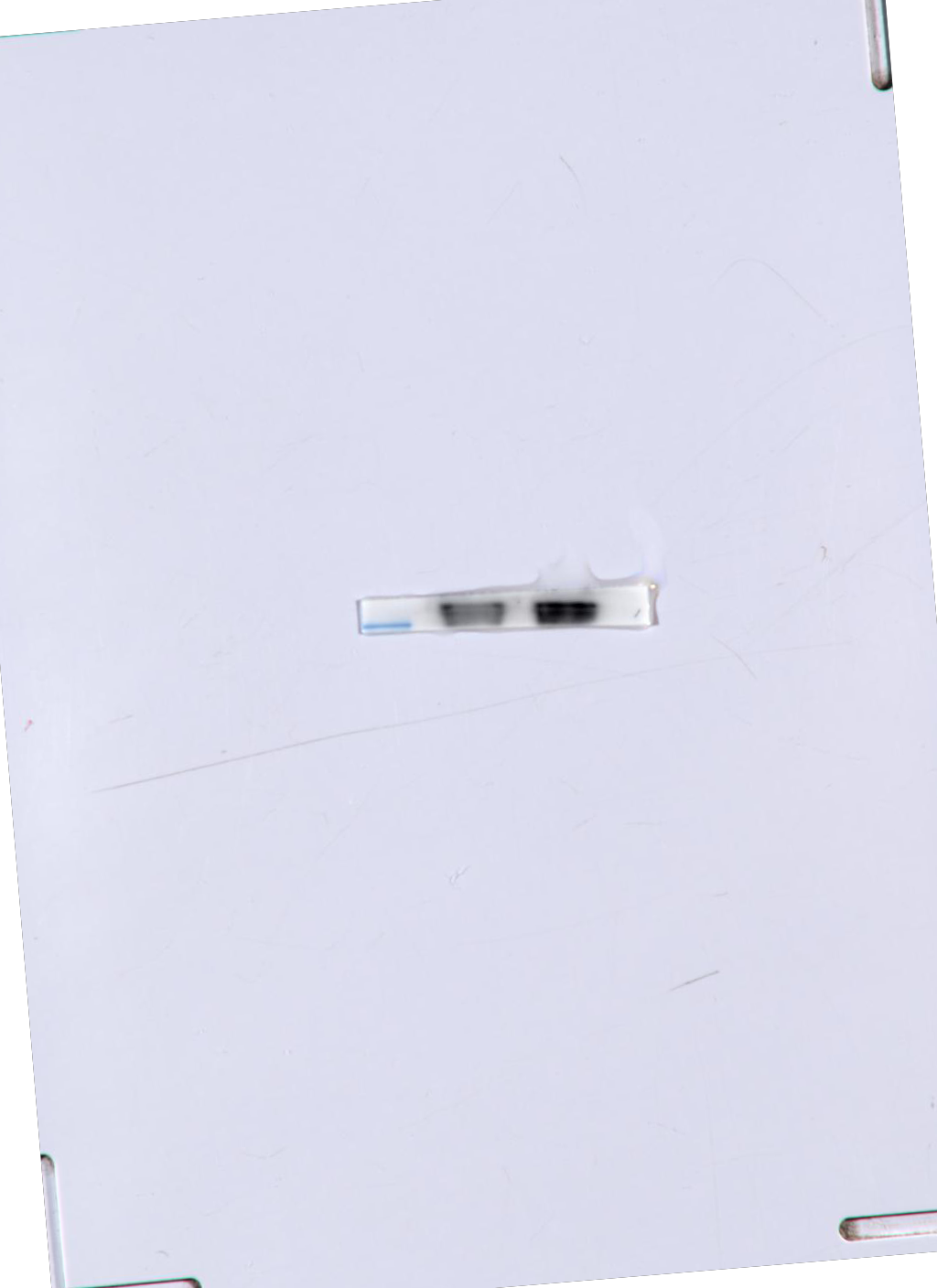
**

1. **β-actin (Duodenum, Jejunum, Ileum)**


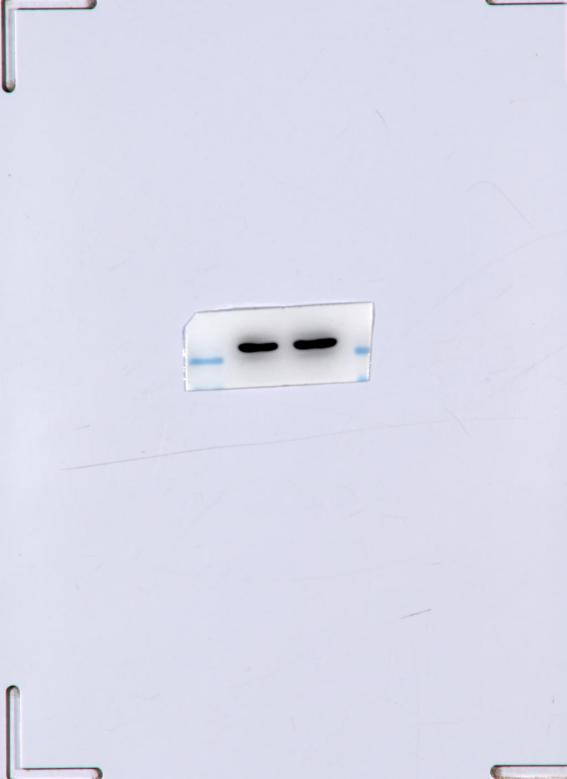

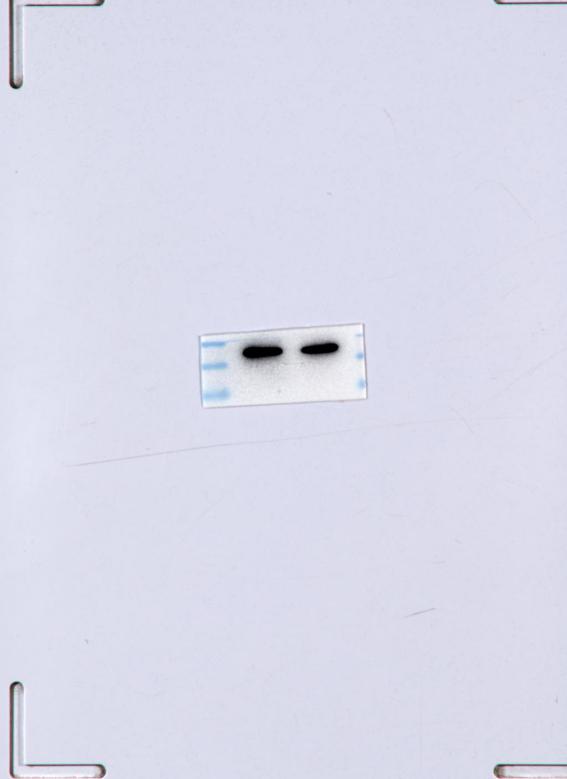


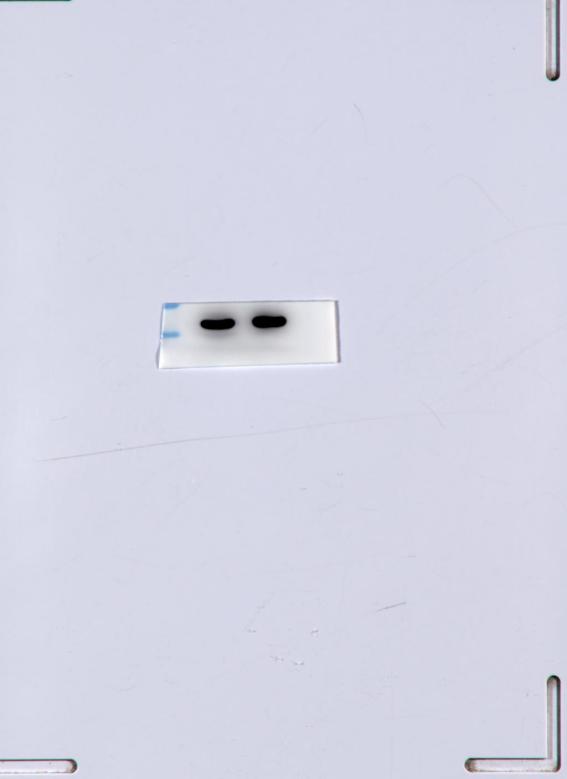


1. **Nuclearβ-catenin (Duodenum, Jejunum, Ileum)**

**
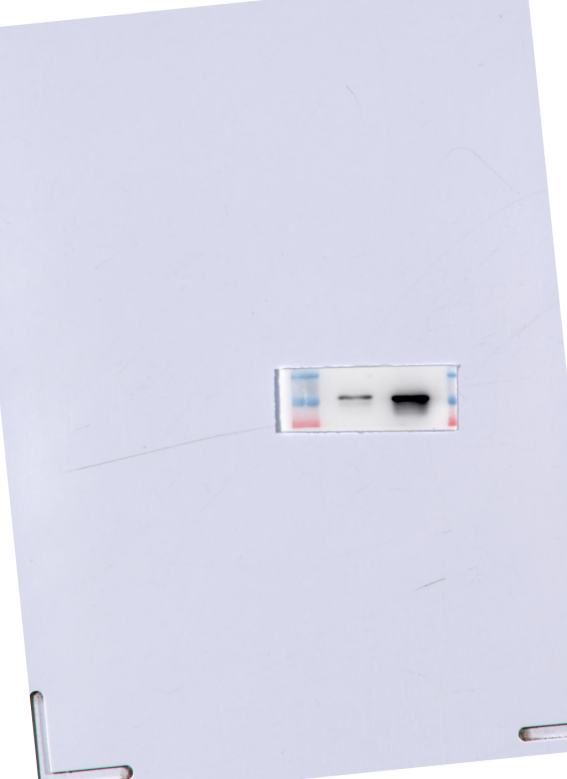
**
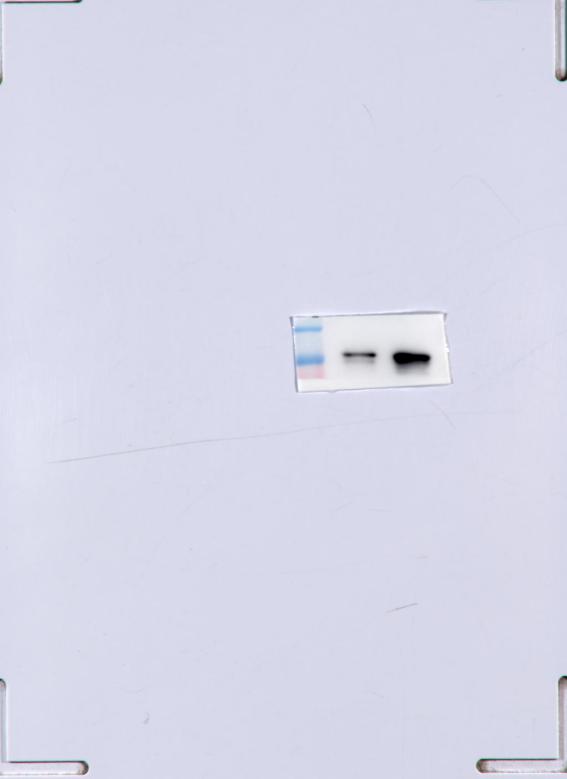


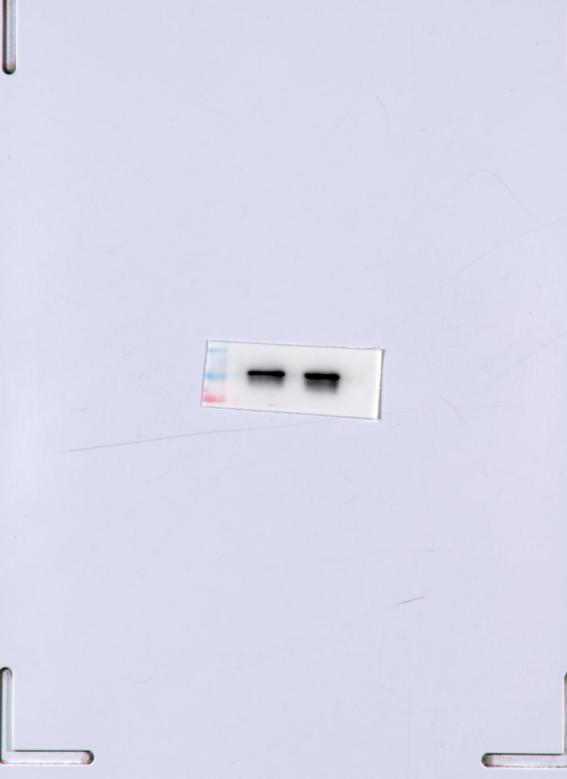


1. **Lamin B (Duodenum, Jejunum, Ileum)**

**
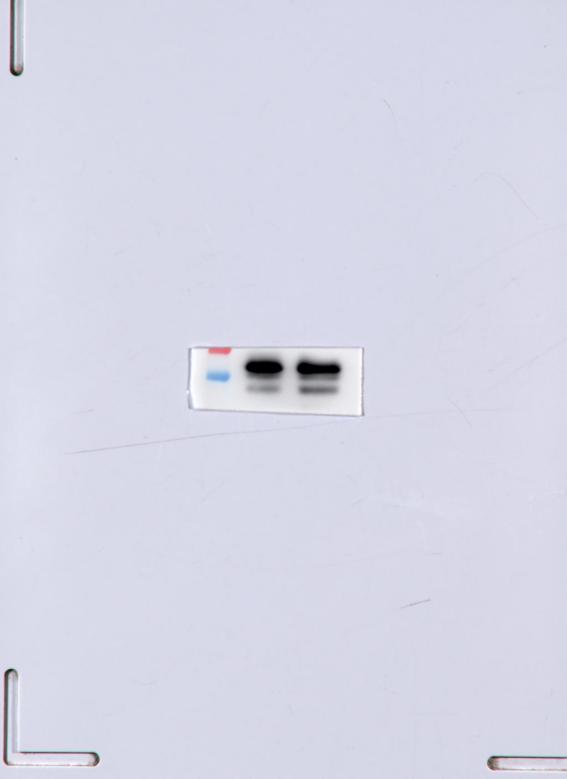
**
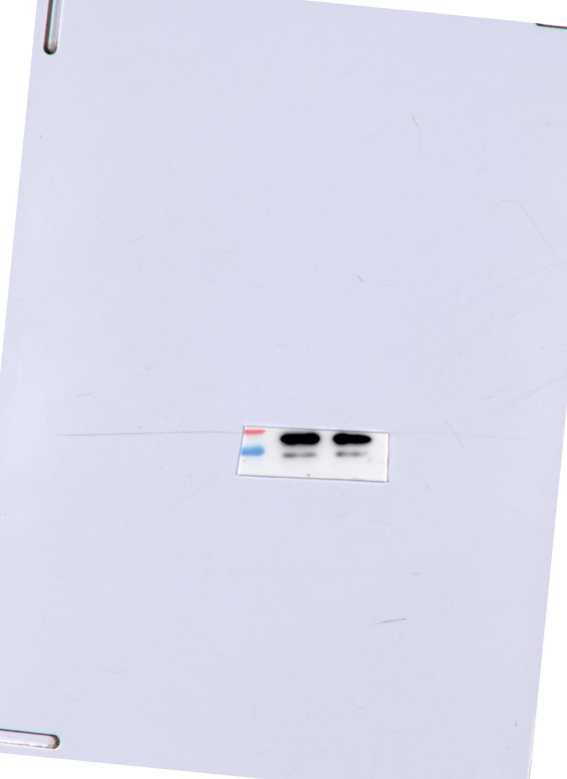

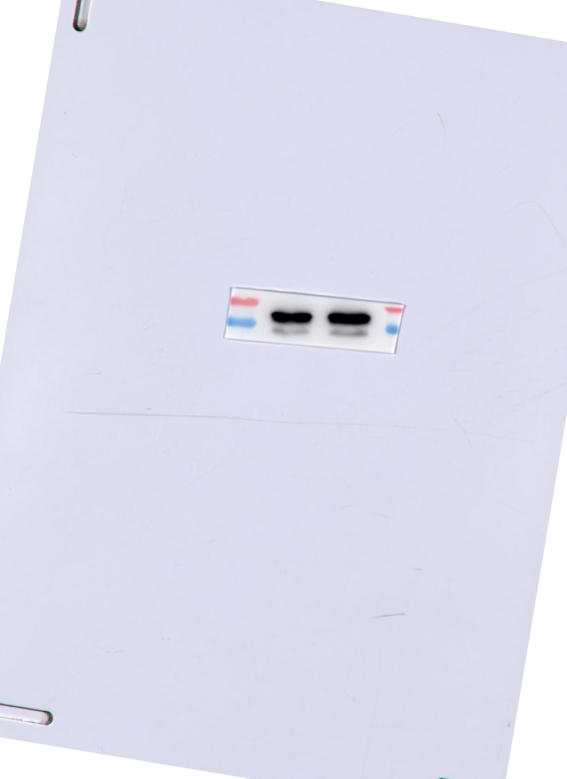


1. **Ki67 (Duodenum, Jejunum, Ileum)**

**
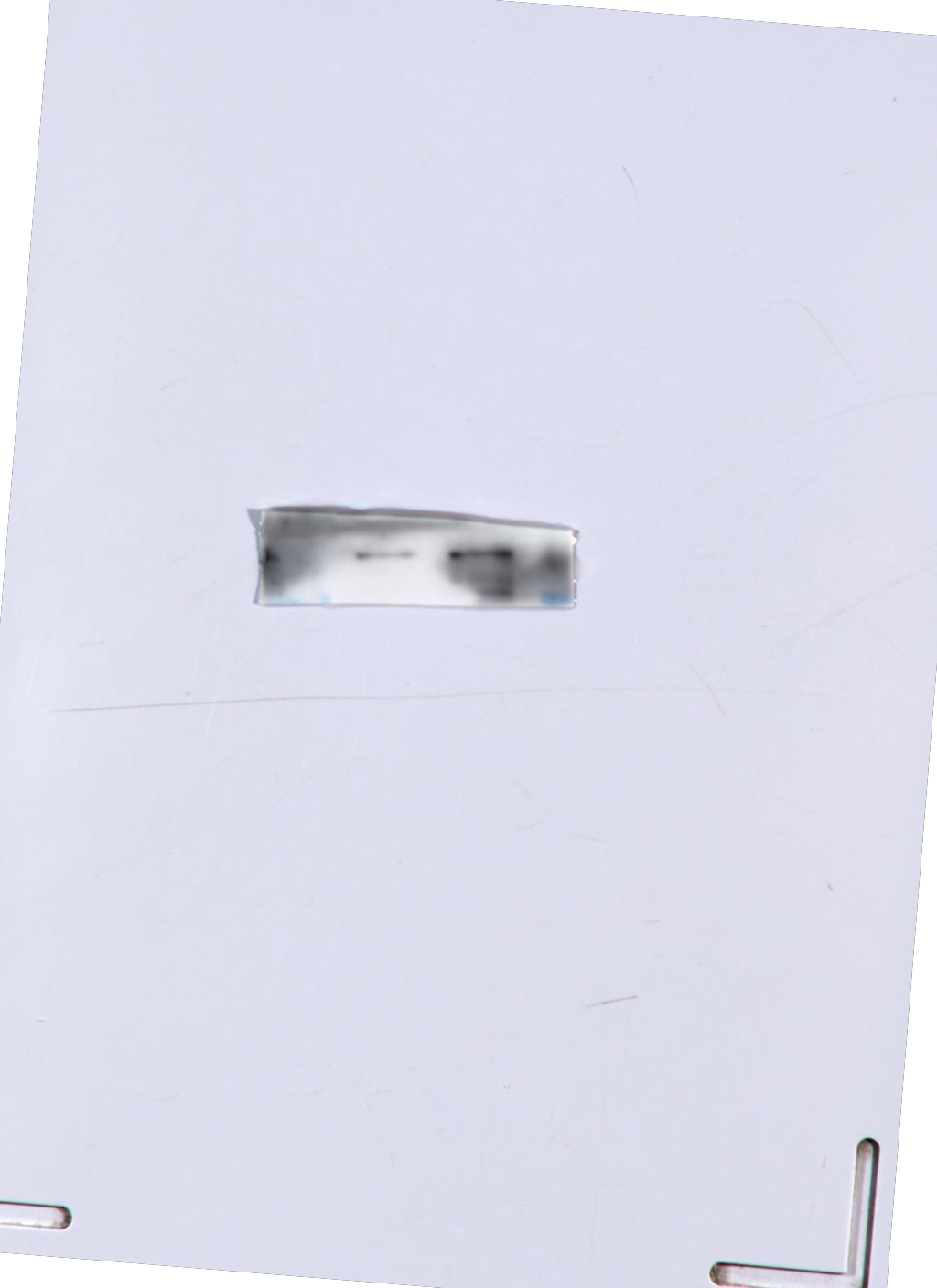
** **
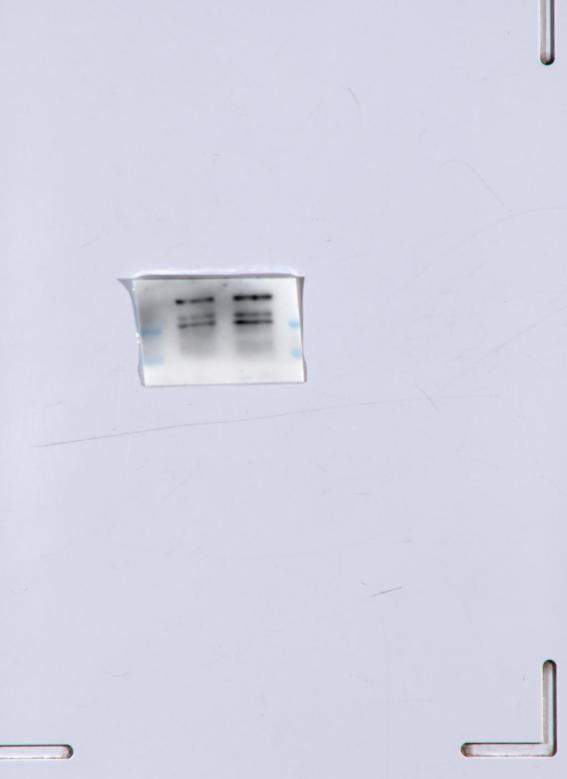
**
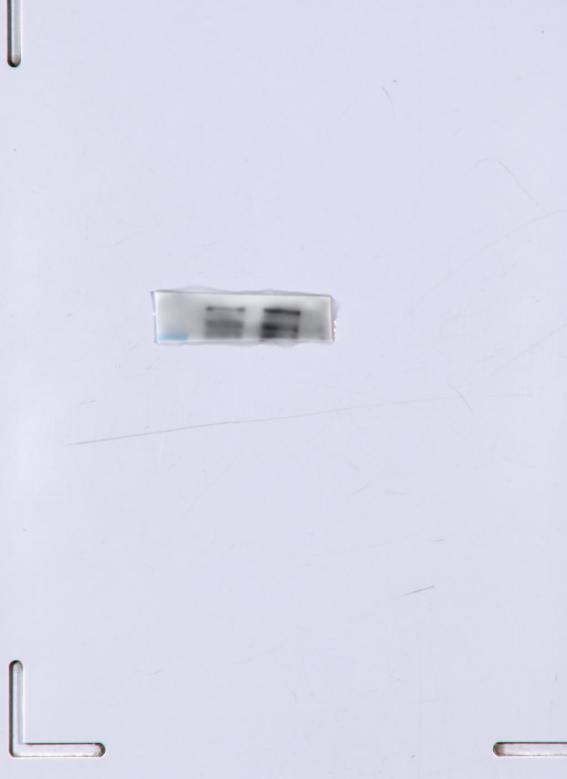


1. **Cyclin D1 (Duodenum, Jejunum, Ileum)**

**
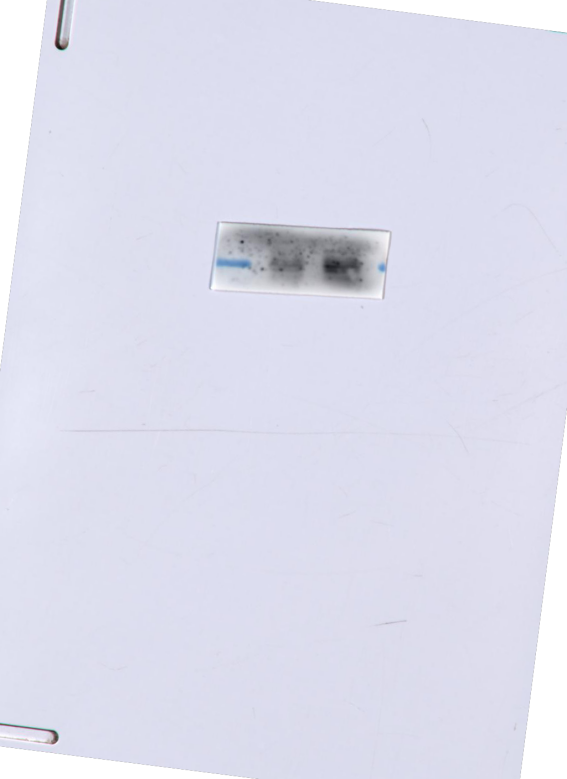
** **
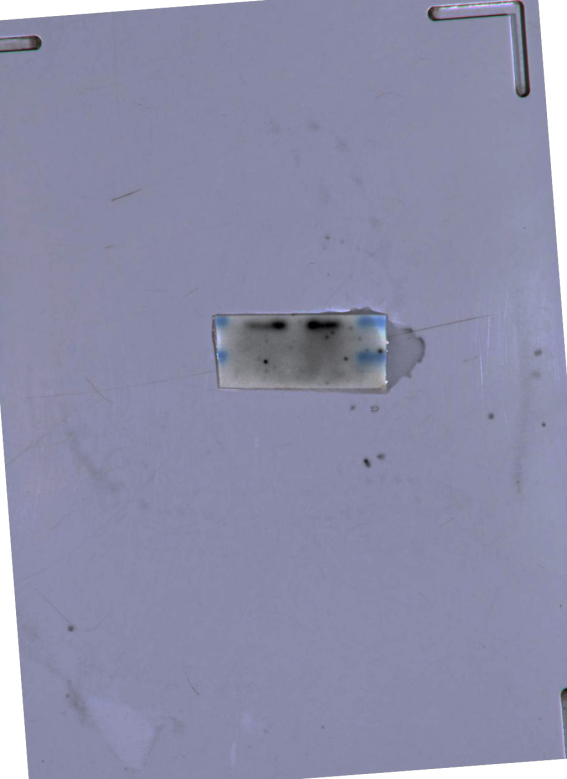
**
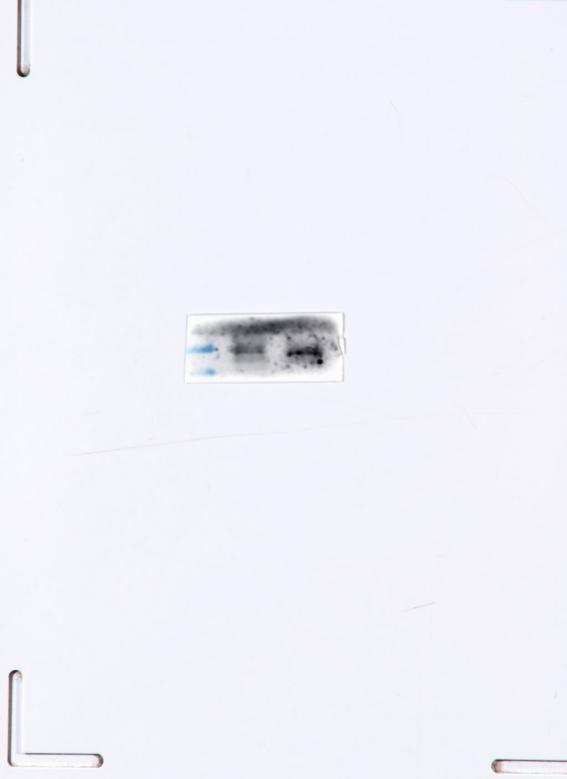


1. **Lgr5 (Duodenum, Jejunum, Ileum)**

**
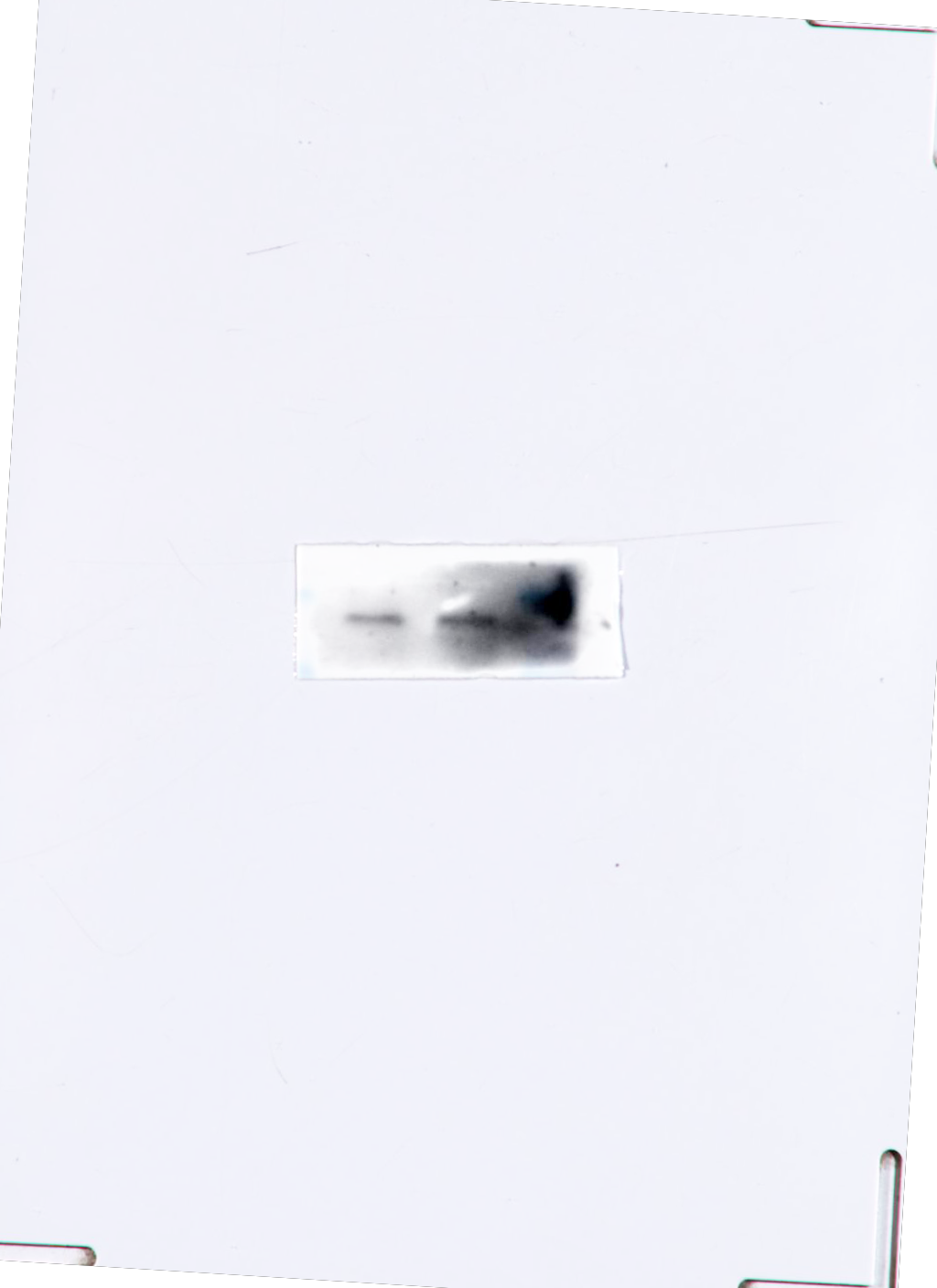

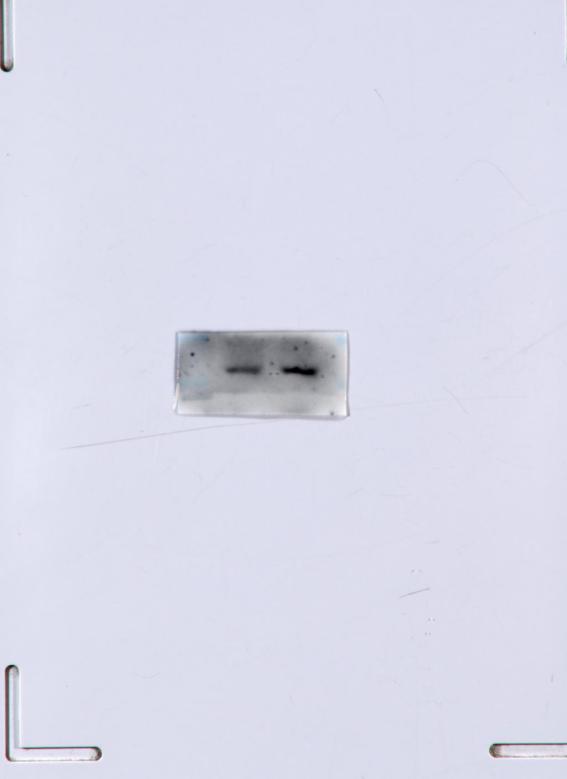
**
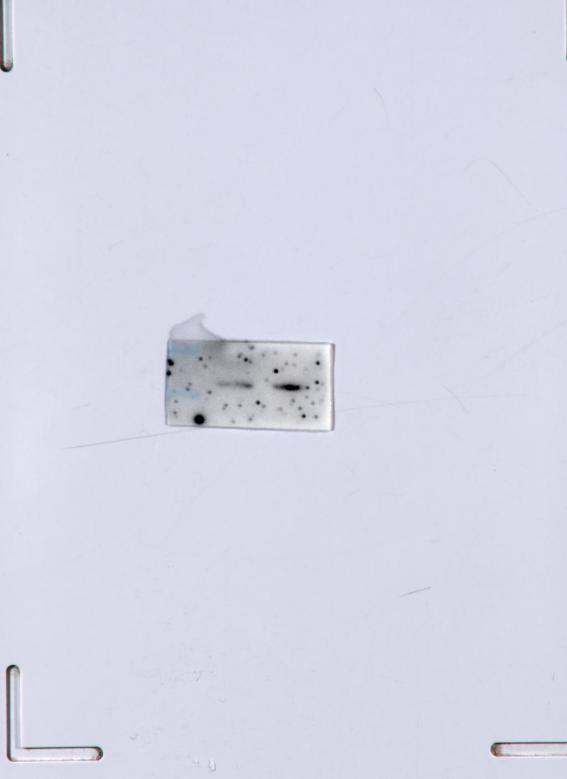


1. **β-actin (Duodenum, Jejunum, Ileum)**

**
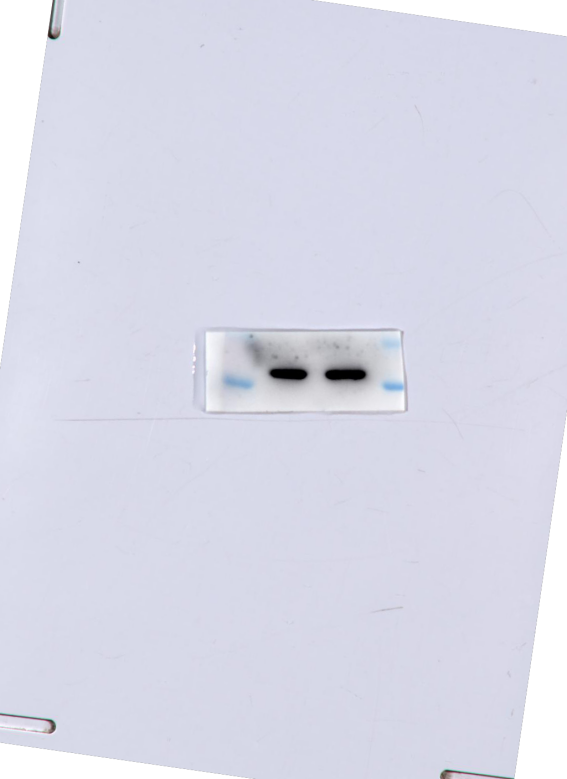
** **
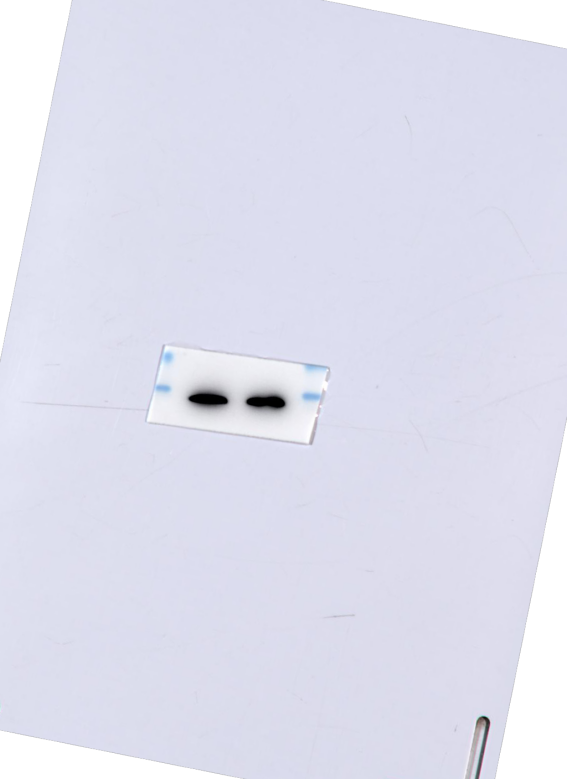
**

**
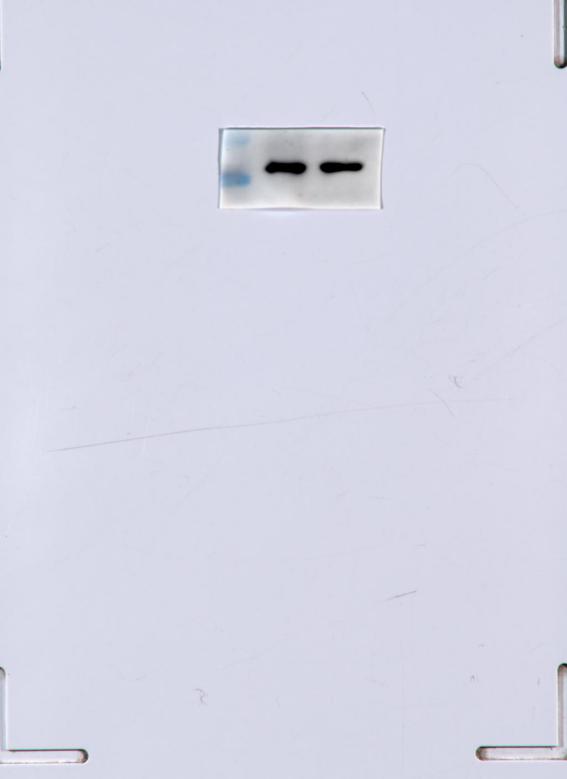
**

1. **Wnt1 (IPEC-J2)**

**
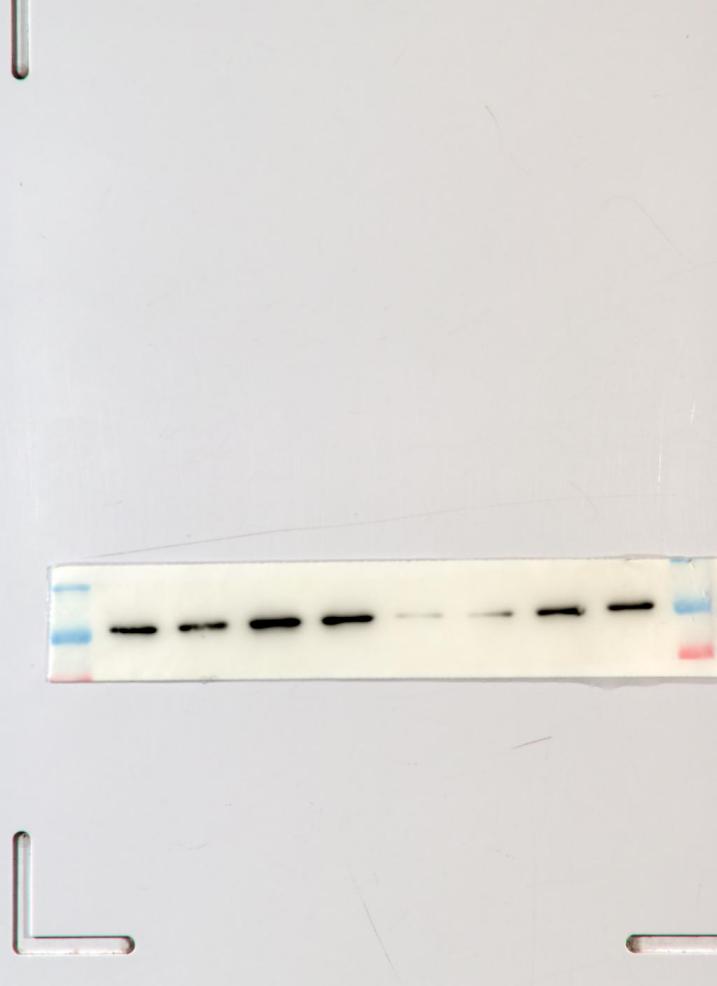
**

1. **β-catenin (IPEC-J2)**

**
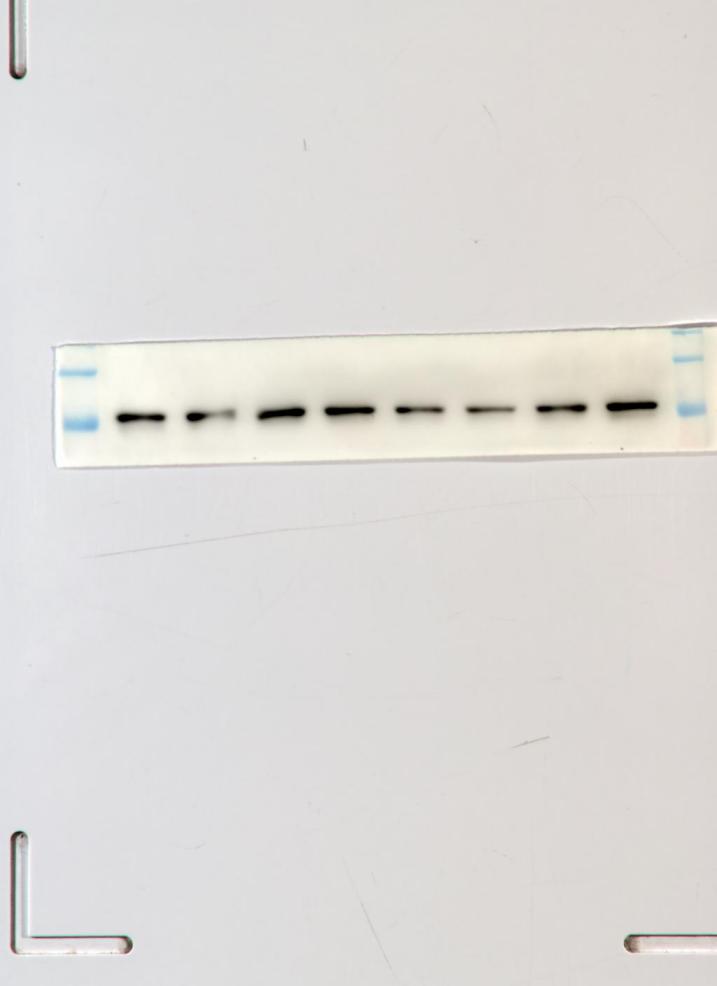
**

1. **β-actin (IPEC-J2)**

**
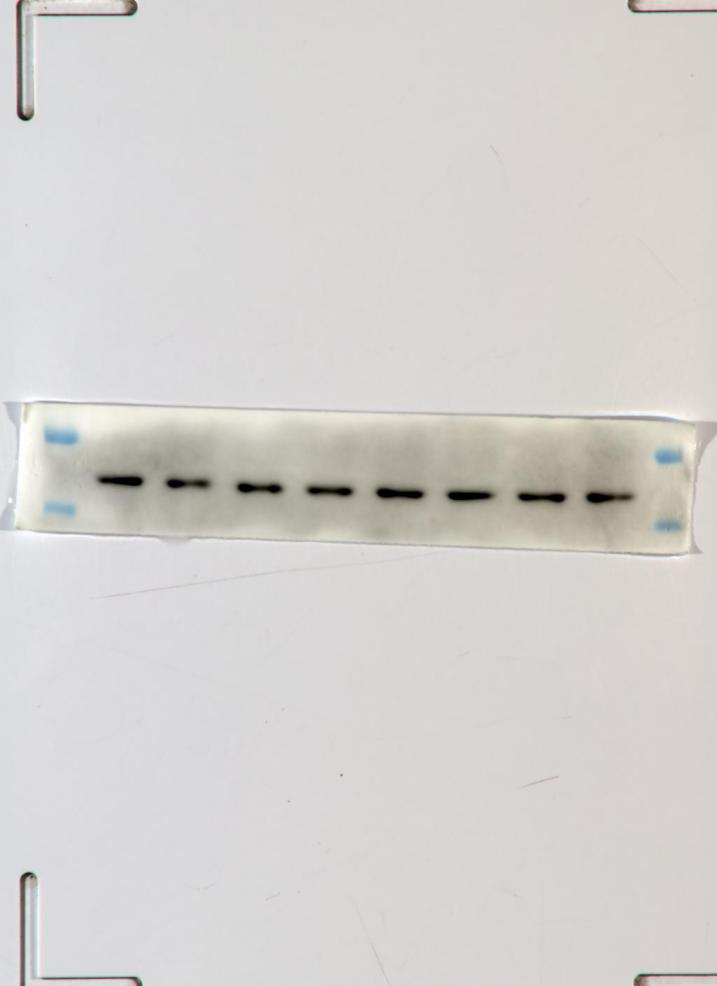
**
